# Supplementary material for: Structural basis for specific inhibition of the deubiquitinase UCHL1
Source: Nat Commun. 2022 Oct 10;13:5950. doi: 10.1038/s41467-022-33559-4 (PMC9549030; doi:10.1038/s41467-022-33559-4)
Supplement: Supplementary file 1 — Supplementary Information [file 41467_2022_33559_MOESM1_ESM.pdf]

Supplementary Information

Structural basis for specific inhibition of the deubiquitinase UCHL1

Christian Grethe[1,2,#], Mirko Schmidt[1,2,#], Gian-Marvin Kipka[1,2], Rachel O'Dea[1,2], Kai Gallant[1,2], Petra Janning[3], and Malte Gersch[1,2,*]

[1] Max Planck Institute of Molecular Physiology, Chemical Genomics Centre, Otto-Hahn-Str. 15, Dortmund, Germany

[2] TU Dortmund University, Department of Chemistry and Chemical Biology, Otto-Hahn-Str. 15, Dortmund, Germany

[3] Max Planck Institute of Molecular Physiology, Department of Chemical Biology, Otto-Hahn-Str. 11, Dortmund, Germany

[#] Authors contributed equally.

* Correspondence: malte.gersch@mpi-dortmund.mpg.de, malte.gersch@tu-dortmund.de

Contents

1. Supplementary Figures ....p. 2

2. Uncropped gels and blots .... p. 24

3. Chemical synthesis .... p. 28

   3.1 Synthesis of CG017 .... p. 29

   3.2 Synthesis of CG041 .... p. 32

   3.3 Synthesis of 3-carboxypyrrolidine-based probes and controls .... p. 34

   3.4 Synthesis of 2-carboxypyrrolidine-based probes and controls .... p. 43

   3.5 Synthesis of CG050 .... p. 49

   3.6 Synthesis of MS37 .... p. 52

   3.7 Synthesis of MS23 .... p. 55

4. Chemical characterization data .... p. 58

5. Supplementary References .... p. 72

Supplementary Figure 1

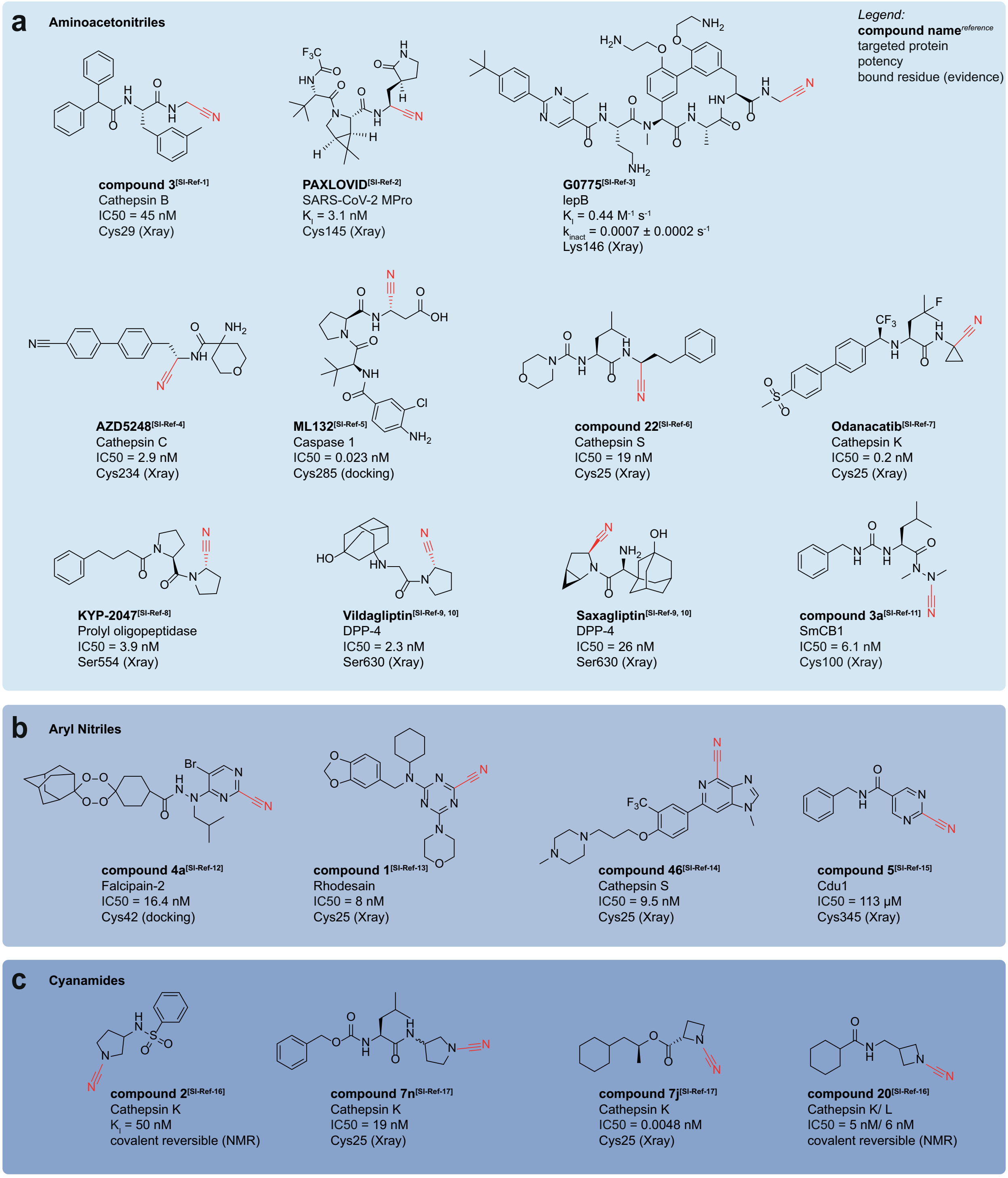

Supplementary Figure 1. Nitrile-based covalent inhibitors.

a-c. A selection of nitrile-based inhibitors was classified as aminoacetonitriles (**a**), aryl nitriles (**b**) and cyanamides (**c**). The electrophilic nitrile is shown in red. Targeted proteins, potency, bound residues, and the evidence for covalent modification are given.

Supplementary Figure 2

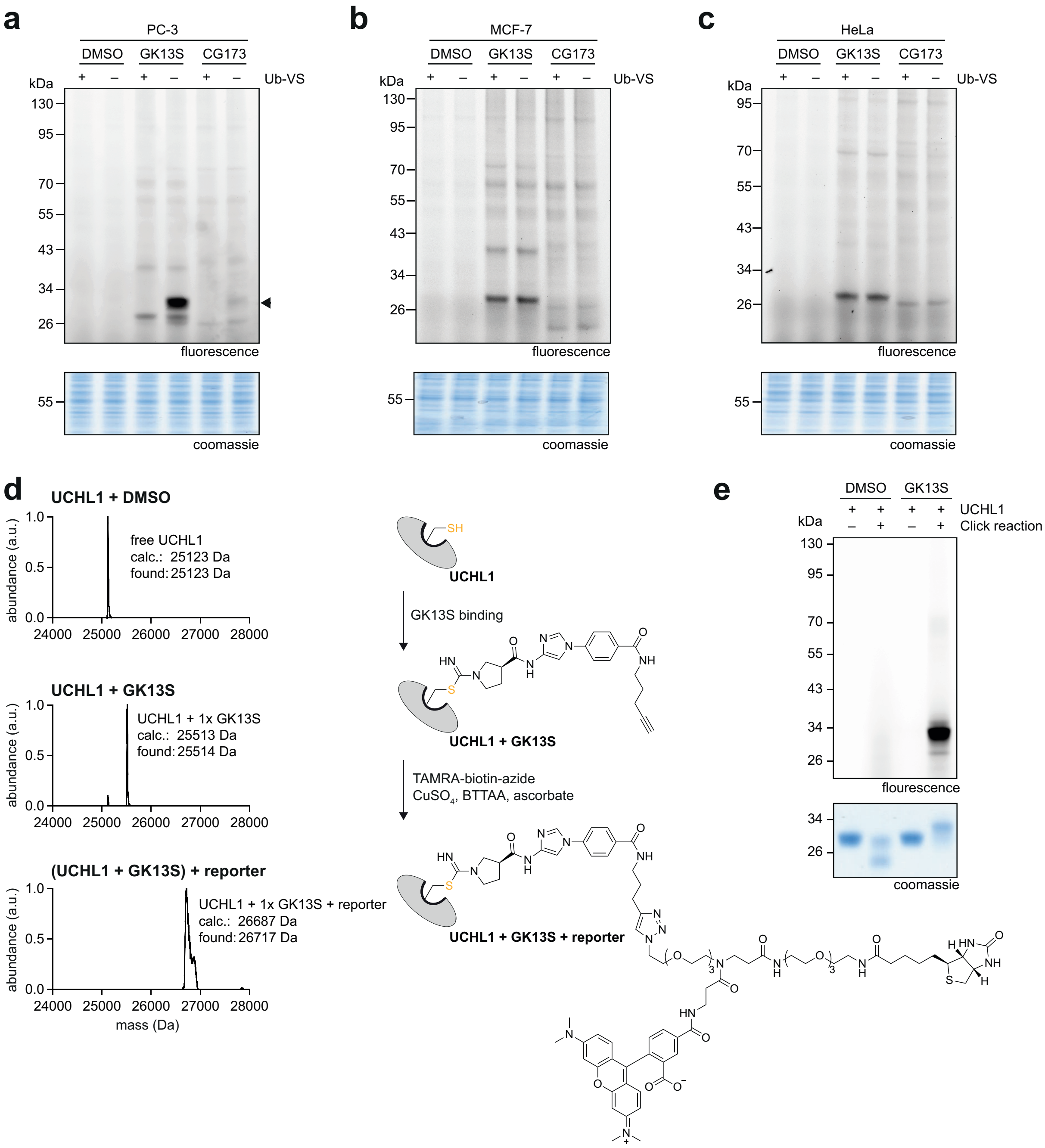

Supplementary Figure 2. Validation of activity-based protein profiling workflow.

a, b, c. Activity-based protein profiling in PC-3 (**a**), MCF-7 (**b**) and HeLa (**c**) cell lysate with indicated compounds (1 µM, 1 h) as in Fig. 1d. Proteomes were pre-treated with Ub-VS probe where shown. The black arrow indicates a Ub-VS competitive protein target in GK13S- and CG173-treated samples in PC-3 cells which is absent in the other cell lines. Uncropped versions of gels are shown in the supplementary information.

d. Intact protein mass spectra of apo UCHL1 (top), UCHL1 bound to GK13S (middle), UCHL1 bound to GK13S with the incorporated reporter molecule via Copper(I)-catalyzed azide-alkyne cycloaddition (CuAAC) (bottom, all on the left panel) and respective schematic illustrations of the observed protein species (right panel).

e. In-gel fluorescence showing the labeling of recombinant UCHL1 with a TAMRA-biotin-azide as trifunctional reporter in the presence of GK13S.

Supplementary Figure 3

a

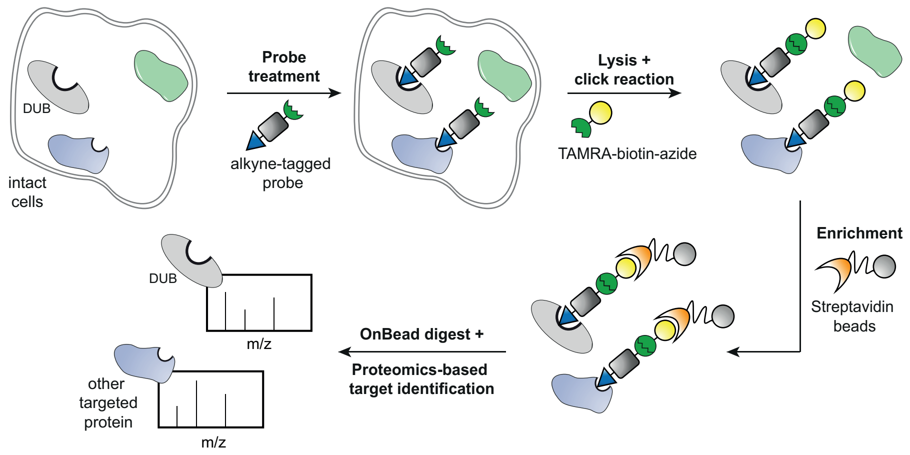

b

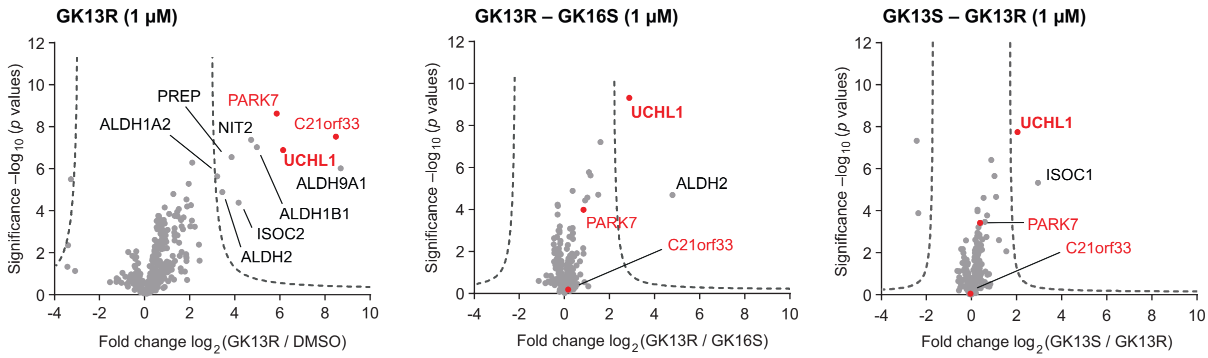

Supplementary Figure 3. Identification of cellular targets.

a. Schematic workflow of cellular activity-based protein profiling (ABPP) for target identification. Intact cells were treated with the probe for typically 24 h, and subsequently lysed. A TAMRA-biotin reporter conjugate was introduced via click chemistry. Probe-bound proteins where either analyzed by in-gel fluorescence (Fig. 2b) or subjected to proteomics-based mass spectrometry experiments (Fig. 2d, e and Supplementary Fig. 3b).

b. Proteomics-based target identification of indicated probes. Volcano plots show the relative label-free abundance ratio (fold change) of proteins between indicated samples. Compare Fig. 2e. Cells were treated for 24 h at indicated concentrations. UCHL1, PARK7 and the PARK7-homologue C21orf33 are marked in red.

Supplementary Figure 4

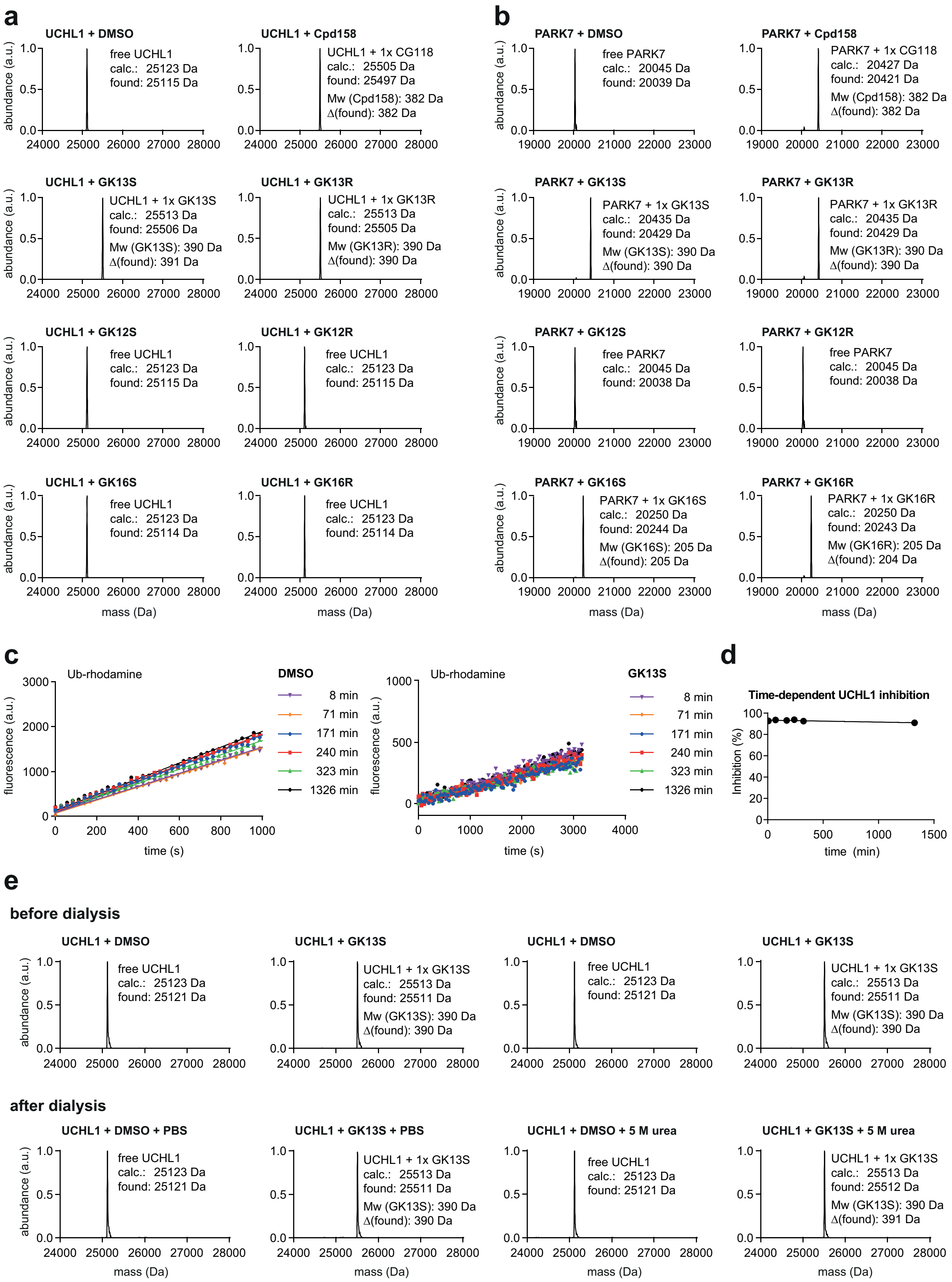

Supplementary Figure 4. *In vitro* characterization of probe binding mode.

a, **b**. Intact protein mass spectrometry data of indicated compounds binding to recombinant UCHL1 (**a**) or PARK7 (**b**). UCHL1 (3 µM) or PARK7 (3 µM) were treated with compounds (10 µM) or DMSO for 16 h. Observed masses were assigned to given species with listed expected masses. See Fig. 2a for compound structures.

c. Jump-dilution assay. UCHL1 was treated with DMSO or an excess of GK13S, then diluted 100-fold, and its activity measured in Ub-rhodamine cleavage assays after indicated timepoints.

d. Time-dependent UCHL1 inhibition based on slopes of individual time points from jump-dilution assay shown in c.

e. Dialysis dilution assay. UCHL1 was treated with DMSO or an excess of GK13S (upper panel), then diluted 5-fold with PBS or 5 M urea as indicated, dialyzed overnight into PBS and its protein mass was determined (lower panel).

Supplementary Figure 5

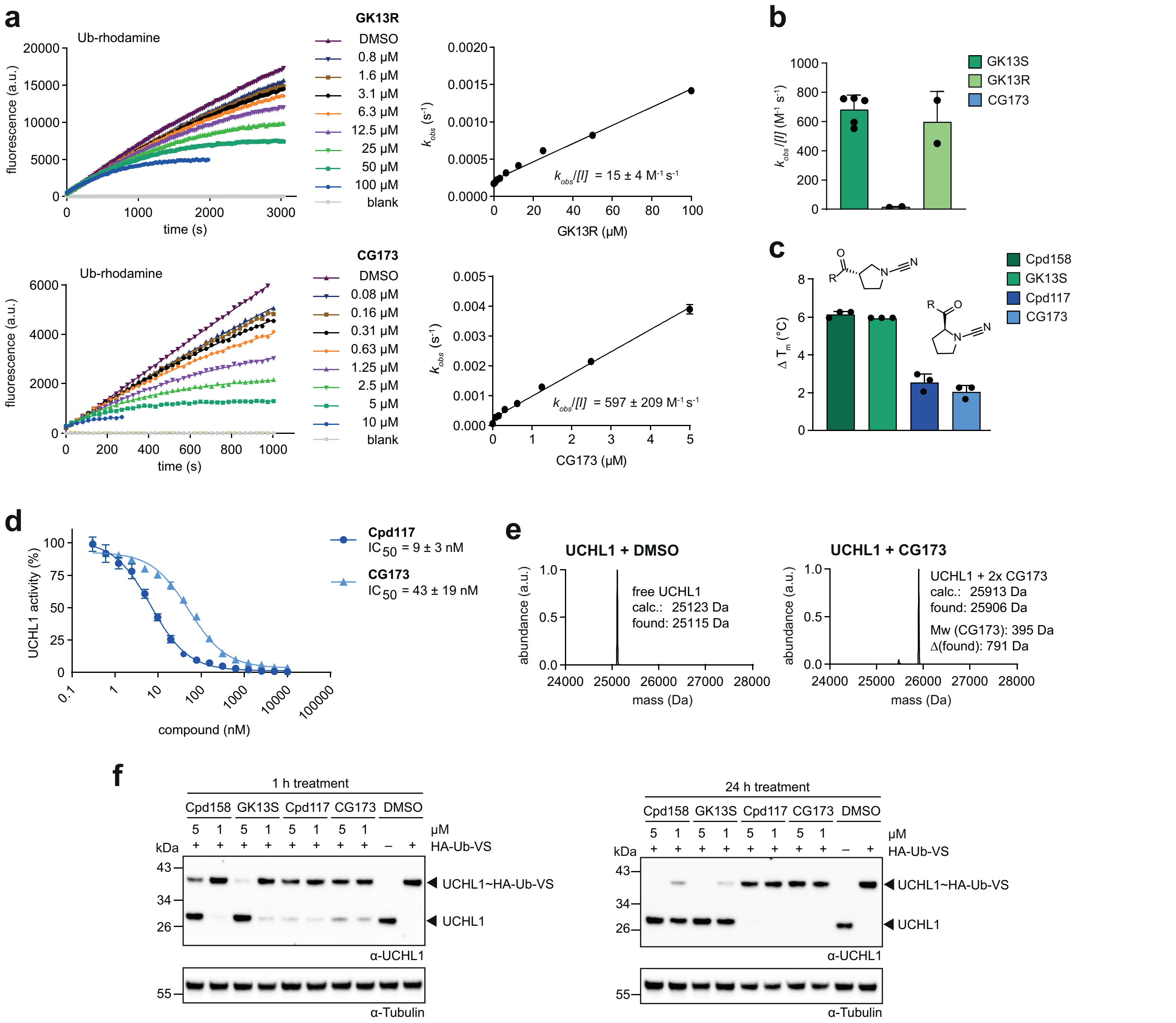

Supplementary Figure 5. Comparison of 2- and 3-carboxy-*N*-cyanopyrrolidine UCHL1 inhibitors.

a. $k_{obs}/[I]$ kinetic assay of GK13R (upper part) and CG173 (lower part) binding to UCHL1 at indicated concentrations. $k_{obs}/[I]$ values were calculated from 2 independent experiments.

b. Comparison of $k_{obs}/[I]$ values of indicated compounds. Related experiments are shown in Fig. 3c and panel a. Values are plotted as mean ± standard deviation from 5 (GK13S) or 2 (GK13R and CG173) independent experiments.

c. Comparison of changes in UCHL1 protein melting temperatures (ΔT_m) after binding to 3- and 2-carboxy-*N*-cyanopyrrolidine-based compounds. See Fig. 1a for complete chemical structures. Values are given as mean ± standard deviation from 3 technical replicates.

d. Inhibitory potencies of indicated compounds were determined from Ubiquitin rhodamine cleavage assays. Compare Fig. 3b. IC_{50} values were determined from 2 independent experiments and are given as mean ± standard deviation.

e. Intact protein mass spectrometry data of compound CG173 covalently binding recombinant UCHL1 twice. UCHL1 (3 µM) was treated with compound (10 µM) or DMSO for 16 h, identical conditions as in Supplementary Fig. 4a.

f. Comparison of inhibitory potency on cellular UCHL1 for 3- and 2-carboxy-*N*-cyanopyrrolidine-based compounds. Same workflow as in Fig. 3e. Uncropped versions of gels and blots are shown in the supplementary information.

Supplementary Figure 6

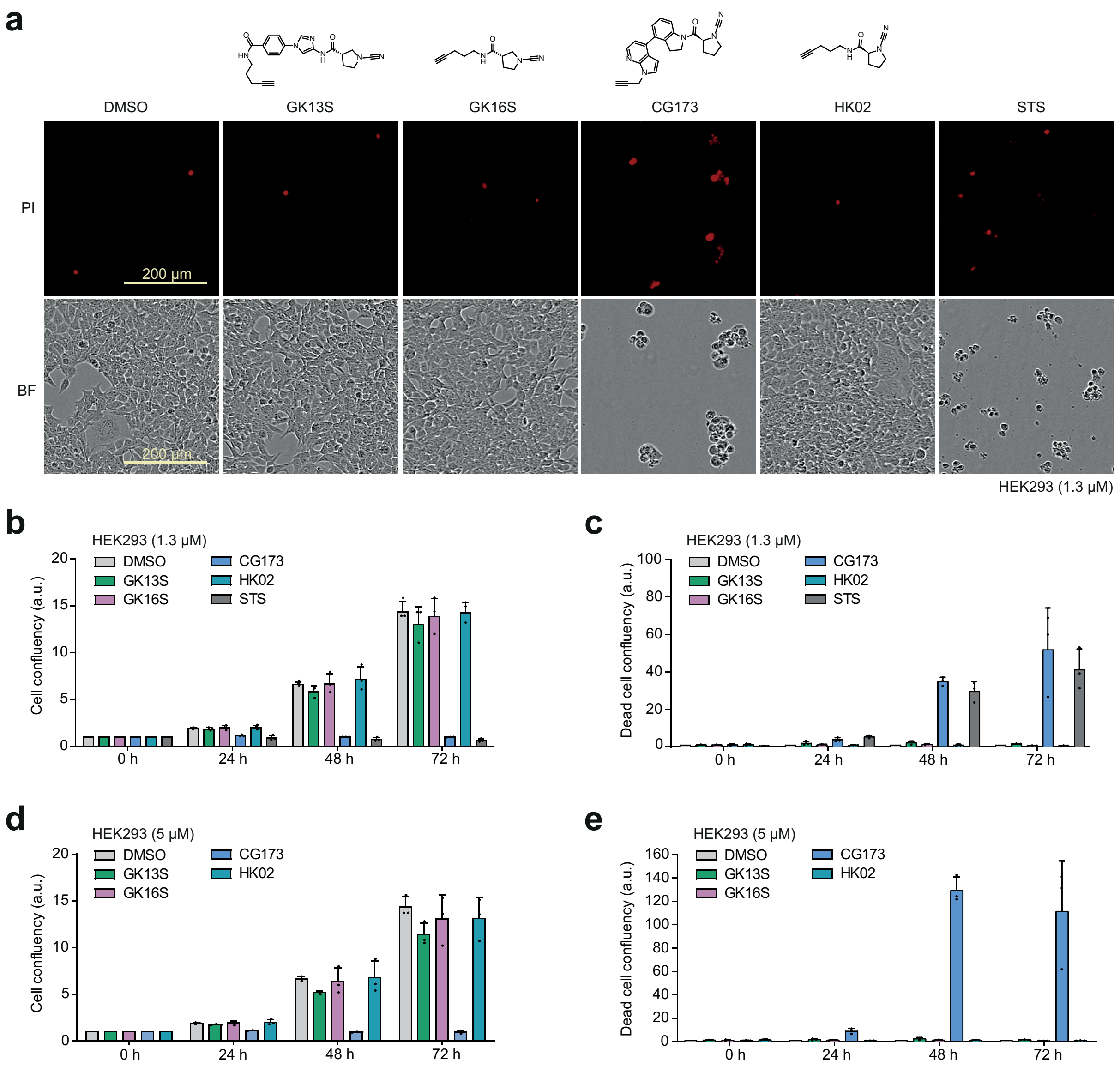

Supplementary Figure 6. 2-, but not 3-carboxy-*N*-cyanopyrrolidines induce apoptosis in HEK293 cells.

a. Representative microscopy images of HEK293 cells taken 72 h after treatment with 1.3 µM of the indicated compounds.

b. Quantification of HEK293 cell confluency observed at 24, 48 and 72 h post treatment with 1.3 µM of the indicated compounds. Confluency is normalized to that at 0 h. Data are shown as means of N = 3 independent experiments. Error bars represent standard deviation.

c. Quantification of HEK293 propidium iodide (PI) positive cells observed at 24, 48 and 72 h post treatment with 1.3 µM of the indicated compounds. Confluency is normalized to DMSO at each time point. PI positive cell confluency is adjusted to overall cell confluency. Data are shown as means of N = 3 independent experiments. Error bars represent standard deviation.

d. Quantification of HEK293 confluency after treatment with 5 µM of the indicated compounds as in b. Data are shown as means of N = 3 independent experiments. Error bars represent standard deviation.

e. Quantification of HEK293 PI positive cells observed at 24, 48 and 72 h post treatment with 5 µM of the indicated compounds as in c. Data are shown as means of N = 3 independent experiments. Error bars represent standard deviation.

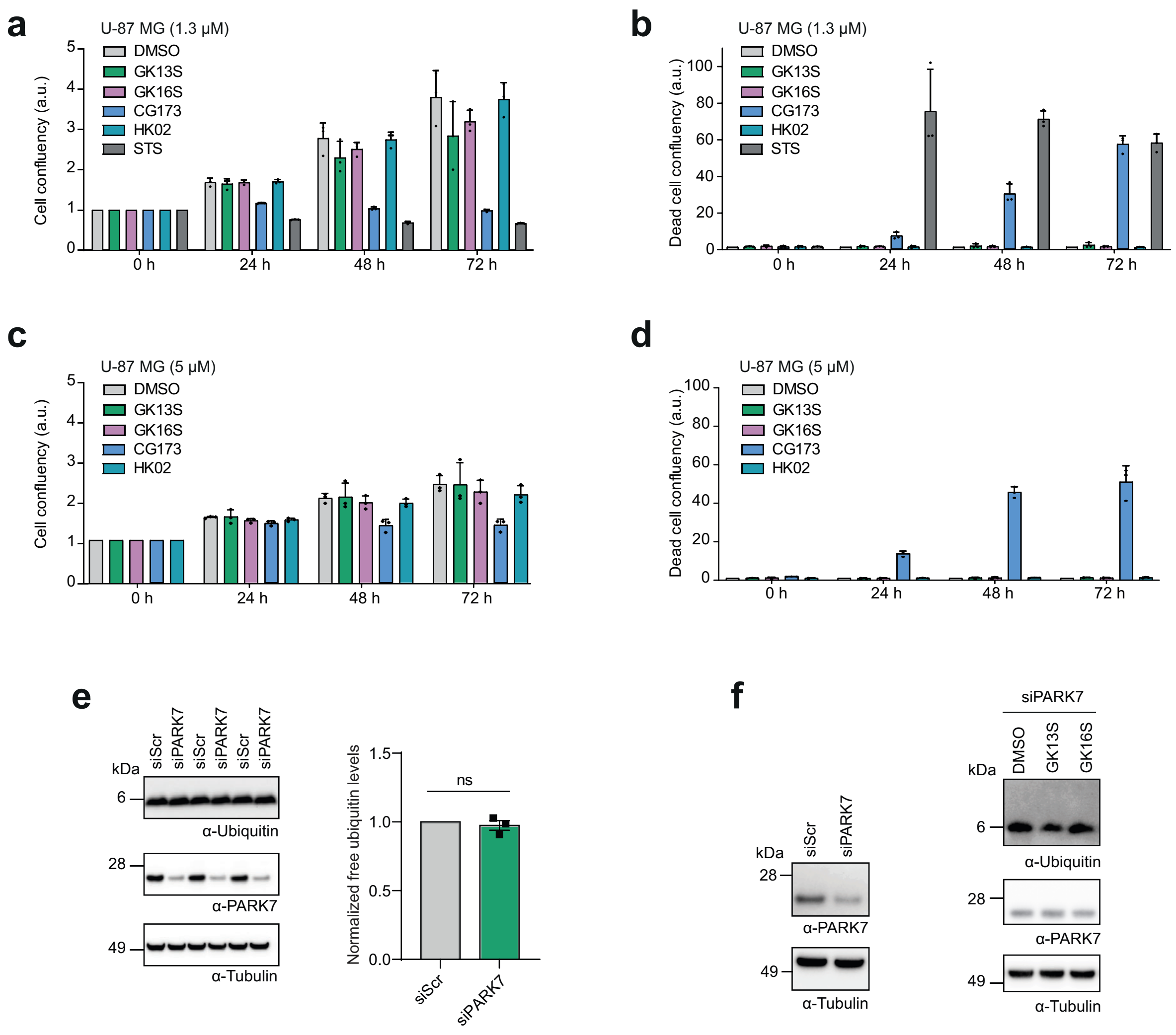
a
U-87 MG (1.3 μM)
DMSO
GK13S
GK16S
CG173
HK02
STS
Cell confluency (a.u.)
0 h
24 h
48 h
72 h
b
U-87 MG (1.3 μM)
Dead cell confluency (a.u.)
c
U-87 MG (5 μM)
d
U-87 MG (5 μM)
e
siScr
siPARK7
kDa
α-Ubiquitin
α-PARK7
α-Tubulin
Normalized free ubiquitin levels
ns
f
siPARK7
DMSO
GK13S
GK16S

Supplementary Figure 7. 2-, but not 3-carboxy-*N*-cyanopyrrolidines induce apoptosis in U-87 MG cells.

a. Quantification of U-87 MG confluency observed at 24, 48 and 72 h post treatment with 1.3 µM of the indicated compounds. Confluency is normalized to that observed at 0 h. Data are shown as means of N = 3 independent experiments. Error bars represent standard deviation.

b. Quantification of U-87 MG PI positive cells observed at 24, 48 and 72 h post treatment with 1.3 µM of the indicated compounds. Confluency is normalized to that observed for DMSO at each time point. PI positive cell confluency is adjusted to overall cell confluency. Data are shown as means of N = 3 independent experiments. Error bars represent standard deviation.

c. Quantification of U-87 MG confluency observed at 24, 48 and 72 h post treatment with 5 µM of the indicated compounds as in a. Data are shown as means of N = 3 independent experiments. Error bars represent standard deviation.

d. Quantification of U-87 MG PI positive cells observed at 24, 48 and 72 h post treatment with 5 µM of the indicated compounds as in b. Data are shown as means of N = 3 independent experiments. Error bars represent standard deviation.

e. Western blots showing that depletion of PARK7 in U-87 MG does not change monoubiquitin levels. Quantitation as in Fig. 4e. N = 3, values are plotted as mean ± standard error of the mean. Statistical significance was analyzed using a one sample, two-tailed *t*-test compared to the mean of ‘1’ as set for the siScr samples. ns, not significant. Uncropped versions of gels and blots are shown in the supplementary information.

f. Western blots showing effective depletion of PARK7 in U-87 MG cells (left panel). Samples were treated with DMSO for 48 h to exactly mimic the conditions used for experiments shown in Fig. 4e. Western blots showing monoubiquitin levels in PARK7-depleted U-87 MG cells treated with GK13S or GK16S (right panel). Compare Fig. 4e.

Supplementary Figure 8

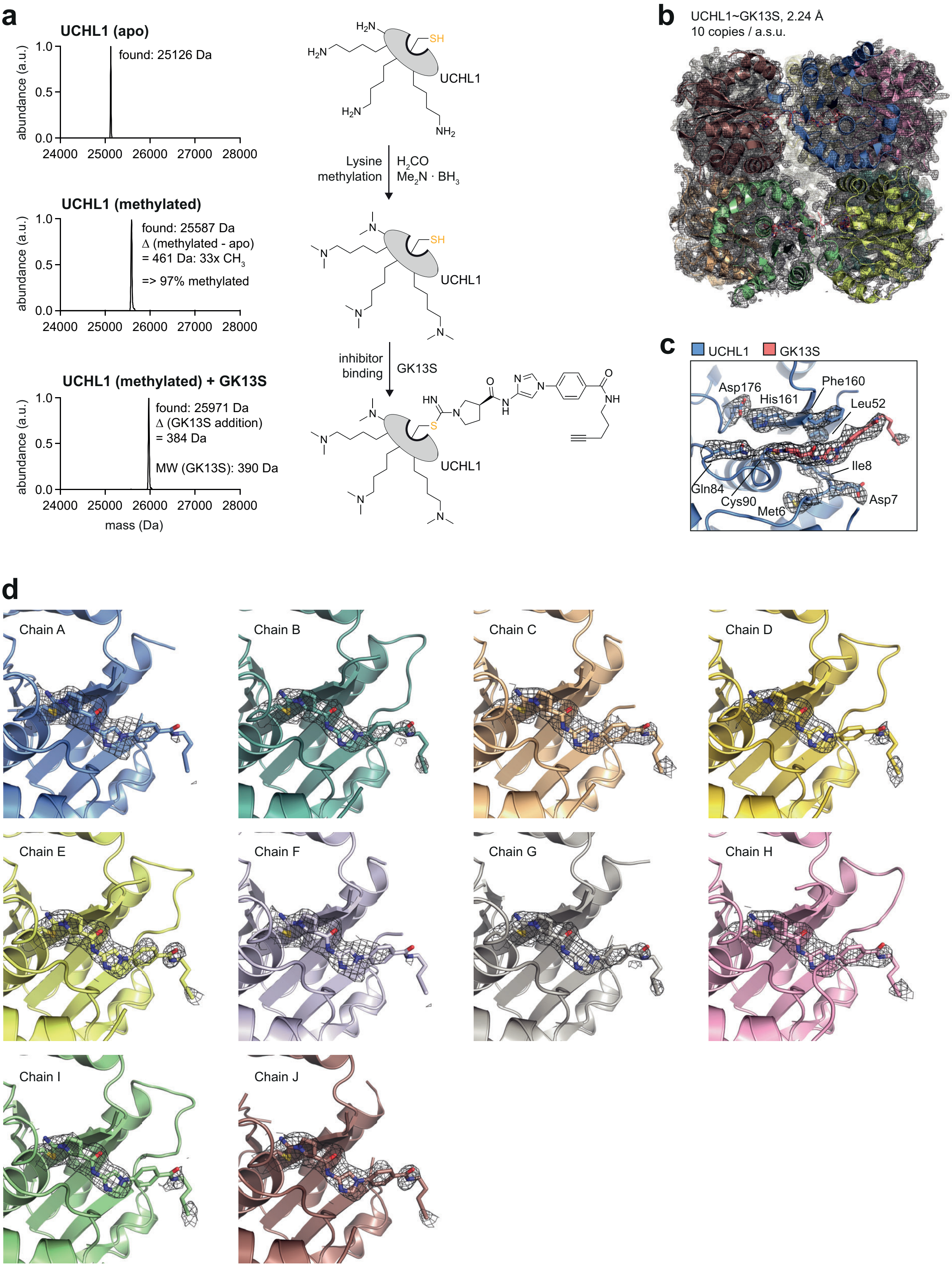

Supplementary Figure 8. Lysine methylation of UCHL1 enabled crystallization of GK13S-bound protein.

a. Lysine methylation of the crystallized human UCHL1 construct (residues 1-223 with N-terminal GS-linker). Left panel: Intact protein mass spectrometry data of apo UCHL1, methylated UCHL1 and GK13S-bound methylated UCHL1. Right panel: Corresponding schematic representations of the methylation workflow and compound binding.

b. Asymmetric unit (a.s.u.) of the solved UCHL1~GK13S structure including all 10 copies. The individual chains are shown in cartoon representation with the electron density map overlayed in grey and contoured at 0.8 σ corresponding to the weighted $|2F_0\text{-}F_C|$ electron density.

c. Close-up view of the UCHL1~GK13S binding pocket in chain C. GK13S and important surrounding amino acids are shown in stick representation with the corresponding electron density as in b.

d. Individual cartoon representations of all 10 copies of the asymmetric unit of UCHL1~GK13S. UCHL1 is shown in cartoon representation. GK13S is shown as sticks with the electron density map as in b and c.

Supplementary Figure 9

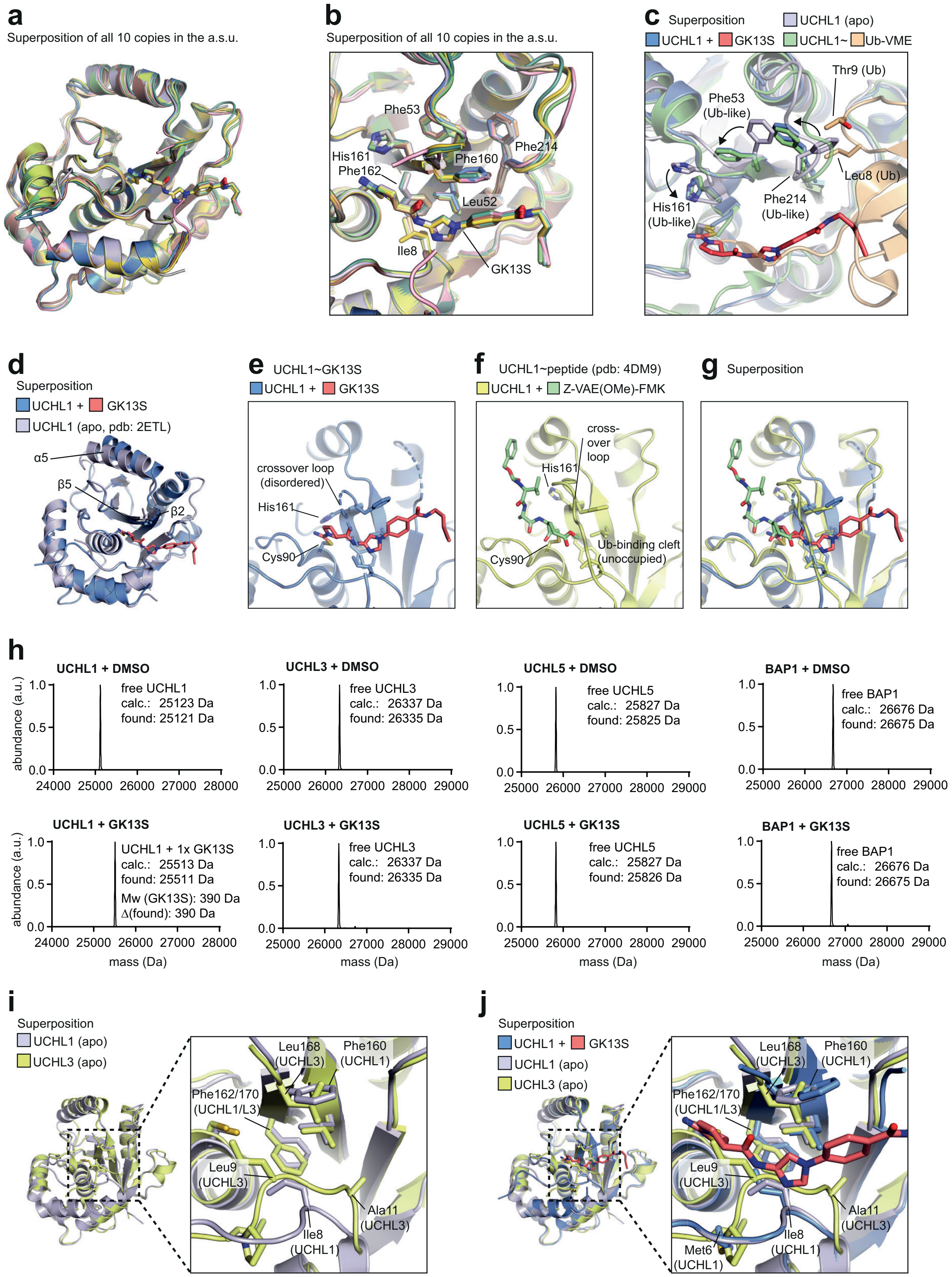

Supplementary Figure 9. Structural analysis of specific binding of GK13S to UCHL1.

a, b. Superposition of all 10 copies within the asymmetric unit of the UCHL1~GK13S structure showing the entire protein (a) or the compound binding site (b). Labeled residues and the compound are shown as sticks, demonstrating that the same binding mode is observed in all copies.

c. Superposition of UCHL1~GK13S with UCHL1 apo (pdb: 2ETL) and UCHL1~Ub-VME (pdb: 3KW5). An allosteric relay composed of Phe214, Phe53 and catalytic His161 was described previously[18] to activate the catalytic triad of UCHL1 through engaging the Leu8 loop of ubiquitin binding when bound to the S1 site. GK13S triggers the same conformational change. Indicated residues are shown as sticks.

d. Superposition of UCHL1~GK13S with UCHL1 apo (pdb: 2ETL). Secondary structure elements changed by GK13S binding are labelled.

e. Close-up view of the GK13S binding site in UCHL1. The covalently targeted active-site cysteine 90 and catalytic histidine 161 are shown as sticks.

f. Close-up view of UCHL1 bound to a peptide-fluoromethyl ketone (FMK), obtained from the soaking of apo crystals (pdb: 4DM9). The peptide Z-VAE(OMe)-FMK and the catalytic Cys90 and His161 are shown as sticks. Crossover loop and unoccupied Ubiquitin binding cleft are indicated.

g. Superposition of e and f. Both compounds bind in opposite orientations. GK13S establishes contacts with residues of the same UCHL1 copy to which it is covalently bound. The phenyl ring in the CbZ group of the peptide inhibitor is coordinated through a crystal contact to a neighboring copy.

h. Intact protein mass spectrometry data of indicated UCH catalytic domains, treated with GK13S where indicated. Enzyme (3 μM) was treated with compound (10 μM) or DMSO for 1 h.

i, j. Superposition of UCHL1~GK13S with apo UCHL1 (pdb: 2ETL) and apo UCHL3 (pdb: 1UCH) and close-up view of the binding pocket. Labelled residues are shown as sticks. Compare Fig. 5a.

Supplementary Figure 10

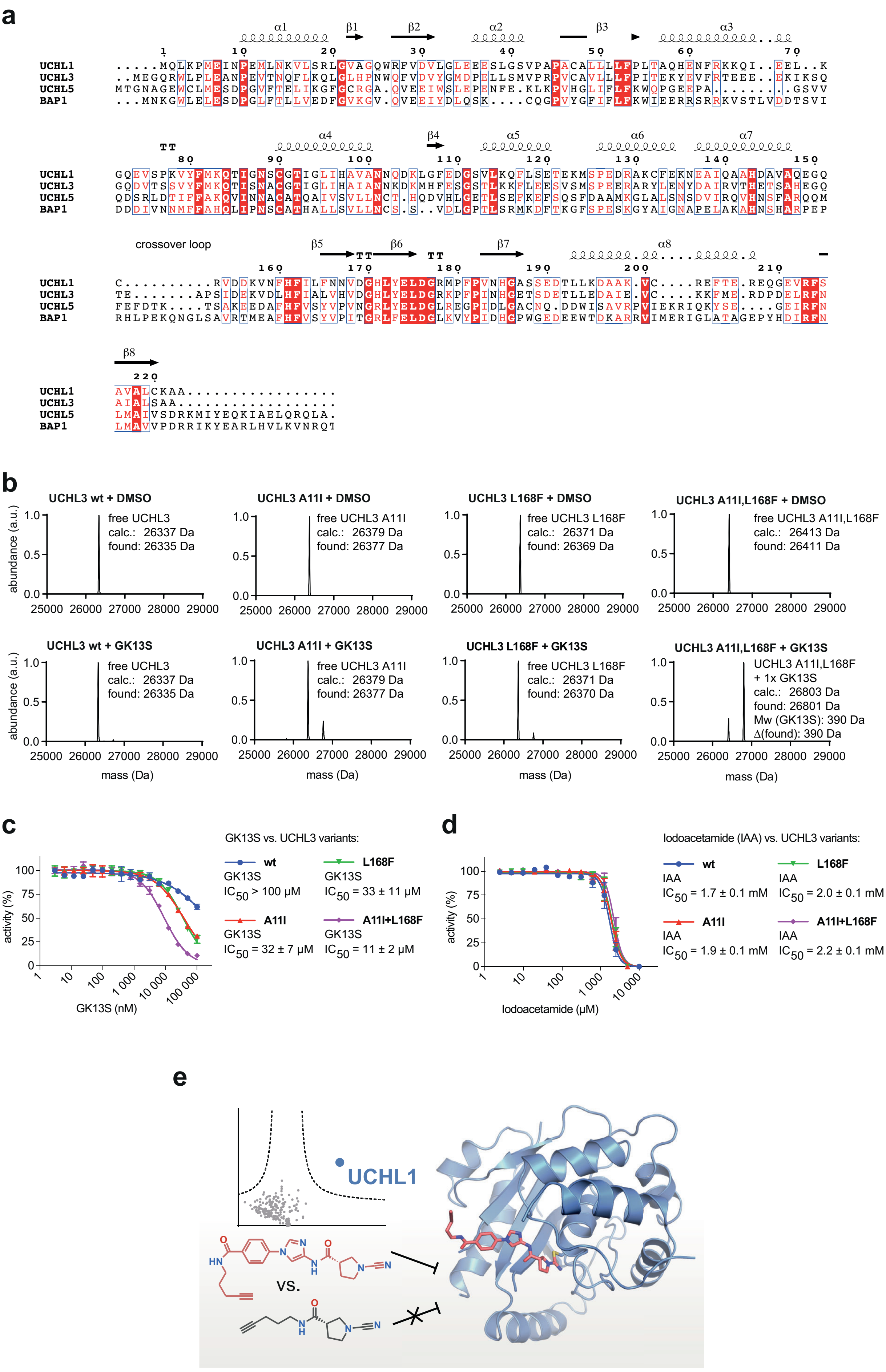

Supplementary Figure 10. Inhibition by GK13S is not driven by chemical reactivity, but by specific recognition by UCH enzymes.

a. Full sequence alignment of human Ubiquitin C-terminal Hydrolase (UCH) family members. Secondary structure assignments are based on the UCHL1~GK13S structure.

b. Intact protein mass spectrometry data of indicated UCHL3 catalytic domains, treated with GK13S where indicated. Enzyme (3 µM) was treated with compound (10 µM) or DMSO for 1 h.

c. Inhibitory potencies of GK13S against indicated UCHL3 catalytic domains determined from Ubiquitin rhodamine cleavage assays. IC_{50} values were determined from 3 independent experiments. Data are shown from a representative experiment as mean ± standard deviation.

d. Inhibitory potencies of iodoacetamide (IAA) against indicated UCHL3 catalytic domains determined from Ubiquitin rhodamine cleavage assays. IC_{50} values were determined from 3 independent experiments. Values are plotted as mean ± standard error from 3 independent experiments.

e. Graphical summary of this manuscript.

Supplementary Figure 11

PARK7 glyoxalase mechanism

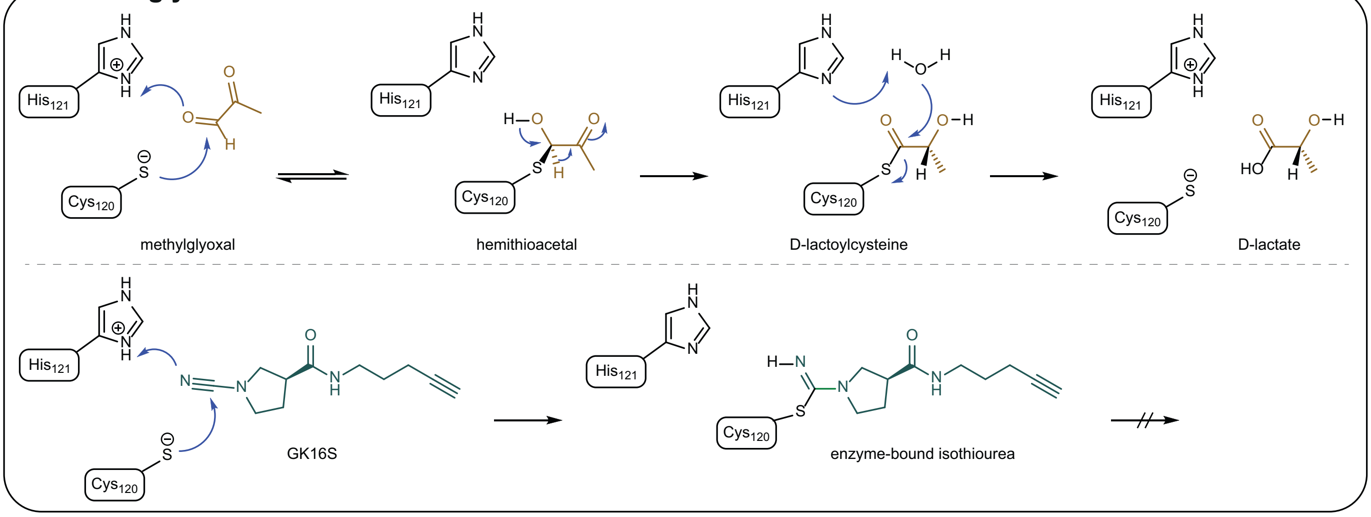

PARK7 in glycolysis

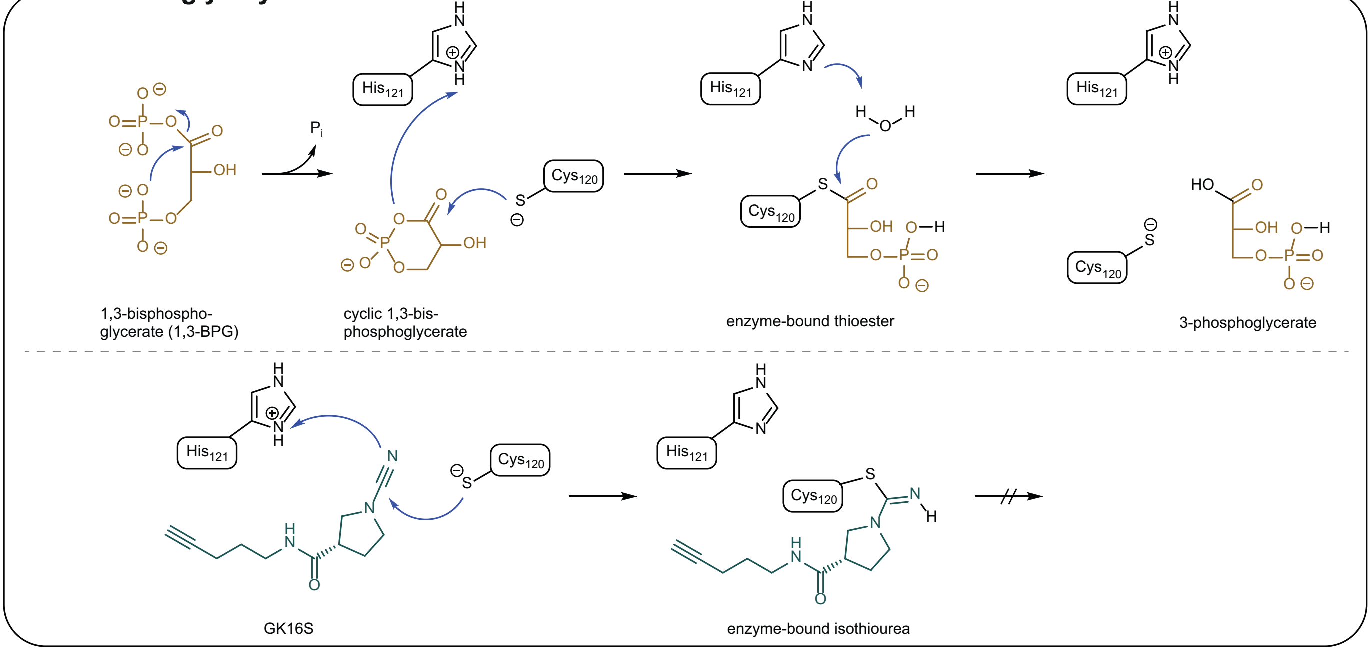

C21orf33 glutamine amidotransferase mechanism

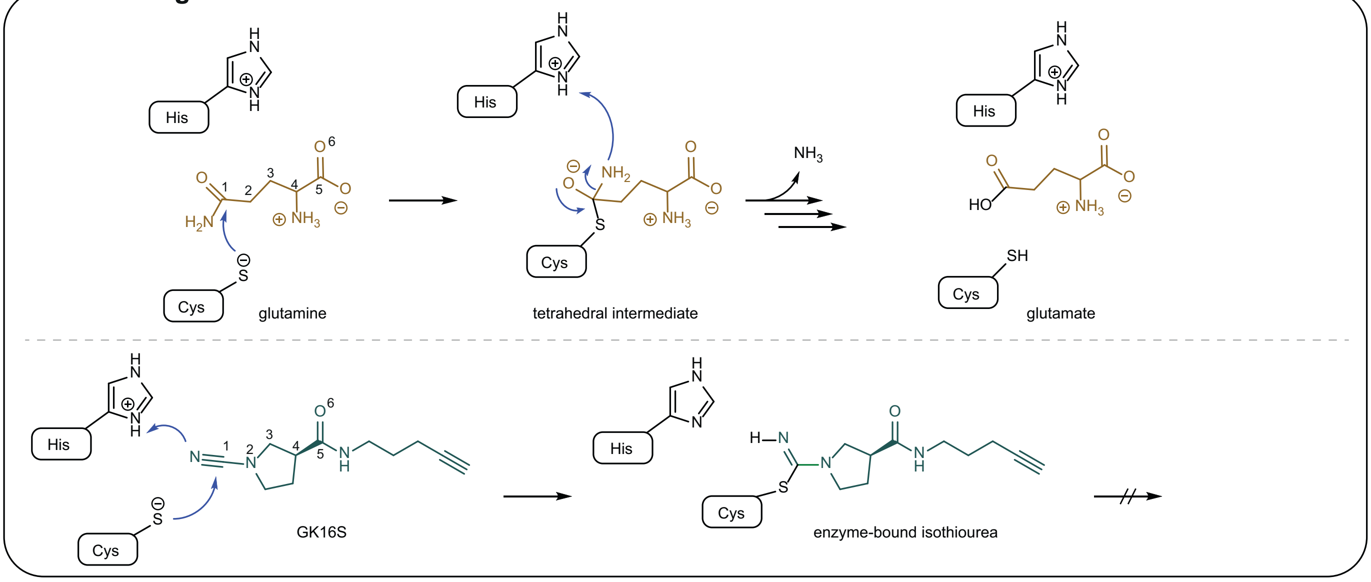

Supplementary Figure 11. Potential chemical basis for cross-reactivity of cyanamide-containing probes with PARK7 and C21orf33/GATD3.

PARK7 glyoxylase mechanism.[19] Methylglyoxal is attacked by the active site cysteine forming a reversible hemithioacetal. Proton transfer and subsequent hydrolysis lead to release of D-lactate and free catalytic cysteine.

PARK7 in glycolysis.[20] PARK7 prevents phosphoglycerate modifications on proteins from reactive cyclic-1,3-phosphoglycerate by forming a reversible enzyme-bound thioester, followed by hydrolysis to yield 3-phosphoglycerate. Minimal probe GK16S is proposed to be attacked by the active site cysteine of PARK7 in a similar manner, forming an irreversible isothiourea.

C21orf33 glutamine amidotransferase mechanism.[21] Deamidation of glutamine proceeds via a tetrahedral intermediate and release of ammonia. GK16S displays a similar size and chemical characteristics as the glutamine substrate (indicated by the numbers) and thereby is proposed to be recognized by C21orf33 in a similar manner.

2. Uncropped gels and blots

Fig. 1d

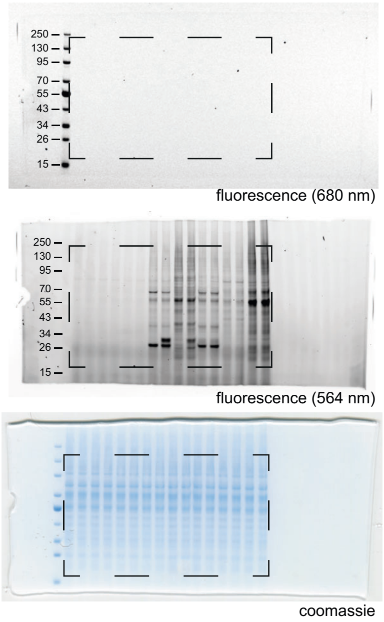

Fig. 2b

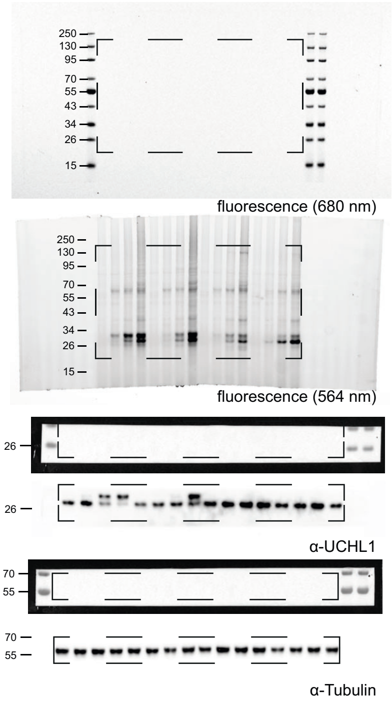

Fig. 2c

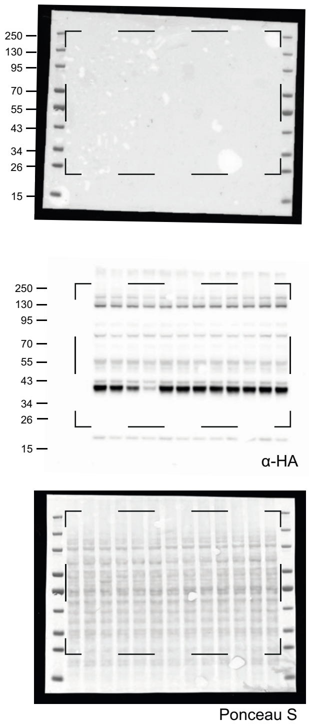

Fig. 2f

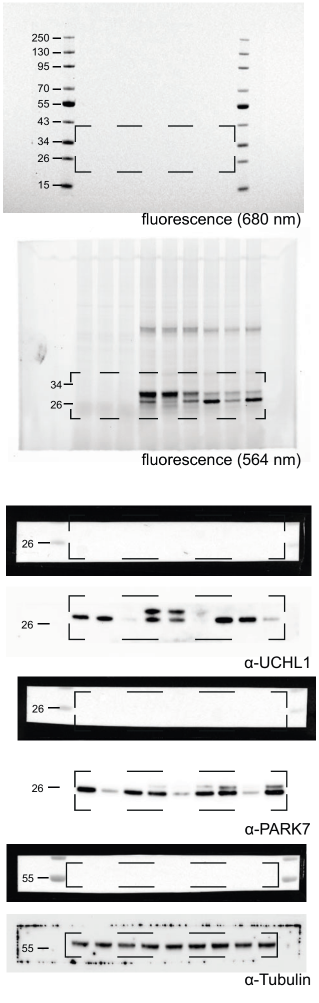

Fig. 2g

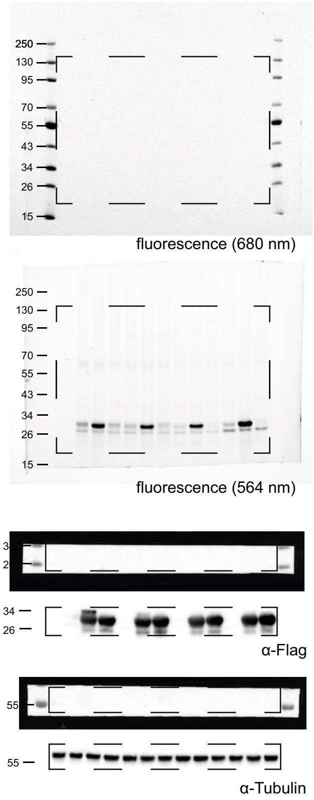

Fig. 2h

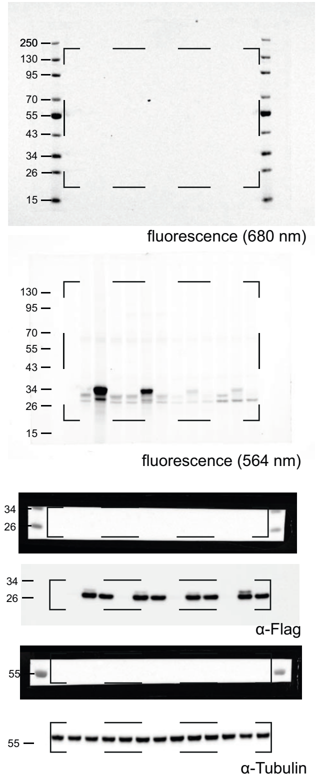

Fig. 3e

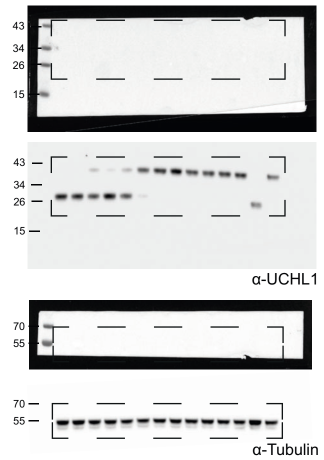

Fig. 4b

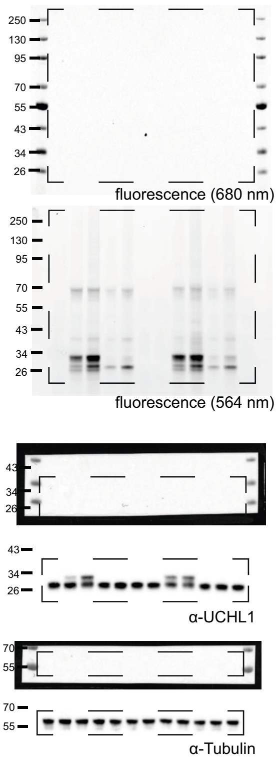

Fig. 4d

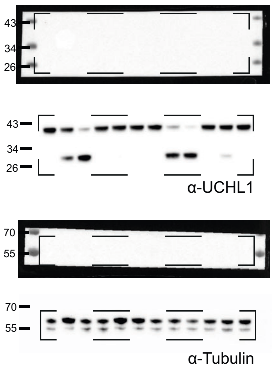

Fig. 4e

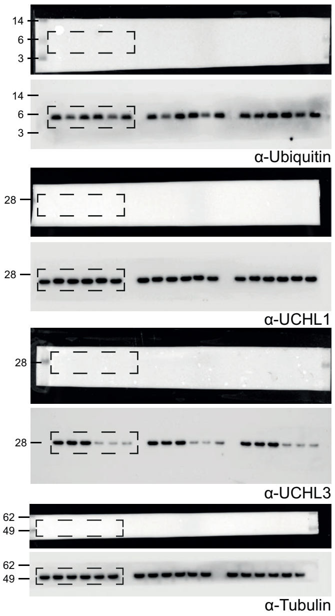

Fig. 5k

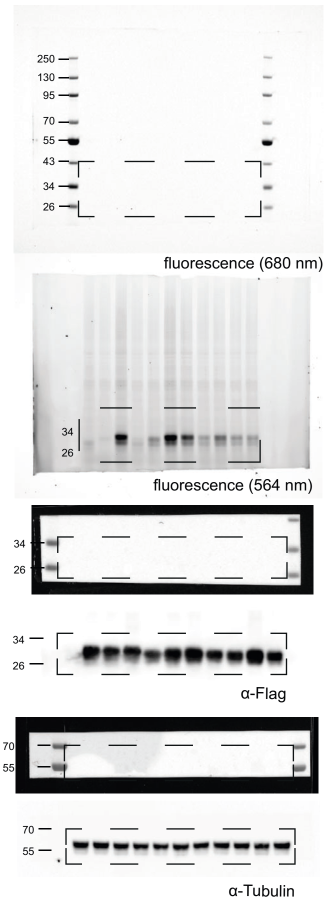
250
130
95
70
55
43
34
26
fluorescence (680 nm)
34
26
fluorescence (564 nm)
34
26
34
26
α-Flag
70
55
70
55
α-Tubulin

Fig. 6e

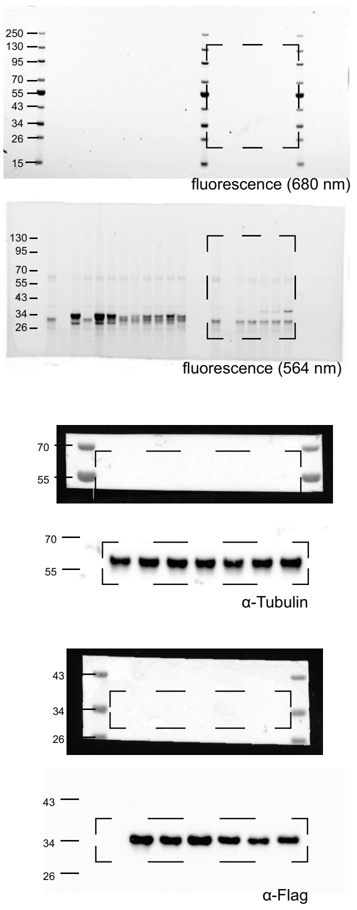
250
130
95
70
55
43
34
26
15
fluorescence (680 nm)
130
95
70
55
43
34
26
fluorescence (564 nm)
70
55
70
55
α-Tubulin
43
34
26
43
34
26
α-Flag

Supplementary Fig. 2a

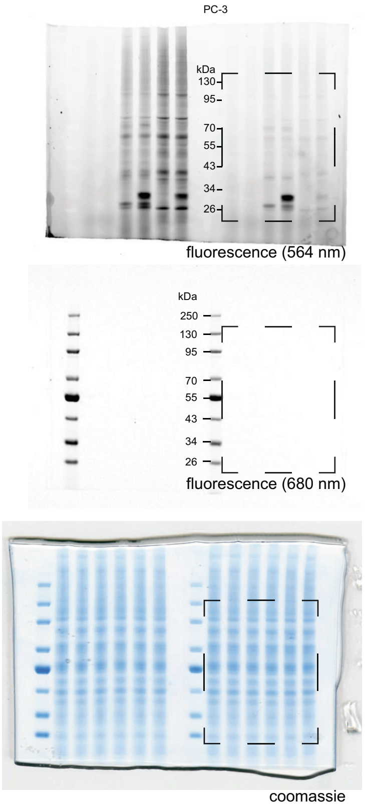

Supplementary Fig. 2b

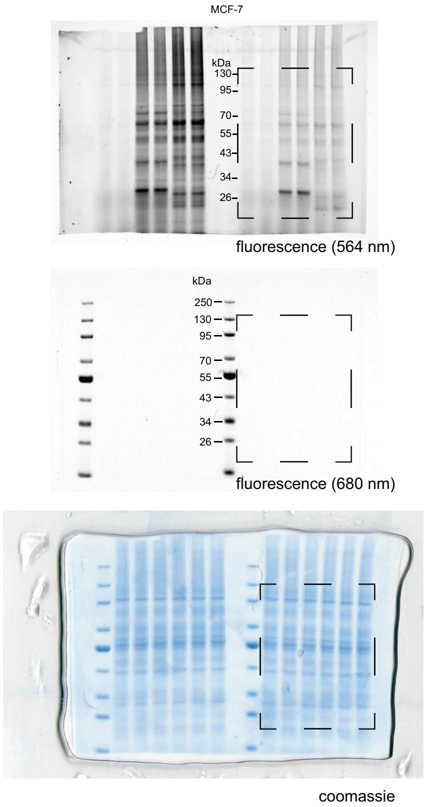

Supplementary Fig. 2c

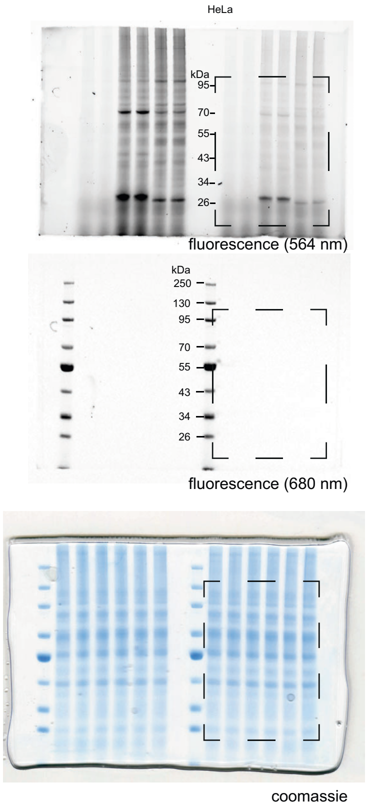

Supplementary Fig. 2e

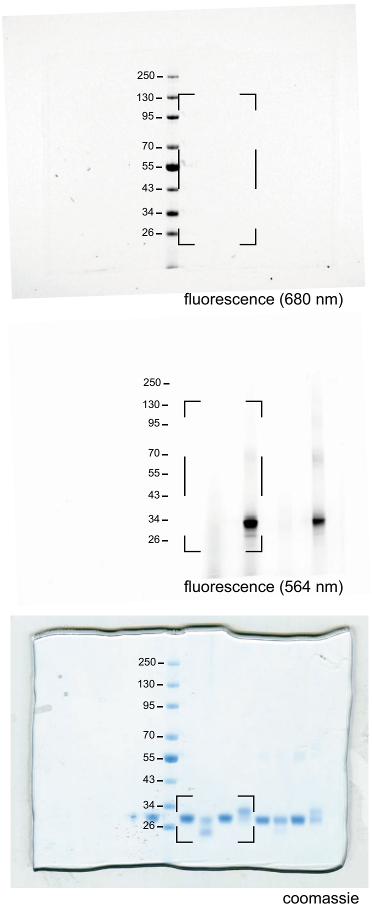

Supplementary Fig. 5f

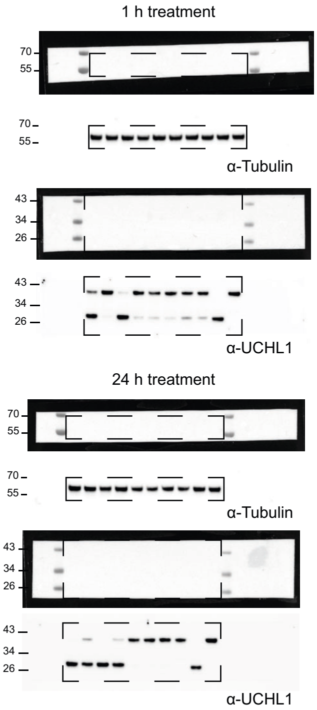

Supplementary Fig. 7f

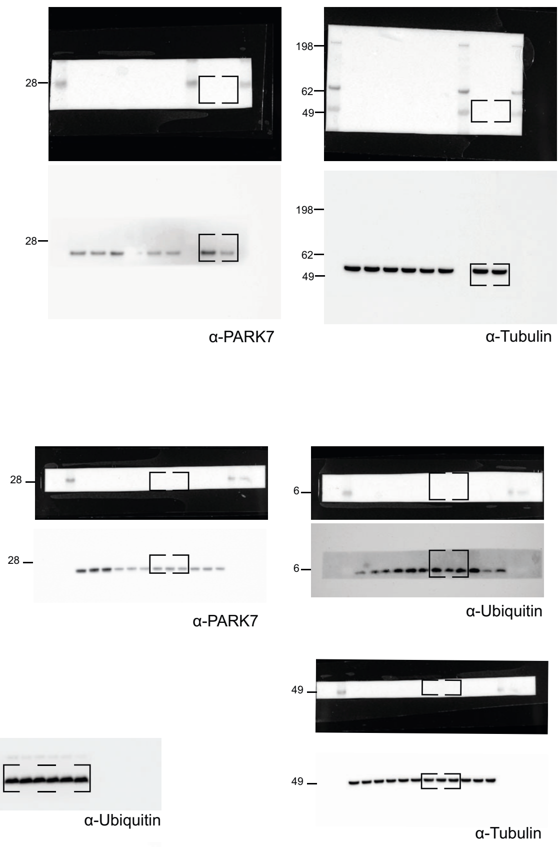

Supplementary Fig. 7e

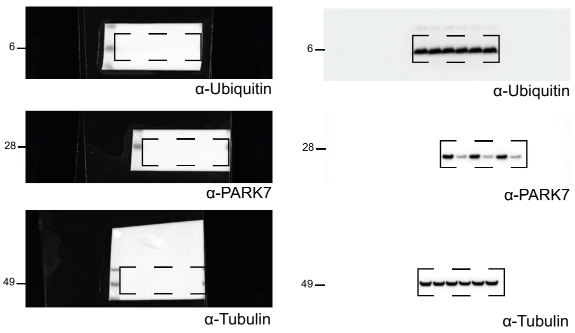

3. Chemical synthesis

Chemicals and solvents were used without further purification, and purchased from companies such as Acros, Activate Scientific, Alfa Aesar, Fluorchem, Merck, Novabiochem, Roth, Sigma-Aldrich, TCI and VWR. LDN-57444 was purchased from Sigma-Aldrich. Solvents used for all synthetic steps were named as follows. EA: ethyl acetate, DCM: dichloromethane, MeOH: methanol, PE: petroleum ether, CH: cyclohexane, DMF: dimethylformamide, THF: tetrahydrofuran, ACN: acetonitrile, H_2O: water, EtOH: ethanol. Thin-layer chromatography was carried out using silica gel aluminum plates (silica gel 60 F254, Merck). The detection was carried out using UV light (λ = 254/366 nm) and potassium permanganate or ninhydrin solution as staining reagents. For purification via column chromatography, silica gel 60 (60Å, 0.035-0.070 mm, Sigma Aldrich) was used. Automated column chromatographic purification was carried out on a Pure C-850 FlashPrep system or a Pure-C-810 Flash system (Büchi). For preparative HPLC, an 1260/1290 Infinity II series system (Agilent Technologies) with a VP125/21 Nucleodur C18 Gravity column (5 µm, Macherey Nagel) was used. Gradient A (40-60% ACN in H_2O + 0.1% TFA over 30 min) or gradient B (20-60% ACN in H_2O + 0.1% TFA over 60 min) was used to purify molecules before biological testing. For chiral HPLC, a Finnigan LTQ Orbitrap XL MS system (Thermo Scientific) with a Lux Cellulose-2 column (5 µm, Phenomenex), an Agilent 1200 Series G1312 binary pump, an Agilent G1315 DAD UV detector, and an Agilent 1200 Series G1367 Hip-ALS autosampler were used. Solvent A: Hexane + 0.1% TFA, solvent B: Isopropanol:MeOH (1:1) + 0.1% TFA; Flowrate: 0.5 ml/ min; Gradient:

Time (min)	Composition
0	A = 88%, B = 12%
16	A = 88%, B = 12%
49	A = 20%, B = 80%
58	A = 20%, B = 80%

For low resolution LC-MS analysis, a 1200 series HPLC system (Agilent Technologies) with a ZORBAX Eclipse XDB column (C18 80 Å; 4.6 x 150 mm; 5 µm) was used. High resolution mass spectrometry (HRMS) was carried out with an LTQ Orbitrap (Thermo Fisher). NMR spectra were recorded on the following devices (all from Bruker): AV 400 Avance III HD (400 MHz for ^1H and 101 MHz for ^{13}C-NMR), AV 500 Avance III HD (500 MHz for ^1H and 125 MHz for ^{13}C-NMR), AV 600 Avance III HD (600 MHz for ^1H and 151 MHz for ^{13}C NMR) and AV 700 Avance III HD (700 MHz for ^1H and 176 MHz for ^{13}C NMR). The chemical shifts of all spectra are specified in ppm. The coupling constants *J* are given in Hertz (Hz). Peaks are referenced to used deuterated solvent (DMSO-d_6: δ = 2.50 ppm / 39.52 ppm; $CDCl_3$: δ = 7.26 ppm / 77.16 ppm; MeOH-d_4: δ = 4.87 ppm / 49.15 ppm). The multiplicities of the signals in the ^1H spectra are abbreviated as follows. s (singlet), d (doublet), dd (doublet of doublets), t (triplet), td (triplet of doublets), q (quartet), m (multiplet) and b (broad).

3.1 Synthesis of CG017

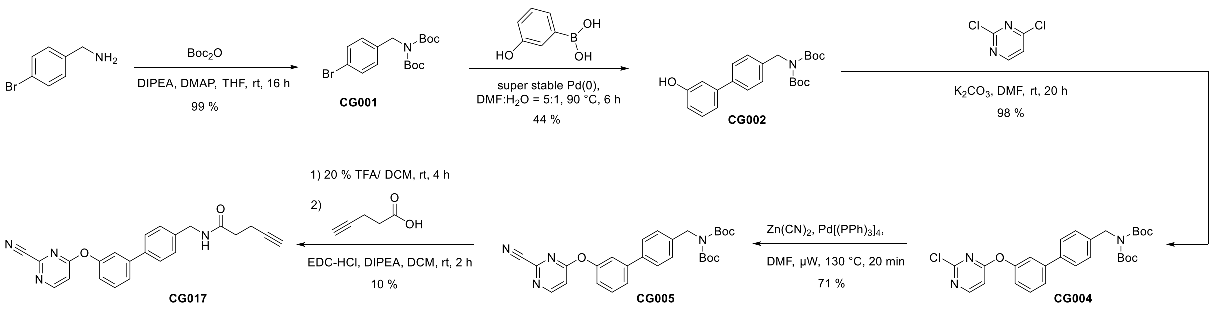

Scheme 3.1: Synthesis of compound CG017 based on [22].

tert-Butyl (4-bromobenzyl)(*tert*-butoxycarbonyl)carbamate (CG001)

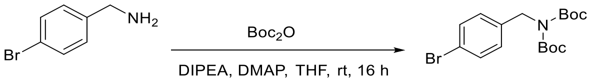

Diisopropylethylamine (DIPEA) (2.08 mL, 12.25 mmol, 1.2 eq) and dimethylaminopyridine (DMAP) (0.62 g, 5.1 mmol, 0.5 eq) were added to a suspension of 4-bromobenzylamine (2.0 g, 10.2 mmol, 1.0 eq) in THF (15 mL) at 0 °C under argon atmosphere. After stirring for 10 min, a solution of Boc-anhydride (Boc_2O) (5.6 g, 25.5 mmol, 2.5 eq) in THF (10 mL) was added dropwise via a cannula. The reaction mixture was stirred at room temperature for 16 h. The reaction mixture was poured into concentrated NH_4Cl (200 mL) and extracted with EA (200 mL). The organic layer was washed with sat. $NaHCO_3$ (100 mL) and brine (100 mL), dried over anhydrous Na_2SO_4 and filtered. The solvent was removed under reduced pressure and the crude product purified via a normal phase silica column. The product was eluting at 10-15% EA in PE and CG001 (3.9 g, 10.1 mmol, 99%) was obtained as a white solid. The analytical characterization was in good agreement with previously reported data[23].

^{1}H NMR (400 MHz, $CDCl_3$) δ (ppm) = 7.43 (d, *J* = 8.3 Hz, 2H), 7.17 (dt, *J* = 8.1, 0.5 Hz, 2H), 4.72 (s, 2H), 1.46 (s, 18H).

^{13}C NMR (126 MHz, DMSO-*d*$_6$) δ (ppm) = 152.16, 137.85, 131.41, 129.24, 120.24, 82.38, 48.41, 27.64.

tert-Butyl (*tert*-butoxycarbonyl)((3'-hydroxy-[1,1'-biphenyl]-4-yl)methyl)-carbamate (CG002)

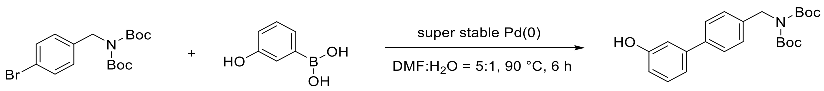

A suspension of CG001 (2.97 g, 7.7 mmol, 1.0 eq), 3-hydroxyphenylboronic acid (1.38 g, 10 mmol, 1.3 eq), tetrakis[tris[3,5-bis(trifluoromethyl)phenyl]phosphine]palladium(0) (super stable Pd(0)) (81.4 mg, 38.4 µmol, 0.005 eq) and K_2CO_3 (2.13 g, 15.4 mmol, 2.0 eq) in a mixture of DMF:H_2O = 5:1 (12 mL) was stirred for 6 h at 90 °C. The reaction mixture was filtered through Celite545 (Roth), poured into H_2O (100 mL) and extracted with EA (100 mL). The organic layer was washed with brine (100 mL), dried over anhydrous Na_2SO_4 and filtered. The organic layer was evaporated under reduced pressure and the crude product was purified on a normal phase silica column. The product was eluting at 15% EA in PE to obtain CG002 (1.3 g, 3.4 mmol, 44%) as a white solid.

^{1}H NMR (500 MHz, DMSO-*d*$_6$) δ (ppm) = 9.52 (s, 1H), 7.57 (d, *J* = 8.3 Hz, 2H), 7.32-7.27 (m, 2H), 7.24 (t, *J* = 7.9 Hz, 1H), 7.05 (dt, *J* = 7.8, 1.2 Hz, 1H), 7.01 (t, *J* = 2.1 Hz, 1H), 6.75 (ddd, *J* = 8.1, 2.4, 0.9 Hz, 1H), 4.71 (s, 2H), 1.41 (s, 18H).

^{13}C NMR (126 MHz, DMSO-d_6) δ (ppm) = 157.83, 152.23, 141.23, 139.14, 137.46, 129.95, 127.49, 126.58, 117.37, 114.42, 113.38, 82.13, 48.61, 27.59.

HRMS *m/z* for $C_{23}H_{30}NO_5^+$ ($[M+H]^+$) calculated: 400.2119, found: 400.2119.

tert-Butyl (tert-butoxycarbonyl)((3'-((2-Chloropyrimidin-4-yl)oxy)-[1,1'-biphenyl]-4-yl)methyl)carbamate (CG004)

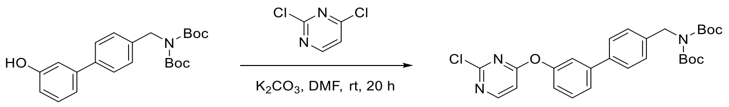

A mixture of CG002 (1.27 g, 3.2 mmol, 1.0 eq), 2,4-dichloropyrimidine (0.95 g, 6.4 mmol, 2.0 eq) and K_2CO_3 (0.88 g, 6.4 mmol, 2.0 eq) in DMF was stirred at room temperature for 20 h. This mixture was quenched with brine (100 mL) and extracted twice with EA (100 mL). The combined organic phases were dried over anhydrous Na_2SO_4, filtered and the solvent was evaporated. The resulting crude product was purified via a normal phase silica column. The product eluted at 20% EA in PE to afford pure CG004 (1.60 g, 3.13 mmol, 98%).

^{1}**H NMR** (500 MHz, DMSO-d_6) δ (ppm) = 8.63 (dd, *J* = 5.6, 0.9 Hz, 1H), 7.71 (d, *J* = 8.1 Hz, 2H), 7.66 (dt, *J* = 7.8, 1.4 Hz, 1H), 7.60 (s, 1H), 7.57 (t, *J* = 7.9 Hz, 1H), 7.32 (d, *J* = 8.1 Hz, 2H), 7.27 (ddd, *J* = 8.0, 2.3, 1.1 Hz, 1H), 7.20 (dd, *J* = 5.6, 1.0 Hz, 1H), 4.72 (s, 2H), 1.41 (s, 18H).

^{13}**C NMR** (126 MHz, DMSO-d_6) δ (ppm) = 170.17, 162.32, 161.53, 159.02, 152.20, 141.76, 138.15, 137.60, 130.61, 127.56, 126.79, 124.36, 120.47, 119.49, 107.70, 82.16, 48.58, 27.58.

HRMS *m/z* for $C_{27}H_{31}ClN_3O_5^+$ ($[M+H]^+$) calculated: 512.1947, found: 512.1949.

tert-Butyl (tert-butoxycarbonyl)((3'-((2-cyanopyrimidin-4-yl)oxy)-[1,1'-biphenyl]-4-yl)methyl)carbamate (CG005)

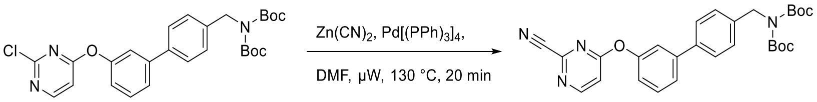

A mixture of CG004 (50 mg, 0.10 mmol, 1.0 eq), $Zn(CN)_2$ (11.5 mg, 0.10 mmol, 1.0 eq) and $Pd(PPh_3)_4$ (11.3 mg, 0.01 mmol, 0.1 eq) in DMF (1 mL) was irradiated in a microwave reactor at 130 °C for 20 min. The reaction was quenched with sat. $NaHCO_3$ (20 mL) and extracted with EA (30 mL). The organic layer was washed with brine (20 mL), dried over anhydrous Na_2SO_4 and the solvent was evaporated under reduced pressure. The product was purified using a silica column, eluting at 30% EA in PE to yield pure CG005 (149 mg, 0.30 mmol, 71%).

^{1}**H NMR** (600 MHz, DMSO-d_6) δ (ppm) = 8.86 (d, *J* = 5.9 Hz, 1H), 7.71 (d, *J* = 8.2 Hz, 2H), 7.67 (dt, *J* = 8.0, 1.2 Hz, 1H), 7.61 (d, *J* = 2.1 Hz, 1H), 7.59 (t, *J* = 7.9 Hz, 1H), 7.52 (d, *J* = 5.9 Hz, 1H), 7.32 (d, *J* = 8.0 Hz, 2H), 7.28 (dd, *J* = 8.0, 2.0 Hz, 1H), 4.72 (s, 2H), 1.41 (s, 18H).

^{13}**C NMR** (151 MHz, DMSO-d_6) δ (ppm) = 170.15, 161.50, 159.02, 152.26, 152.19, 141.76, 138.15, 137.60, 130.59, 127.55, 126.76, 124.33, 120.42, 119.44, 107.66, 82.16, 48.58, 27.60, 27.57.

HRMS *m/z* for $C_{28}H_{31}N_4O_5^+$ ($[M+H]^+$) calculated: 503.2289, found: 503.2289.

***N*-((3'-((2-Cyanopyrimidin-4-yl)oxy)-[1,1'-biphenyl]-4-yl)methyl)pent-4-ynamide (CG017)**

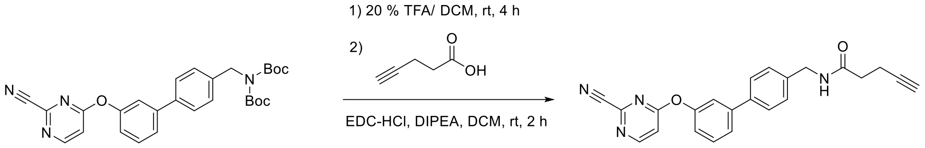

Step 1

CG005 (50 mg, 0.10 mmol, 1.0 eq) was dissolved in DCM (2 mL) and TFA (400 µL) was added dropwise to the solution. After completion of the deprotection, the reaction mixture was poured into sat. $NaHCO_3$ (50 mL) and extracted with EA (50 mL). The organic layer was washed with brine (50 mL), dried over anhydrous Na_2SO_4, filtered and the solvent evaporated under reduced pressure.

Step 2

4-Pentynoic acid (12.3 mg, 0.12 mmol, 1.2 eq), EDC-HCl (28.6 mg, 0.15 mmol, 1.5 eq) and DIPEA (25.4 µL, 0.15 mmol, 1.5 eq) were dissolved in DCM (1 mL) and stirred for 10 min at room temperature. To this reaction was added the crude product from step 1 in DCM (1 mL) dropwise. The reaction was stirred for 2 h until full conversion of the educt was observed by TLC. The reaction was quenched with sat. $NaHCO_3$ (30 mL) and extracted with EA (40 mL). The organic layer was washed with brine (30 mL), dried over anhydrous Na_2SO_4, filtered and the solvent evaporated under reduced pressure. The product was purified using a preparative HPLC to yield pure CG017 (4 mg, 0.01 mmol, 10%).

^{1}H NMR (600 MHz, $CDCl_3$) δ (ppm) = 8.79 (d, *J* = 4.8 Hz, 1H), 7.56 (d, *J* = 8.2 Hz, 2H), 7.52 (d, *J* = 5.1 Hz, 2H), 7.39 (s, 2H), 7.37 (d, *J* = 4.7 Hz, 2H), 7.19 – 7.15 (m, 1H), 5.99 (s, 1H), 4.52 (d, *J* = 5.7 Hz, 2H), 2.59 (td, *J* = 7.1, 2.7 Hz, 2H), 2.48 (t, *J* = 7.1 Hz, 2H), 2.01 (t, *J* = 2.6 Hz, 1H).

^{13}C NMR (151 MHz, $CDCl_3$) δ (ppm) = 171.32, 165.62, 162.15, 152.69, 143.42, 142.87, 139.54, 137.73, 130.31, 128.52, 127.64, 124.99, 120.42, 120.20, 119.29, 114.99, 82.97, 69.79, 43.59, 35.53, 15.12.

HRMS *m/z* for $C_{23}H_{19}N_4O_2^+$ ($[M+H]^+$) calculated: 383.1503, found: 383.1500.

3.2 Synthesis of CG041

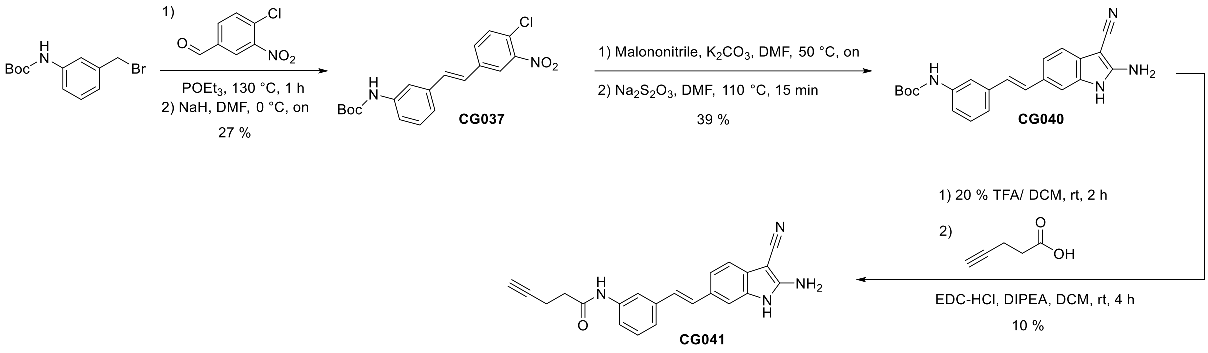

Scheme 3.2: Synthesis of compound CG041 based on [24].

tert-Butyl (*E*)-(3-(4-chloro-3-nitrostyryl)phenyl)carbamate (CG037)

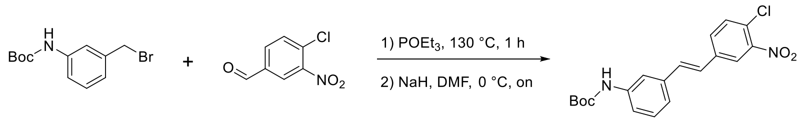

Step 1

tert-Butyl (3-(bromomethyl)phenyl)carbamate (463 mg, 1.6 mmol, 1.0 eq) and $POEt_3$ (448 µL, 1.6 mmol, 2.6 eq) were heated to 130 °C for 1 h until the formation of *tert*-butyl (3-((diethoxyphosphoryl)methyl)phenyl)carbamate was finished, as judged by LC-MS and TLC. Residual $POEt_3$ was removed under reduced pressure and the product was used for step 2 without further purification.

Step 2

The crude product from step 1 was diluted with DMF (3 mL) and cooled to 0 °C. NaH (129 mg, 3.2 mmol, 2.0 eq) was added portionwise to the solution at 0 °C. After stirring for 20 min, a solution of 4-chloro-3-nitrobenzaldehyde (300.0 mg, 1.6 mmol, 1.0 eq) in DMF (2 mL) was added dropwise at 0 °C. After 12 h the mixture was diluted with EA (50 mL) and the organic layer was washed with H_2O (30 mL) and brine (50 mL). After drying over anhydrous $MgSO_4$, the organic layer was filtered and concentrated under reduced pressure. The compound was purified via a silica column, eluting at 5% EA in PE, yielding CG037 (165 mg, 0.4 mmol, 27%) as a red solid.

^{1}H NMR (500 MHz, $CDCl_3$) δ (ppm) = 7.97 (d, *J* = 2.1 Hz, 1H), 7.74 (d, *J* = 4.7 Hz, 1H), 7.60 (dd, *J* = 8.4, 2.1 Hz, 1H), 7.51 (d, *J* = 8.4 Hz, 1H), 7.29 (t, *J* = 7.8 Hz, 1H), 7.20-7.15 (m, 2H), 7.13 (d, *J* = 16.4 Hz, 1H), 7.04 (d, *J* = 16.3 Hz, 1H), 6.55 (s, 1H), 1.54 (s, 9H).

^{13}C NMR (126 MHz, $CDCl_3$) δ (ppm) = 152.83, 148.31, 139.08, 137.66, 137.05, 132.19, 130.71, 129.54, 125.48, 125.39, 123.14, 121.88, 118.77, 116.56, 80.94, 28.48.

HRMS *m/z* for $C_{19}H_{20}ClN_2O_4^+$ ($[M+H]^+$) calculated: 375.1112, found: 375.1104.

***tert*-Butyl (*E*)-(3-(2-(2-amino-3-cyano-1*H*-indol-6-yl)vinyl)phenyl)carbamate (CG040)**

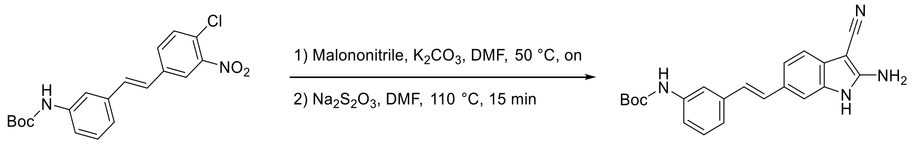

A stirred solution of CG037 (150 mg, 0.4 mmol, 1.0 eq) and malononitrile (66.1 mg, 1.0 mmol, 2.5 eq) in DMF (5 mL) was treated with K_2CO_3 (221 mg, 1.6 mmol, 4.0 eq). The mixture was stirred at 50 °C overnight. To this solution was added DMF (2 mL), H_2O (2 mL) and $Na_2S_2O_3$ (697 mg, 4.0 mmol, 10.0 eq) and the mixture heated to 110 °C for 15 min. The solution was poured into H_2O (50 mL) and extracted with EA (100 mL). The organic layer was washed with brine (100 mL), dried over anhydrous $MgSO_4$ and filtered. The solvent was evaporated, and the product purified on a reverse phase C18-column, eluting at 52% ACN in H_2O, yielding CG040 (58.8 mg, 0.16 mmol, 39%).

^{1}H NMR (500 MHz, DMSO-*d*6) δ (ppm) = 10.75 (s, 1H), 9.34 (s, 1H), 7.66 (d, *J* = 2.0 Hz, 1H), 7.34 (d, *J* = 1.5 Hz, 1H), 7.26 (dd, *J* = 8.2, 1.6 Hz, 2H), 7.22 (t, *J* = 7.6 Hz, 1fH), 7.20-7.16 (m, 1H), 7.12 (d, *J* = 16.1 Hz, 1H), 7.10 (s, 1H), 6.99 (d, *J* = 16.4 Hz, 1H), 6.86 (br s, 2H), 1.49 (s, 9H).

^{13}C NMR (126 MHz, DMSO-*d*6) δ (ppm) = 154.55, 152.85, 139.87, 138.00, 132.62, 129.66, 128.90, 128.46, 125.07, 119.96, 118.15, 117.71, 117.03, 115.76, 115.18, 114.72, 108.30, 79.05, 62.12, 28.18.

HRMS *m/z* for $C_{22}H_{23}N_4O_2^+$ ($[M+H]^+$) calculated: 375.1816, found: 375.1815.

(*E*)-*N*-(3-(2-(2-Amino-3-cyano-1*H*-indol-6-yl)vinyl)phenyl)pent-4-ynamide (CG041)

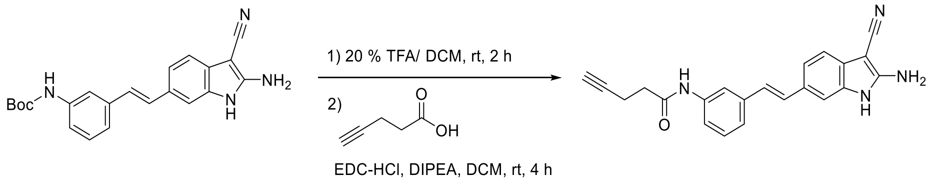

Step 1

CG040 (50 mg, 0.13 mmol, 1.0 eq) was dissolved in DCM (3 mL) and TFA (600 µL) was added dropwise. After the completion of the deprotection as judged by TLC, the reaction mixture was poured into sat. $NaHCO_3$ (50 mL) and extracted with EA (50 mL). The organic layer was washed with brine (50 mL), dried over anhydrous $MgSO_4$, filtered and the solvent evaporated under reduced pressure.

Step 2

The crude product from step 1, EDC-HCl (38.4 mg, 0.20 mmol, 1.5 eq), HOBt (20.4 mg, 0.13 mmol, 1.0 eq), 4-pentynoic acid (16.5 mg, 0.16 mmol, 1.2 eq) and DIPEA (34 µL, 0.20 mmol, 1.5 eq) were dissolved in DMF (2 mL) and stirred for 4 h at room temperature. The reaction was quenched with sat. $NaHCO_3$ (50 mL) and extracted with EA (100 mL). The organic layer was washed with brine (50 mL), dried over anhydrous $MgSO_4$, filtered and the solvent evaporated under reduced pressure. The product was purified using a preparative HPLC eluting at 55% ACN, yielding CG041 (4.7 mg, 0.01 mmol, 10%).

^{1}H NMR (700 MHz, DMSO-*d*6) δ (ppm) = 10.76 (s, 1H), 9.98 (s, 1H), 7.79 (t, *J* = 1.8 Hz, 1H), 7.43 (dt, *J* = 7.2, 2.1 Hz, 1H), 7.34 (d, *J* = 1.5 Hz, 1H), 7.29-7.24 (m, 3H), 7.15 (d, *J* = 16.3 Hz, 1H), 7.11 (d, *J* = 8.0 Hz, 1H), 7.02 (d, *J* = 16.3 Hz, 1H), 6.84 (s, 2H), 2.81 (t, *J* = 2.6 Hz, 1H), 2.54 (t, *J* = 7.0 Hz, 2H), 2.52-2.46 (m, 2H).

^{13}C NMR (176 MHz, DMSO-*d*6) δ (ppm) = 169.38, 154.52, 139.44, 138.06, 132.58, 129.81, 128.97, 128.82, 128.48, 124.86, 121.07, 119.91, 117.85, 117.62, 116.56, 115.15, 108.29, 83.64, 71.49, 62.15, 35.22, 14.07.

HRMS *m/z* for $C_{22}H_{19}N_4O^+$ ($[M+H]^+$) calculated: 355.1553, found: 355.1551.

3.3 Synthesis of 3-carboxypyrrolidine-based probes and controls

3.3.1 Synthesis of GK12S/R and GK13S/R

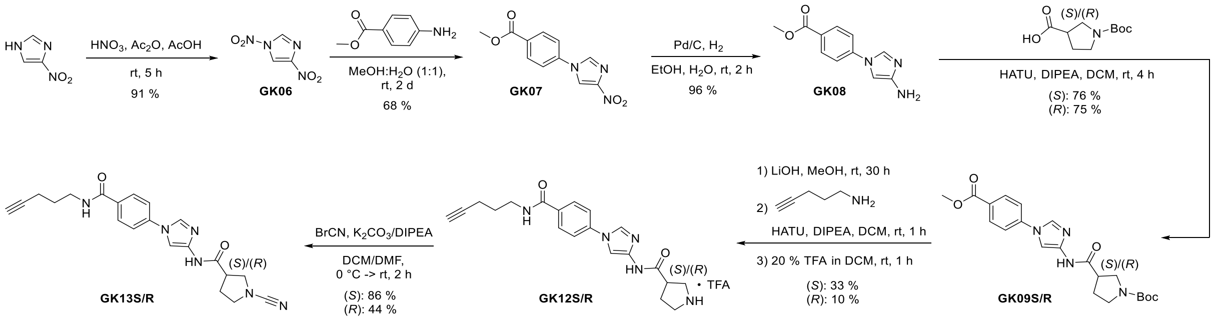

Scheme 3.3.1: Synthesis of compounds GK13S, GK13R, GK12S, and GK12R based on [25].

1,4-Dinitro-1*H*-imidazole (GK06)

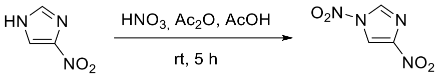

4-Nitroimidazole (2.00 g, 17.7 mmol, 1.0 eq) was dissolved in acetic acid (36 mL) and cooled to 0 °C. Concentrated nitric acid (9 mL) was added dropwise over 30 min. Then acetic anhydride (24 mL) was added dropwise over 30 min. The solution was stirred for 4 h at room temperature. The reaction was slowly poured into sat. K_2CO_3 solution (250 mL) and the aqueous phase was extracted twice with EA (50 mL). The combined organic layers were washed with sat. K_2CO_3 solution (100 mL), brine (100 mL) and dried with $MgSO_4$. The solvent was removed under reduced pressure. The obtained yellow crystals (2.56 g, 16.2 mmol, 91% yield) were used for the next reaction without further purification.

^{1}H NMR (700 MHz, DMSO-*d*$_6$) δ (ppm) = 9.42 (d, *J* = 1.6 Hz, 1H), 8.98 (d, *J* = 1.6 Hz, 1H).

^{13}C NMR (176 MHz, DMSO-*d*$_6$) δ (ppm) = 136.32, 133.14, 116.44.

Methyl 4-(4-nitro-1*H*-imidazol-1yl)benzoate (GK07)

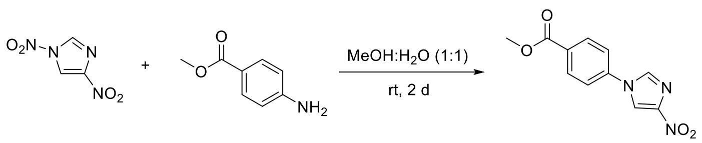

GK06 (2.56 g, 16.18 mmol, 1.0 eq) was dissolved in a 1:1 mixture of MeOH and H_2O (120 mL). Methyl 4-aminobenzoate (641 mg, 4.24 mmol, 1.0 eq) was added and the reaction mixture was stirred for two days at room temperature in darkness. The obtained red suspension was cooled down to 0 °C and the precipitate was filtered and washed with cold MeOH in H_2O (1:1). The red solid was recrystallized in a mixture of MeOH, H_2O and concentrated hydrochloric acid (2000:100:1) (100 mL) at 90 °C. The product was obtained as a yellow solid (2.74 g, 11.1 mmol, 68% yield). The analytical characterization was in good agreement with previously reported data.[26]

^{1}H NMR (700 MHz, DMSO-*d*$_6$) δ (ppm) = 9.10 (d, *J* = 1.6 Hz, 1H), 8.60 (d, *J* = 1.5 Hz, 1H), 8.11 (d, *J* = 8.8 Hz, 2H), 7.99 (d, *J* = 8.8 Hz, 2H), 3.89 (s, 3H).

^{13}C NMR (176 MHz, DMSO-*d*$_6$) δ (ppm) = 165.26, 148.33, 138.95, 135.71, 130.84, 129.32, 121.23, 119.56, 52.44.

HRMS *m/z* for $C_{11}H_{10}N_3O_4^+$ ($[M+H]^+$) calculated: 248.0666, found: 248.0666.

Methyl 4-(4-amino-1*H*-imidazol-1yl)benzoate (GK08)

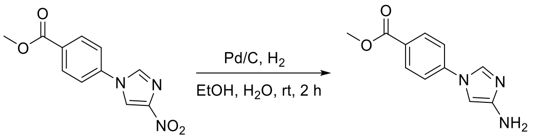

GK07 (500 mg, 2.02 mmol, 1.0 eq) was dissolved in EtOH (25 mL) and a few drops of H_2O were added. Palladium (10%) on activated charcoal (100 mg) was added under argon atmosphere. The reaction was flushed with H_2 and stirred for 2 h at room temperature. The reaction was filtered through celite545 which was then washed with EA. The solvent was dried over anhydrous $MgSO_4$ and removed under reduced pressure. The obtained yellow solid (423 mg, 1.94 mmol, 96%) was used for the next reaction without further purification. The analytical characterization was in good agreement with previously reported data.[26]

^{1}H NMR (400 MHz, DMSO-*d6*) δ (ppm) = 8.03 (d, *J* = 1.6 Hz, 1H), 8.00 (d, *J* = 8.9 Hz, 2H), 7.69 (d, *J* = 8.9 Hz, 2H), 6.73 (d, *J* = 1.7 Hz, 1H), 3.32 (s, 3H).

^{13}C NMR (126 MHz, DMSO-*d6*) δ (ppm) = 165.59, 149.07, 140.75, 131.82, 130.96, 126.27, 118.43, 95.11, 52.21.

HRMS *m/z* for $C_{11}H_{12}N_3O_2^+$ ($[M+H]^+$) calculated: 218.0924, found: 218.0924.

***tert*-Butyl-(*S*)-3-((1-(4-(Methoxycarbonyl)phenyl)-1*H*-Imidazol-4-yl)carbamoyl)-pyrrolidine-1-carboxylate (GK09S)**

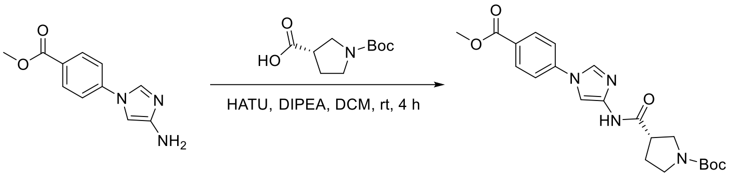

(*S*)-1-(*tert*-Butoxycarbonyl)pyrrolidine-3-carboxylic acid (198 mg, 0.92 mmol, 1.0 eq) was dissolved in DCM (5 mL), to which DIPEA (313 µL, 1.84 mmol, 2.0 eq) and HATU (525 mg, 1.38 mmol, 1.5 eq) were added. The solution was stirred for 30 min at room temperature. GK08 (200 mg, 0.60 mmol, 1.0 eq) dissolved in DCM (6 mL) was added dropwise to the reaction mixture. After additional 30 min the reaction mixture was quenched with sat. NH_4Cl-solution (20 ml) and extracted with DCM (40 mL). The organic phase was washed with brine (20 mL), dried over anhydrous $MgSO_4$ and the solvent was removed under reduced pressure. The compound was purified via a silica column eluting at 60% EA in PE, yielding GK09S (320 mg, 0.77 mmol, 76%) as a brown solid.

^{1}H NMR (500 MHz, $CDCl_3$) δ (ppm) = 9.89 (d, *J* = 110.6 Hz, 1H), 8.17 (d, *J* = 8.6 Hz, 2H), 7.93 (d, *J* = 12.8 Hz, 1H), 7.86 (s, 1H), 7.52 (d, *J* = 8.7 Hz, 2H), 3.95 (s, 3H), 3.85-3.65 (m, 1H), 3.65-3.53 (m, 2H), 3.43-3.32 (m, 1H), 3.21-3.09 (m, 1H), 2.33-2.15 (m, 2H), 1.45 (s, 9H).

^{13}C NMR (126 MHz, $CDCl_3$) δ (ppm) = 169.73, 165.50, 153.38, 140.36, 139.49, 132.30, 131.04, 127.37, 119.53, 103.90, 78.35, 54.96, 52.31, 48.48, 45.28, 43.14, 42.26, 28.20

HRMS *m/z* for $C_{21}H_{26}N_4O_5^+$ ($[M+H]^+$) calculated: 415.1976, found: 415.1976.

tert-Butyl-(R)-3-((1-(4-(Methoxycarbonyl)phenyl)-1H-Imidazol-4-yl)carbamoyl)-pyrrolidine-1-carboxylate (GK09R)

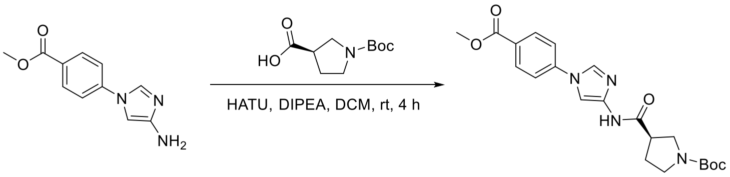

GK09R (313 mg, 0.76 mmol, 75%) was obtained as a brown solid from (*R*)-1-(*tert*-Butoxycarbonyl)pyrrolidine-3-carboxylic acid as described above for GK09S.

^{1}H NMR (500 MHz, $CDCl_3$) δ (ppm) = 9.97 (d, *J* = 103.4 Hz, 1H), 8.16 (d, *J* = 8.6 Hz, 2H), 7.89 (d, *J* = 12.9 Hz, 1H), 7.86 (s, 1H), 7.52 (d, *J* = 8.7 Hz, 2H), 3.94 (s, 3H), 3.86-3.67 (m, 1H), 3.63-3.54 (m, 2H), 3.44-3.33 (m, 1H), 3.23-3.09 (m, 1H), 2.35-2.15 (m, 2H), 1.45 (s, 9H).

^{13}C NMR (126 MHz, $CDCl_3$) δ (ppm) = 169.94, 166.00, 154.54, 140.19, 138.39, 131.76, 130.64, 129.56, 120.54, 106.14, 79.70, 52.58, 48.75, 45.65, 44.50, 28.62.

HRMS *m/z* for $C_{21}H_{26}N_4O_5^+$ ($[M+H]^+$) calculated: 415.1976, found: 415.1976.

(S)-N-(1-(4-(Pent-4-yn-1-ylcarbamoyl)phenyl)-1H-imidazol-4-yl)pyrrolidine-3-carboxamide TFA salt (GK12S)

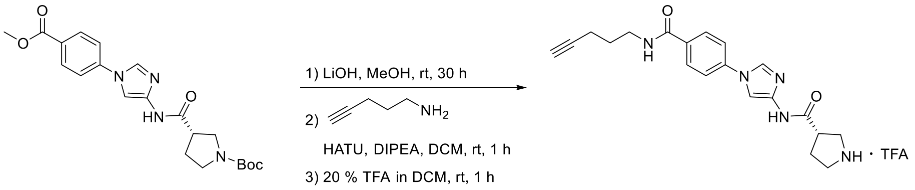

Step 1

GK09S (290 mg, 0.70 mmol, 1.0 eq) was dissolved in MeOH (7.5 mL). Aqueous 2 M LiOH solution (875 µL) was added and the reaction mixture was stirred for 30 h at room temperature. To the reaction mixture was added 1 M hydrochloric acid (700 µL) to neutralize the excess of LiOH. The solvent was removed under reduced pressure and the obtained crude product was used for the next reaction without further purification.

Step 2

The crude product from step 1 was dissolved in DMF (4 mL) and DCM (3 mL). DIPEA (285 µL, 1.67 mmol, 2.0 eq) and HATU (636 mg, 1.67 mmol, 2.0 eq) were added and the reaction mixture was stirred for 30 min. 4-Pentyn-1-amine hydrochloride (100 mg, 0.84 mmol, 1.0 eq) was dissolved in DCM (2 mL) and added to the reaction dropwise. After 30 min the reaction mixture was quenched with sat. NH_4Cl solution (20 ml) and extracted with DCM (40 mL). The organic phase was washed with brine (20 mL), dried over $MgSO_4$ and the solvent was removed under reduced pressure. The obtained crude product was used for the next reaction without further purification.

Step 3

The crude product from step 2 was dissolved in DCM (4 mL) and TFA (1 mL) was added dropwise. The solution was stirred for 1 h at room temperature. The solvent was removed under reduced pressure. The product was purified by preparative HPLC eluting at 10-20% ACN in H_2O, yielding GK12S as a TFA salt (109 mg, 0.23 mmol, 33% yield).

^{1}H NMR (500 MHz, DMSO-d_6) δ (ppm) = 10.89 (s, 1H), 9.05 (s, 2H), 8.61 (t, *J* = 5.6 Hz, 1H), 8.28 (d, *J* = 1.7 Hz, 1H), 7.98 (d, *J* = 8.7 Hz, 2H), 7.79 (d, *J* = 1.7 Hz, 1H), 7.74 (d, *J* = 8.7 Hz, 2H), 3.48-3.39 (m,

1H), 3.34 (m, 4H), 3.29-3.17 (m, 2H), 2.80 (t, *J* = 2.6 Hz, 1H), 2.29-2.18 (m, 3H), 2.08-1.98 (m, 1H), 1.71 (p, *J* = 7.1 Hz, 2H).

^{13}C NMR (126 MHz, DMSO-*d$_6$*) δ (ppm) = 169.01, 165.30, 158.52 (q, *J* = 34.5 Hz), 139.01, 138.73, 132.56, 132.31, 129.09, 119.40, 116.25 (q, *J* = 293.8 Hz), 104.39, 84.21, 71.49, 47.04, 45.12, 42.03, 38.57, 29.02, 28.12, 15.61.

HRMS *m/z* for $C_{20}H_{24}N_5O_2^+$ ($[M+H]^+$) calculated: 366.1924, found: 366.1924.

(*R*)-*N*-(1-(4-(Pent-4-yn-1-ylcarbamoyl)phenyl)-1*H*-imidazol-4-yl)pyrrolidine-3-carboxamide (GK12R)

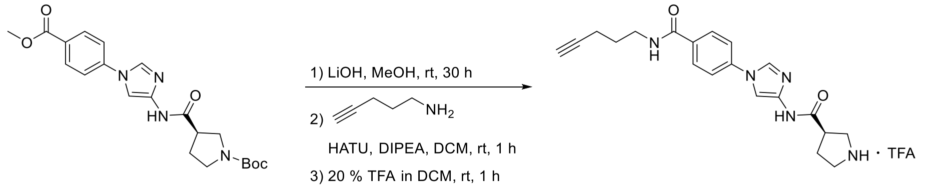

GK12R was obtained from GK09R as described above for GK12S. For step 3, the crude product was purified by column chromatography using a reverse phase silica column. The product was eluting at 15-25% ACN (+0.1% TFA) in H_2O (+0.1% TFA). Parts of the product were further purified by preparative HPLC, eluting at 10-20% ACN, yielding GK12R as a TFA salt (39 mg, 0.08 mmol, 10%).

^{1}H NMR (500 MHz, DMSO-*d$_6$*) δ (ppm) = 10.88 (s, 1H), 9.03 (s, 2H), 8.60 (t, *J* = 5.6 Hz, 1H), 8.28 (d, *J* = 1.7 Hz, 1H), 8.00-7.96 (m, 2H), 7.79 (d, *J* = 1.6 Hz, 1H), 7.76-7.72 (m, 2H), 3.50-3.38 (m, 1H), 3.37-3.30 (m, 4H), 3.28-3.17 (m, 2H), 2.81 (t, *J* = 2.6 Hz, 1H), 2.23 (td, *J* = 7.1, 2.8 Hz, 3H), 2.03 (dq, *J* = 12.8, 7.3 Hz, 1H), 1.72 (p, *J* = 7.1 Hz, 2H).

^{13}C NMR (126 MHz, DMSO-*d$_6$*) δ (ppm) = 168.95, 165.24, 158.38 (q, *J* = 33.7 Hz), 139.08, 138.71, 132.51, 132.30, 129.05, 119.35, 116.50 (d, *J* = 295.3 Hz), 104.30, 84.18, 71.49, 47.02, 45.09, 42.00, 38.54, 28.99, 28.09, 15.58.

HRMS *m/z* for $C_{20}H_{24}N_5O_2^+$ ($[M+H]^+$) calculated: 366.1925, found: 366.1924.

(*S*)-1-Cyano-*N*-(1-(4-(pent-4-yn-1ylcarbamoyl)phenyl)-1*H*-imidazol-4-yl)pyrrolidine-3-carboxamide (GK13S).

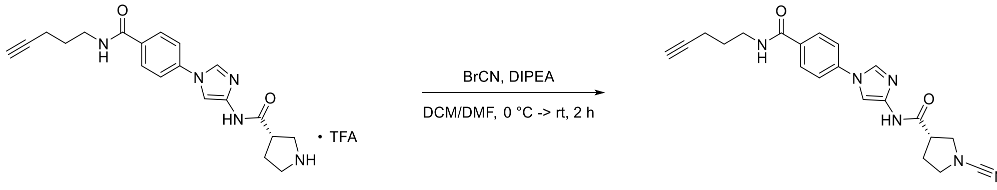

GK12S (70 mg, 0.15 mmol, 1.0 eq) was dissolved in DCM (3 mL) and DMF (1 mL), then DIPEA (127 µL, 0.73 mmol, 5.0 eq) was added. The mixture was cooled to 0 °C and stirred for 30 min. Then 3 M cyanogen bromide solution (58 µL, 0.18 mmol, 1.2 eq) was added and the mixture was stirred for 30 min at 0 °C. The reaction mixture was allowed to warm up to room temperature and stirred for another 2 h followed by the addition of H_2O (10 mL). The two phases were separated, and the aqueous phase was extracted two times with EA (20 mL). The combined organic layers were dried over anhydrous $MgSO_4$, filtered and the solvent was removed under reduced pressure. The corresponding product was purified via a silica column eluting at 4% MeOH in DCM, yielding GK13S (49 mg, 0.13 mmol, 86%) as light brown crystals.

^{1}H NMR (700 MHz, DMSO-d_6) δ (ppm) = 10.74 (s, 1H), 8.56 (t, *J* = 5.6 Hz, 1H), 8.25 (s, 1H), 7.97 (d, *J* = 8.2 Hz, 2H), 7.81 (s, 1H), 7.75 (d, *J* = 8.2 Hz, 2H), 3.60 (t, *J* = 8.6 Hz, 1H), 3.47 (td, *J* = 8.8, 8.3, 5.8 Hz, 2H), 3.40 (q, *J* = 7.7 Hz, 1H), 3.36 – 3.32 (m, 2H), 3.28 – 3.22 (m, 1H), 2.82 – 2.76 (m, 1H), 2.23 (dt, *J* = 7.2, 4.3 Hz, 3H), 2.09 (ddq, *J* = 81.0, 14.1, 7.1 Hz, 2H), 1.72 (p, *J* = 7.2 Hz, 2H).

^{13}C NMR (176 MHz, DMSO-d_6) δ (ppm) = 169.02, 165.19, 139.18, 138.71, 132.37, 132.09, 128.95, 119.20, 117.24, 104.17, 84.12, 71.39, 52.55, 50.03, 43.04, 38.48, 29.54, 28.04, 15.53.

HRMS *m/z* for $C_{21}H_{23}N_6O_2^+$ ([M+H]$^+$) calculated: 391.1877, found: 391.1881.

ee >99%, t_R (min) = 49.83.

(*R*)-1-Cyano-*N*-(1-(4-(pent-4-yn-1ylcarbamoyl)phenyl)-1*H*-imidazol-4-yl)pyrrolidine-3-carboxamide (GK13R)

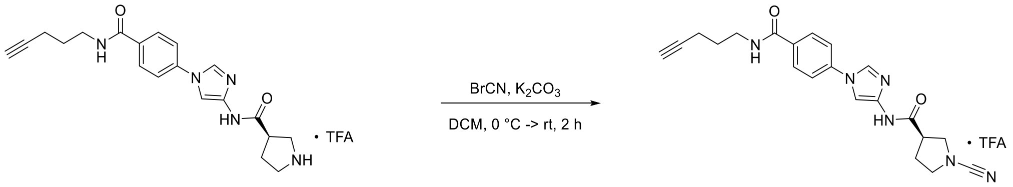

GK12R (53 mg, 0.11 mmol, 1.0 eq) was dissolved in DCM (3 mL) and K_2CO_3 (61 mg, 0.44 mmol, 4.0 eq) was added. The mixture was cooled to 0 °C and stirred for 30 min. Then 3 M cyanogen bromide solution (44 µL, 0.13 mmol, 1.2 eq) was added and the mixture was stirred for 2 h at 0 °C. The reaction mixture was allowed to warm up to room temperature and stirred for another 2 h followed by the addition of H_2O (10 mL). The two phases were separated, and the aqueous phase was extracted two times with EA (20 mL). The combined organic layers were dried over anhydrous $MgSO_4$, filtered and the solvent was removed under reduced pressure. The corresponding product was purified via preparative HPLC eluting at 20-30% ACN (+0.1% TFA) in H_2O (+0.1% TFA) yielding GK13R as a TFA salt (21 mg, 0.05 mmol, 44%).

^{1}H NMR (500 MHz, DMSO-d_6) δ (ppm) = 10.77 (s, 1H), 8.58 (t, *J* = 5.6 Hz, 1H), 8.29 (s, 1H), 7.97 (d, *J* = 8.7 Hz, 2H), 7.82 (d, *J* = 1.7 Hz, 1H), 7.76 (d, *J* = 8.7 Hz, 2H), 3.60 (dd, *J* = 9.4, 7.7 Hz, 1H), 3.50 – 3.44 (m, 2H), 3.40 (dt, *J* = 9.0, 7.2 Hz, 1H), 3.33 (q, *J* = 6.4 Hz, 2H), 3.29 – 3.22 (m, 1H), 2.81 (t, *J* = 2.6 Hz, 1H), 2.23 (td, *J* = 7.1, 2.7 Hz, 2H), 2.18 – 1.99 (m, 2H), 1.71 (p, *J* = 7.1 Hz, 2H).

^{13}C NMR (126 MHz, DMSO-d_6) δ (ppm) = 169.12, 165.23, 158.26 (d, *J* = 36.9 Hz), 139.01, 138.70, 132.44, 132.14, 129.00, 119.29, 117.30, 104.30, 84.17, 71.48, 52.56, 50.05, 43.08, 38.52, 29.59, 28.08, 15.57.

HRMS *m/z* for $C_{21}H_{23}N_6O_2^+$ ([M+H]$^+$) calculated: 391.1877, found: 391.1880.

ee >99%, t_R (min) = 50.04.

3.3.2 Synthesis of the minimal probes

(*S*)-1-Cyano-*N*-(pent-4-yn-1-yl)pyrrolidine-3-carboxamide (GK16S)

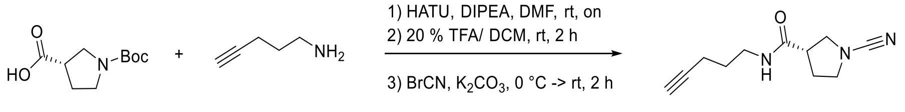

<u>Step 1</u>

(*S*)-*N*-Boc-pyrrolidine-3-carboxylic acid (50 mg, 0.23 mmol, 1.0 eq) was dissolved in 4 mL of DCM, then DIPEA (79 µL, 0.46 mmol, 2.0 eq) and HATU (525 mg, 1.38 mmol, 1.5 eq) were added to the solution.

After stirring for 30 min at room temperature 4-pentyn-1-amine hydrochloride (33 mg, 1.38 mmol, 1.5 eq) was added and the solution was stirred overnight. The reaction mixture was poured into sat. NH_4Cl (50 mL) and extracted with EA (100 mL). The organic layer was washed with brine (100 mL), dried over anhydrous $MgSO_4$, filtered and the solvent was evaporated under reduced pressure. The obtained crude product was used for the next reaction without further purification.

Step 2

The crude product from step 1 was dissolved in 20% TFA in DCM (2 mL) and stirred for 2 h. To this solution toluene (1 mL) was added and the solvent was removed under reduced pressure yielding crude (*S*)-*N*-(pyrrolidin-3-yl)pent-4-ynamide.

Step 3

The crude product from step 2 was dissolved in DCM (4 mL), then K_2CO_3 (130 mg, 0.87 mmol, 4.0 eq) was added and the reaction mixture was stirred for 20 min at room temperature. The pH was adjusted to pH = 7-8 by adding a few drops of 10 M NaOH. The solution was cooled to 0 °C and 3 M cyanogen bromide solution in DCM (106 µL, 0.25 mmol, 1.1 eq) was added dropwise. After 30 min the ice bath was removed, and the reaction mixture was stirred for 2 h at room temperature. The reaction was quenched by the addition of H_2O (50 mL) and extracted with DCM (100 mL). The aqueous phase was extracted with EA (100 mL) and the organic layers were combined and dried over anhydrous $MgSO_4$. After filtering, the solvent was removed under reduced pressure and the product purified via a silica column eluting at 0-1% MeOH in DCM. Pooling of pure fractions yielded GK16S (18.1 mg, 0.09 mmol, 39%) as a clear resin.

^{1}H NMR (700 MHz, DMSO-*d*$_6$) δ (ppm) = 8.04 (t, *J* = 5.6 Hz, 1H), 3.49 (dd, *J* = 9.3, 7.8 Hz, 1H), 3.41 (ddd, *J* = 9.1, 7.9, 5.2 Hz, 1H), 3.38-3.32 (m, 2H), 3.12 (tdd, *J* = 6.7, 5.5, 0.9 Hz, 2H), 2.94 (p, *J* = 7.4 Hz, 1H), 2.78 (t, *J* = 2.7 Hz, 1H), 2.16 (td, *J* = 7.2, 2.7 Hz, 2H), 2.03 (dtd, *J* = 12.6, 7.3, 5.2 Hz, 1H), 1.92 (dq, *J* = 12.5, 7.6 Hz, 1H), 1.57 (p, *J* = 7.1 Hz, 2H).

^{13}C NMR (176 MHz, DMSO-*d*$_6$) δ (ppm) =171.73, 117.76, 84.45, 71.88, 52.96, 50.52, 43.67, 38.25, 29.97, 28.42, 15.84.

HRMS *m/z* for $C_{11}H_{16}N_3O^+$ ($[M+H]^+$) calculated: 206.1288, found: 206.1288.

ee 98.7%, t_R (min) = 39.35.

(*R*)-1-Cyano-*N*-(pent-4-yn-1-yl)pyrrolidine-3-carboxamide (GK16R):

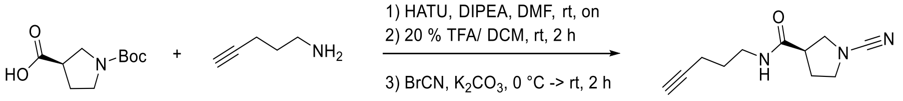

GK16R (24.3 mg, 0.12 mmol, 53%) as obtained as a clear resin from (*R*)-*N*-Boc-pyrrolidine-3-carboxylic acid as described above for GK16S.

^{1}H NMR (700 MHz, DMSO-*d*$_6$) δ (ppm) = 8.03 (t, *J* = 5.6 Hz, 1H), 3.49 (dd, *J* = 9.3, 7.8 Hz, 1H), 3.41 (ddd, *J* = 9.0, 7.8, 5.2 Hz, 1H), 3.38-3.32 (m, 2H), 3.12 (tdd, *J* = 6.7, 5.5, 0.9 Hz, 2H), 2.94 (p, *J* = 7.4 Hz, 1H), 2.77 (t, *J* = 2.7 Hz, 1H), 2.16 (td, *J* = 7.2, 2.7 Hz, 2H), 2.03 (dtd, *J* = 12.6, 7.3, 5.2 Hz, 1H), 1.92 (dq, *J* = 12.5, 7.6 Hz, 1H), 1.57 (p, *J* = 7.1 Hz, 2H).

^{13}C NMR (176 MHz, DMSO-*d*$_6$) δ (ppm) = 171.25, 117.27, 83.96, 71.38, 52.48, 50.04, 43.19, 37.76, 29.48, 27.94, 15.36.

HRMS *m/z* for $C_{11}H_{16}N_3O^+$ ($[M+H]^+$) calculated: 206.1288, found: 206.1288.

ee 94.9%, t_R (min) = 37.63.

3.3.3 Synthesis of parent inhibitor Cpd158

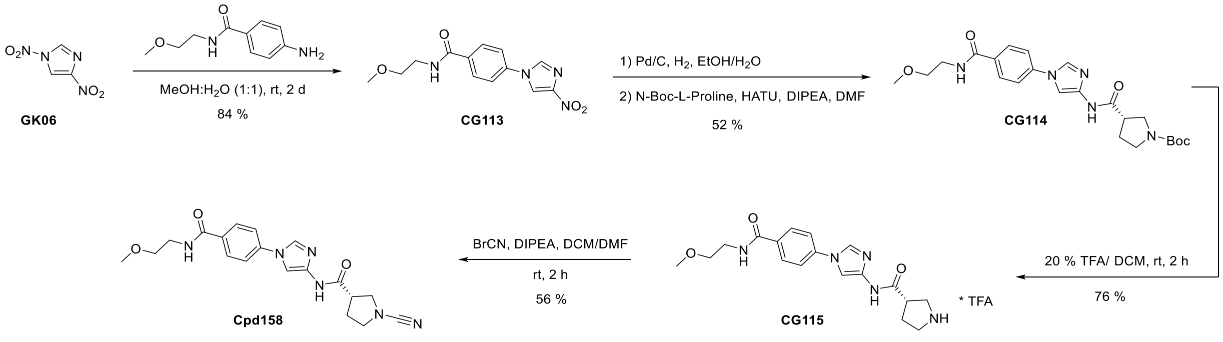

Scheme 3.3.3: Synthesis of compound Cpd158 (compound 158 in patent WO2016046530A1[25]).

N-(2-Methoxyethyl)-4-(4-nitro-1*H*-imidazol-1-yl)benzamide (CG113)

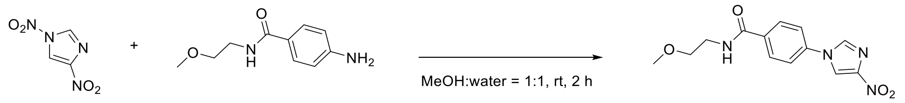

GK06 (300 mg, 1.54 mmol, 1.2 eq) was dissolved in a 1:1 mixture of MeOH and H_2O (10 mL). 4-Amino-*N*-(2-methoxyethyl)benzamide (292.98 mg, 1.85 mmol, 1.0 eq) was added and the reaction mixture was stirred for 2 h at room temperature in darkness. The obtained suspension was cooled down to 0 °C and the precipitate was filtered and washed with cold MeOH in H_2O (1:1). The product was obtained as a yellow solid (377.4 mg, 1.30 mmol, 84% yield).

^{1}H NMR (600 MHz, DMSO-*d*6) δ (ppm) = 9.10 (d, *J* = 1.6 Hz, 1H), 8.69 (t, *J* = 5.3 Hz, 1H), 8.58 (d, *J* = 1.6 Hz, 1H), 8.04 (d, *J* = 8.7 Hz, 2H), 7.94 (d, *J* = 8.7 Hz, 2H), 3.50-3.42 (m, 4H), 3.28 (s, 3H).

^{13}C NMR (151 MHz, DMSO-*d*6) δ (ppm) = 164.96, 148.24, 137.39, 135.65, 134.00, 128.90, 120.78, 119.58, 70.40, 70.38, 57.95, 40.06.

HRMS *m/z* for $C_{13}H_{15}N_4O_4^+$ ([M+H]$^+$) calculated: 291.1088, found: 291.1091.

tert-Butyl (*S*)-3-((1-(4-((2-methoxyethyl)carbamoyl)phenyl)-1*H*-imidazol-4-yl)carbamoyl)pyrrolidine-1-carboxylate (CG114)

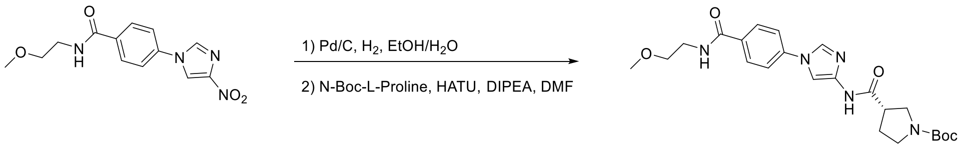

Step 1
CG113 (100 mg, 0.34 mmol, 1.0 eq) was dissolved in EtOH (5 mL) and a few drops of H_2O were added. Palladium (10%) on activated charcoal (20 mg) was added under argon atmosphere. Then the flask was flushed with hydrogen gas and the reaction mixture was stirred for 2 h at room temperature. The reaction was filtered through celite545 and was washed with EA. The solvent was dried over anhydrous $MgSO_4$ and removed under reduced pressure. The obtained yellow solid was used for the next reaction without further purification.

Step 2
(*S*)-1-(*tert*-Butoxycarbonyl)pyrrolidine-3-carboxylic acid (81.6 mg, 0.38 mmol, 1.1 eq) was dissolved in DMF (2 mL) and DIPEA (117 µL, 0.69 mmol, 2.0 eq) and HATU (157 mg, 0.41 mmol, 1.2 eq) were added. The solution was stirred for 30 min at room temperature. The crude product from step 1 was

dissolved in DMF (1 mL) and added dropwise to the reaction mixture. After additional 2 h the reaction mixture was quenched with sat. NH_4Cl-solution (20 ml) and extracted with EA (40 mL). The organic phase was washed with brine (20 mL), dried over anhydrous $MgSO_4$ and the solvent was removed under reduced pressure. The compound was purified via a silica column eluting at 5% MeOH in DCM, yielding CG114 (82 mg, 0.18 mmol, 52%) as a light-brown solid.

^{1}H NMR (600 MHz, DMSO-*d*$_6$) δ (ppm) = 10.67 (d, *J* = 10.0 Hz, 1H), 8.61 (t, *J* = 5.4 Hz, 1H), 8.25 (d, *J* = 1.6 Hz, 1H), 8.03-7.96 (m, 2H), 7.81 (s, 1H), 7.76 (d, *J* = 8.7 Hz, 2H), 3.49 (dd, *J* = 11.1, 4.0 Hz, 1H), 3.48-3.41 (m, 4H), 3.41-3.34 (m, 2H), 3.28 (s, 3H), 3.26-3.14 (m, 2H), 2.09 (td, *J* = 11.7, 5.4 Hz, 1H), 2.06-1.95 (m, 1H), 1.41 (s, 9H).

^{13}C NMR (151 MHz, DMSO-*d*$_6$) δ (ppm) = 169.65, 165.20, 153.36, 139.29, 138.77, 132.04, 128.97, 119.17, 104.07, 78.31, 70.44, 57.93, 48.47, 45.41, 40.74, 33.49, 28.18, 24.10, 21.03.

HRMS *m/z* for $C_{23}H_{32}N_5O_5^+$ ([M+H]$^+$) calculated: 458.2398, found: 458.2393.

(*S*)-*N*-(1-(4-((2-Methoxyethyl)carbamoyl)phenyl)-1*H*-imidazol-4-yl)pyrrolidine-3-carboxamide TFA-salt (CG115)

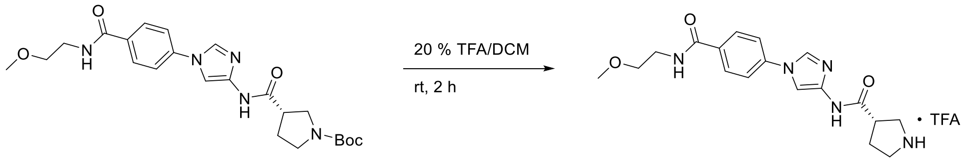

CG114 (75 mg, 0.16 mmol, 1.0 eq) was dissolved in a solution of 20% TFA in DCM (2 mL). The mixture was stirred for 2 h at room temperature. The solvent was removed under reduced pressure and the crude product purified by preparative HPLC eluting at 10-20% ACN, yielding CG115 as a TFA salt (58.7 mg, 0.12 mmol, 76%).

^{1}H NMR (700 MHz, DMSO-*d*$_6$) δ (ppm) = 10.85 (s, 1H), 8.85 (d, *J* = 47.9 Hz, 2H), 8.61 (t, *J* = 5.4 Hz, 1H), 8.27 (d, *J* = 1.6 Hz, 1H), 7.99 (d, *J* = 8.6 Hz, 2H), 7.78 (d, *J* = 1.6 Hz, 1H), 7.74 (d, *J* = 8.6 Hz, 2H), 3.47 (dd, *J* = 6.3, 4.3 Hz, 2H), 3.44 (t, *J* = 5.2 Hz, 2H), 3.43-3.39 (m, 1H), 3.34 (tt, *J* = 11.6, 6.1 Hz, 2H), 3.27 (s, 3H), 3.24 (ddt, *J* = 25.6, 11.8, 3.0 Hz, 2H), 2.24 (dq, *J* = 14.0, 7.2 Hz, 1H), 2.03 (dq, *J* = 13.8, 7.2 Hz, 1H).

^{13}C NMR (176 MHz, DMSO-*d*$_6$) δ (ppm) = 168.87, 165.16, 158.01 (q, *J* = 33.9 Hz), 139.06, 138.69, 132.31, 132.25, 129.00, 119.29, 116.36 (q, *J* = 295.4 Hz), 104.22, 70.44, 57.92, 47.07, 45.10, 41.90, 40.02, 28.88.

HRMS *m/z* for $C_{18}H_{24}N_5O_3^+$ ([M+H]$^+$) calculated: 358.1874, found: 358.1875.

(*S*)-1-Cyano-*N*-(1-(4-((2-methoxyethyl)carbamoyl)phenyl)-1*H*-imidazol-4-yl)pyrrolidine-3-carboxamide (Cpd158)

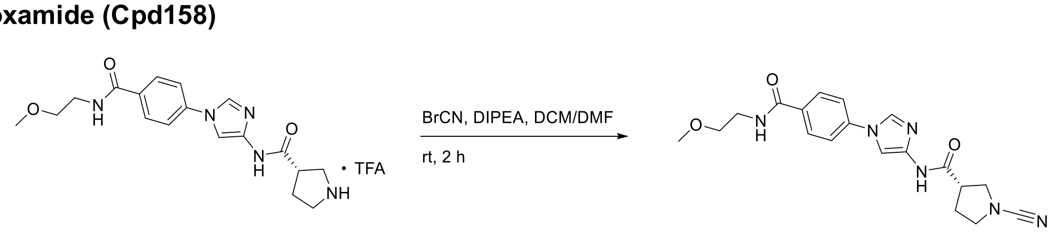

CG115 (40 mg, 0.08 mmol, 1.0 eq) was dissolved in DCM (2 mL) and K_2CO_3 (46.9 mg, 0.34 mmol, 4.0 eq) was added. A 3 M cyanogen bromide solution (34 µL, 0.10 mmol, 1.2 eq) was added and the mixture was stirred for 2 h. The reaction was quenched by the addition of H_2O (10 mL). The two phases were separated, and the aqueous phase were extracted two times with EA (20 mL). The combined

organic layers were dried over anhydrous $MgSO_4$, filtered and the solvent was removed under reduced pressure. The corresponding product was purified via a silica column eluting at 6% MeOH in DCM, yielding Cpd158 (18.2 mg, 0.05 mmol, 56%) as a white solid.

^{1}H NMR (500 MHz, DMSO-*d*$_6$) δ (ppm) = 10.76 (s, 1H), 8.62 (t, *J* = 5.3 Hz, 1H), 8.26 (d, *J* = 1.7 Hz, 1H), 7.98 (d, *J* = 8.7 Hz, 2H), 7.81 (d, *J* = 1.6 Hz, 1H), 7.76 (d, *J* = 8.7 Hz, 2H), 3.60 (dd, *J* = 9.5, 7.7 Hz, 1zH), 3.52-3.37 (m, 7H), 3.27 (s, 3H), 2.14 (dtd, *J* = 12.8, 7.3, 5.6 Hz, 1H), 2.03 (dq, *J* = 12.6, 7.3 Hz, 1H), 1.25 (q, *J* = 6.9 Hz, 1H).

^{13}C NMR (126 MHz, DMSO-*d*$_6$) δ (ppm) = 169.50, 165.65, 139.66, 139.21, 132.63, 132.58, 129.45, 119.66, 117.73, 104.61, 70.91, 58.40, 53.00, 50.49, 43.50, 30.03.

HRMS *m/z* for $C_{19}H_{23}N_6O_3^+$ ($[M+H]^+$) calculated: 383.1826, found: 383.1829.

3.4 Synthesis of 2-carboxypyrrolidine-based probes and controls

3.4.1 Synthesis of parent inhibitor Cpd117

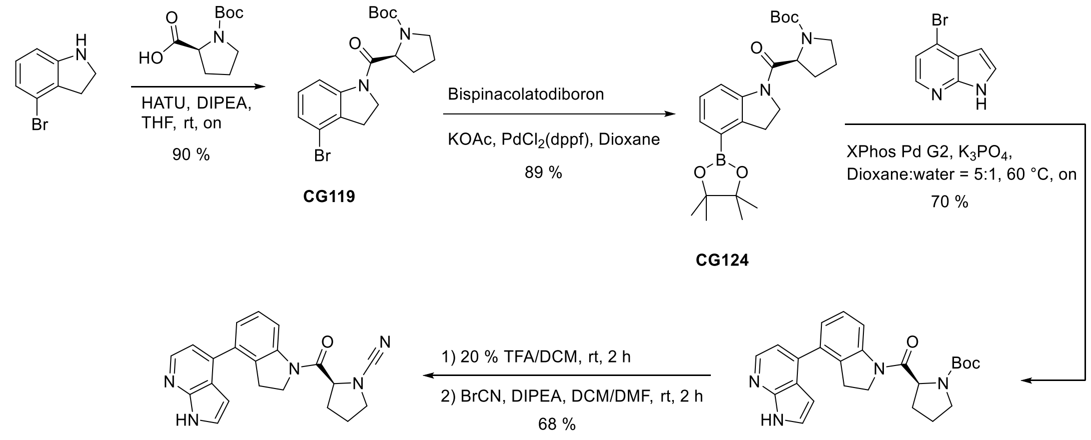

Scheme 3.4.1: Synthesis of compound Cpd117 (compound 117 in patent US20180194724A1[27]).

***tert*-Butyl (*S*)-2-(4-bromoindoline-1-carbonyl)pyrrolidine-1-carboxylate (CG119)**

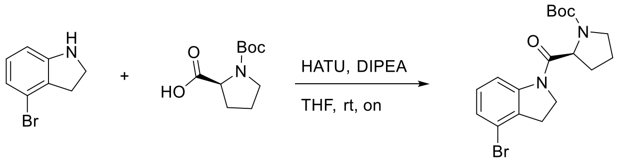

(*tert*-Butoxycarbonyl)-*L*-proline (652 mg, 2.52 mmol, 1.2 eq) was dissolved in 6 mL of THF, then DIPEA (879 µL, 5.05 mmol, 2.0 eq) and HATU (1.44 g, 3.79 mmol, 1.5 eq) were added to the solution. After stirring for 30 min at room temperature 4-bromoindoline (500 mg, 2.52 mmol, 1.0 eq) was added and the solution was stirred overnight. The reaction mixture was poured into sat. $NaHCO_3$ solution (150 mL) and extracted with EA (150 mL). The organic layer was washed with brine (2x100 mL), dried over anhydrous $MgSO_4$, filtered and the solvent was evaporated under reduced pressure. The resulting crude product was purified by column chromatography using a reverse phase silica column. The product was eluting at 60-80% ACN (+0.1% TFA) in H_2O (+0.1% TFA). The fractions containing the product were combined and the solvent removed under reduced pressure yielding CG119 as a white powder (896.4 mg, 2.27 mmol, 90%). The analytical characterization was in good agreement with previously reported data[28]. The data show rotamers within the Boc-protected pyrrolidine which are listed as pseudodoublets for CG119 and all following compounds of the series until deprotection.

^{1}H NMR (500 MHz, $CDCl_3$) δ (ppm) = 8.18 (dd, *J* = 16.5, 8.0 Hz, 1H), 7.16 (ddd, *J* = 18.5, 8.0, 1.0 Hz, 1H), 7.07 (dt, *J* = 22.5, 8.1 Hz, 1H), 4.48 – 4.38 (m, 1H), 4.63 – 4.18 (m, 1H), 4.13 – 4.04 (m, 1H), 3.66 (dddd, *J* = 20.9, 10.2, 7.6, 5.3 Hz, 1H), 3.49 (ddt, *J* = 35.2, 10.5, 7.0 Hz, 1H), 3.28 – 3.14 (m, 2H), 2.37 – 2.05 (m, 2H), 2.06 – 1.82 (m, 1H), 1.40 (d, *J* = 58.8 Hz, 9H).

^{13}C NMR (126 MHz, $CDCl_3$) δ (ppm) = 171.60, 153.66, 143.99, 131.43, 129.56, 126.64, 119.24, 115.83, 79.89, 58.45, 47.14, 46.72, 30.46, 29.73, 28.51, 23.75.

HRMS *m/z* for $C_{18}H_{24}BrN_2O_3^+$ ($[M+H]^+$) calculated: 395.0965, found: 395.0965.

***tert*-Butyl (*S*)-2-(4-(4,4,5,5-tetramethyl-1,3,2-dioxaborolan-2-yl)indoline-1-carbonyl)pyrrolidine-1-carboxylate (CG124)**

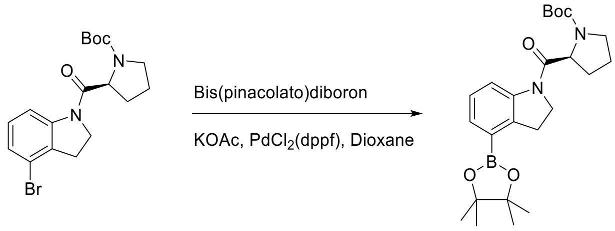

To a solution of CG119 (1.70 g, 4.30 mmol, 1.0 eq) in 1,4-dioxane (20 mL) were added bis(pinacolato)diboron (1.20 g, 4.73 mmol, 1.1 eq) and potassium acetate (1.27 g, 12.90 mmol, 3.0 eq). The reaction mixture was degassed for 30 min at room temperature by bubbling the solution with argon. Pd(dppf)Cl_2 (314.7 mg, 0.43 mmol, 0.1 eq) was added to the mixture and the reaction was heated at 100 °C for 1 h. The resulting solution was poured into H_2O (200 mL) and extracted with EA (2x200 mL). The combined organic layer was dried over anhydrous $MgSO_4$, filtered and concentrated under reduced pressure. The crude compound was purified via silica normal phase column chromatography, eluting at 12% EA in PE yielding CG124 (1.695 g, 3.83 mmol, 89%). The analytical characterization was in good agreement with previously reported data[28].

^{1}H NMR (500 MHz, MeOH-*d$_4$*) δ (ppm) = 8.34-8.21 (m, 1H), 7.43 (td, *J* = 7.4, 1.1 Hz, 1H), 7.18 (dt, *J* = 13.7, 7.8 Hz, 1H), 4.62 (td, *J* = 8.7, 8.2, 4.5 Hz, 1H), 4.39-4.01 (m, 2H), 3.58 (dt, *J* = 9.9, 6.7 Hz, 1H), 3.54-3.46 (m, 1H), 3.45-3.36 (m, 2H), 2.48-2.28 (m, 1H), 2.10-2.03 (m, 1H), 2.02-1.87 (m, 2H), 1.47 (s, 3H), 1.35 (s, 12H), 1.32 (s, 6H).

^{13}C NMR (126 MHz, MeOH-*d$_4$*) δ (ppm) = 173.45, 156.26, 155.68, 143.55, 143.42, 140.25, 132.00, 127.66, 120.69, 85.04, 81.41, 60.29, 47.90, 31.34, 30.36, 28.51, 25.27, 24.87.

HRMS *m/z* for $C_{24}H_{36}BN_2O_5^+$ ([M+H]$^+$) calculated: 443.2712, found: 443.2711.

***tert*-Butyl (*S*)-2-(4-(1*H*-pyrrolo[2,3-*b*]pyridin-4-yl)indoline-1-carbonyl)pyrrolidine-1-carboxylate (CG127)**

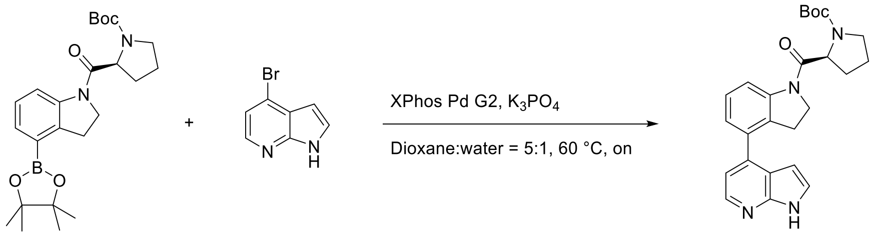

To a solution of 4-bromo-1*H*-pyrrolo[2,3-*b*]pyridine (50 mg, 0.25 mmol, 1.0 eq) and CG124 (123.5 mg, 0.28 mmol, 1.1 eq) in 2.4 mL of a 5:1 mixture of 1,4-dioxane:H_2O was added K_3PO_4 (107.7 mg, 0.51 mmol, 2.0 eq) at room temperature under argon atmosphere. The reaction mixture was degassed performing three iterative freeze-pump-thaw cycles. Xphos Pd G2 (3.0 mg, 3.81 µmol, 0.02 eq) was added to the reaction mixture and it was stirred overnight at 60 °C under argon atmosphere. The resulting reaction mixture was poured into sat. $NaHCO_3$ solution (40 mL) and extracted with EA (3x50 mL). The combined organic layers were dried over anhydrous $MgSO_4$, filtered and concentrated under reduced pressure. The crude compound was purified via silica normal phase column chromatography, eluting at 30-40% EA in PE yielding CG127 (77.0 mg, 0.18 mmol, 70%).

^{1}H NMR (700 MHz, DMSO-*d$_6$*) δ (ppm) = 11.78 (s, 1H), 8.27 (d, *J* = 4.8 Hz, 1H), 8.20 (dd, *J* = 19.6, 8.1 Hz, 1H), 7.51 (td, *J* = 3.6, 2.5 Hz, 1H), 7.34 (dt, *J* = 15.1, 7.8 Hz, 1H), 7.19-7.14 (m, 1H), 7.08 (t, *J* = 4.9 Hz, 1H), 4.60-4.46 (m, 1H), 4.33-4.08 (m, 2H), 3.44 (dt, *J* = 9.8, 6.5 Hz, 1H), 3.17 (dt, *J* = 17.4, 9.2 Hz,

1H), 3.08 (tdd, J = 16.0, 10.2, 6.2 Hz, 1H), 1.99-1.82 (m, 2H), 1.40 (s, 4H), 1.27 (s, 5H), 1.27-1.21 (m, 4H).

^{13}C NMR (176 MHz, DMSO-d_6) δ (ppm) = 171.64, 153.96, 153.47, 149.35, 144.04, 143.01, 139.81, 135.87, 130.43, 128.17, 127.00, 124.55, 118.53, 115.52, 99.67, 78.99, 66.82, 58.92, 47.00, 30.32, 28.37, 24.57, 21.50.

HRMS *m/z* for $C_{25}H_{29}N_4O_3^+$ ([M+H]$^+$) calculated: 433.2234, found: 433.2232.

(*S*)-2-(4-(1*H*-Pyrrolo[2,3-*b*]pyridin-4-yl)indoline-1-carbonyl)pyrrolidine-1-carbonitrile (Cpd117)

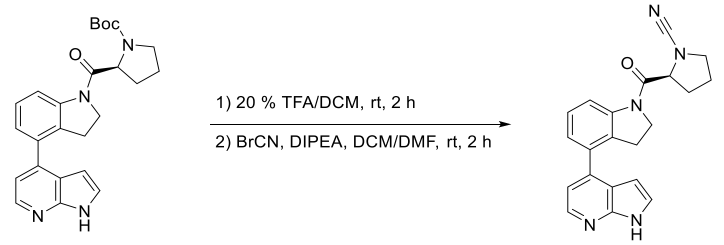

Step 1
CG-127 (19.4 mg, 0.04 mmol, 1.0 eq) was dissolved in 20% TFA in DCM (2 mL) and stirred for 2 h. To this solution toluene (2 mL) was added and the solvent was removed under reduced pressure.

Step 2
The crude product from step 1 was dissolved in DCM (2 mL) and DMF (1 mL), then DIPEA (23.4 µL, 0.13 mmol, 3.0 eq) was added and the reaction mixture was stirred for 20 min at room temperature. The solution was cooled to 0 °C and 3 M cyanogen bromide solution in DCM (17.9 µL, 0.05 mmol, 1.2 eq) was added dropwise. After 30 min the ice bath was removed, and the reaction mixture was stirred for 2 h at room temperature. The reaction was quenched by the addition of H_2O (50 mL) and extracted with DCM (3x50 mL). The aqueous phase was extracted with EA (3x50 mL) and the organic layers were combined and dried over anhydrous $MgSO_4$. After filtering, the solvent was removed under reduced pressure and the product purified via a silica column eluting at 20-30% MeOH in DCM. Pure fractions were pooled, yielding Cpd117 (10.9 mg, 0.03 mmol, 68%) as a clear resin. The analytical characterization was in good agreement with previously reported data[28].

^{1}H NMR (500 MHz, $CDCl_3$) δ (ppm) = 10.12 (s, 1H), 8.36 (d, J = 7.7 Hz, 2H), 7.45-7.33 (m, 2H), 7.24 (dd, J = 7.6, 1.0 Hz, 1H), 7.05 (d, J = 4.7 Hz, 1H), 6.40 (d, J = 3.5 Hz, 1H), 4.50 (dd, J = 8.2, 3.5 Hz, 1H), 4.22 (td, J = 9.9, 6.8 Hz, 1H), 4.05 (td, J = 9.9, 6.4 Hz, 1H), 3.77 (td, J = 7.8, 5.5 Hz, 1H), 3.61-3.48 (m, 1H), 3.18 (dddd, J = 34.4, 16.8, 10.2, 6.6 Hz, 2H), 2.30 (dtd, J = 11.9, 7.7, 3.3 Hz, 1H), 2.24-2.11 (m, 2H), 2.08-1.95 (m, 1H).

^{13}C NMR (126 MHz, $CDCl_3$) δ (ppm) = 168.03, 143.26, 142.59, 141.26, 135.56, 129.52, 128.39, 125.60, 125.43, 119.14, 117.45, 116.40, 115.86, 100.79, 62.46, 51.69, 48.06, 41.12, 30.28, 28.10, 24.52.

HRMS *m/z* for $C_{21}H_{20}N_5O^+$ ([M+H]$^+$) calculated: 358.1662, found: 358.1664.

3.4.2 Synthesis of probe CG173

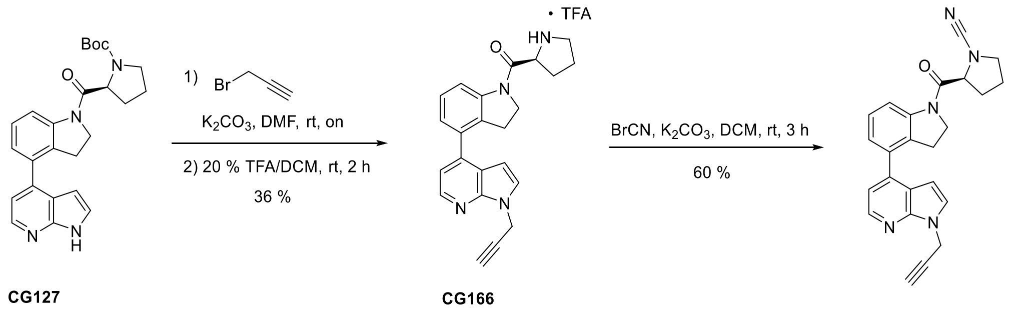

Scheme 3.4.2: Synthesis of compound CG173 based on [27].

(*S*)-4-(1-Prolylindolin-4-yl)-1-(prop-2-yn-1-yl)-1*H*-pyrrolo[2,3-b]pyridine TFA salt (CG166)

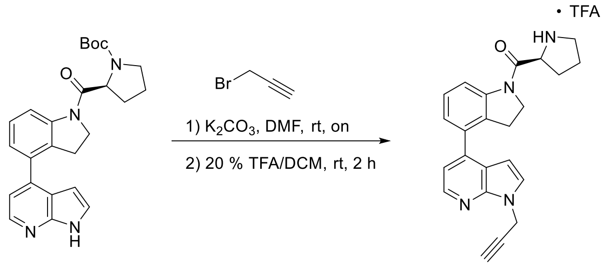

Step 1
To a solution of CG127 (77 mg, 0.18 mmol, 1.0 eq) and K_2CO_3 (49.2 mg, 0.36 mmol, 2.0 eq) in DMF (2 mL) was added propargyl bromide (23.8 µL (80% in toluene), 0.21 mmol, 1.2 eq) dropwise at room temperature. After stirring overnight, the reaction mixture was poured into H_2O (50 mL) and extracted with EA (3x50 mL). The combined organic layers were washed with brine (50 mL), dried over anhydrous $MgSO_4$, filtered and concentrated under reduced pressure. The crude product was directly used for the next reaction.

Step 2
The crude product from step 2 was dissolved in 20% TFA in DCM (2 mL) and stirred for 2 h. The solvent was removed under reduced pressure and the corresponding product purified via preparative HPLC eluting at 50-60% ACN yielding CG166 as a TFA salt (31 mg, 0.06 mmol, 36%).

^{1}H NMR (700 MHz, $CDCl_3$) δ (ppm) = 9.43-9.39 (m, 1H), 8.79-8.71 (m, 1H), 8.37 (d, *J* = 4.8 Hz, 1H), 8.20 (d, *J* = 8.1 Hz, 1H), 7.68 (d, *J* = 3.6 Hz, 1H), 7.43 (t, *J* = 7.9 Hz, 1H), 7.26 (d, *J* = 7.6 Hz, 1H), 7.18 (d, *J* = 4.8 Hz, 1H), 6.39 (d, *J* = 3.5 Hz, 1H), 5.17 (d, *J* = 2.5 Hz, 2H), 4.64-4.58 (m, 1H), 4.22 (td, *J* = 10.0, 6.2 Hz, 1H), 4.10 (td, *J* = 10.0, 6.8 Hz, 1H), 3.39 (t, *J* = 2.5 Hz, 1H), 3.35 (d, *J* = 5.9 Hz, 1H), 3.23 (dt, *J* = 17.8, 6.6 Hz, 1H), 3.20-3.07 (m, 2H), 2.47 (dd, *J* = 8.7, 5.9 Hz, 1H), 1.99 (dddd, *J* = 22.2, 20.0, 9.9, 4.3 Hz, 3H).

^{13}C NMR (176 MHz, $CDCl_3$) δ (ppm) = 166.68, 157.84 (q, J = 32.3 Hz), 147.07, 142.86, 139.72, 135.20, 130.62, 128.91, 127.85, 125.11, 118.23, 117.66, 116.18, 115.69, 99.25, 79.43, 75.14, 59.20, 47.48, 45.95, 33.28, 28.04, 27.39, 23.63.

HRMS *m/z* for $C_{23}H_{23}N_4O^+$ ($[M+H]^+$) calculated: 371.1866, found: 371.1867.

(*S*)-2-(4-(1-(Prop-2-yn-1-yl)-1*H*-pyrrolo[2,3-*b*]pyridin-4-yl)indoline-1-carbonyl)pyrrolidine-1-carbonitrile (CG173)

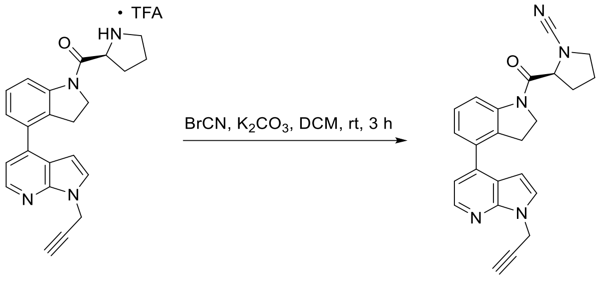

CG166 (27 mg, 0.06 mmol, 1.0 eq) was dissolved in DCM (2 mL), then K_2CO_3 (30.8 mg, 0.22 mmol, 4.0 eq) was added and the reaction mixture was stirred for 20 min at room temperature. The solution was cooled to 0 °C and 3 M cyanogen bromide solution in DCM (22.3 µL, 0.07 mmol, 1.2 eq) was added dropwise. After 30 min the ice bath was removed and the reaction mixture was stirred for 3 h at room temperature. The reaction was quenched by the addition of H_2O (50 mL) and extracted with DCM (3x50 mL). The aqueous phase was extracted with EA (3x50 mL) and the organic layers were combined and dried over anhydrous $MgSO_4$. After filtering, the solvent was removed under reduced pressure and the product purified via a silica column eluting at 2-3% MeOH in DCM. Pooling of pure fractions yielded CG173 (13.3 mg, 0.03 mmol, 60%) as a clear resin.

^{1}H NMR (500 MHz, $CDCl_3$) δ (ppm) = 8.39 (d, *J* = 4.9 Hz, 1H), 8.35 (d, *J* = 8.1 Hz, 1H), 7.45 (d, *J* = 3.6 Hz, 1H), 7.35 (t, *J* = 7.9 Hz, 1H), 7.21 (dd, *J* = 7.6, 1.1 Hz, 1H), 7.03 (d, *J* = 4.8 Hz, 1H), 6.39 (d, *J* = 3.6 Hz, 1H), 5.14 (d, *J* = 2.6 Hz, 2H), 4.50 (dd, *J* = 8.2, 3.5 Hz, 1H), 4.21 (td, *J* = 10.0, 6.8 Hz, 1H), 4.03 (td, *J* = 10.0, 6.4 Hz, 1H), 3.76 (td, *J* = 8.0, 5.5 Hz, 1H), 3.64-3.53 (m, 1H), 3.29-3.07 (m, 2H), 2.42 (t, *J* = 2.6 Hz, 1H), 2.30 (ddt, *J* = 15.4, 7.9, 3.6 Hz, 1H), 2.15 (dddd, *J* = 14.7, 9.0, 7.4, 4.2 Hz, 2H), 2.01 (dddd, *J* = 13.7, 10.8, 5.9, 2.3 Hz, 1H).

^{13}C NMR (126 MHz, $CDCl_3$) δ (ppm) = 168.01, 147.56, 143.23, 143.16, 140.92, 135.49, 129.51, 128.34, 127.59, 125.42, 119.35, 117.36, 116.38, 116.04, 100.21, 78.14, 73.47, 62.45, 51.67, 48.03, 33.94, 30.27, 28.08, 24.50.

HRMS *m/z* for $C_{24}H_{22}N_5O^+$ ($[M+H]^+$) calculated: 396.1819, found: 396.1812.

3.4.3 Synthesis of the 2-carboxypyrrolidine-based minimal probe

(*S*)-1-Cyano-*N*-(pent-4-yn-1-yl)pyrrolidine-2-carboxamide (HK01S)

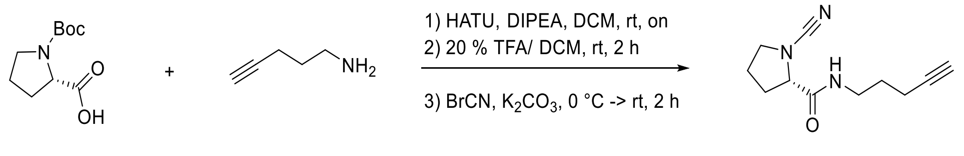

<u>Step 1</u>

(*tert*-Butoxycarbonyl)-*L*-proline (50 mg, 0.23 mmol, 1.0 eq) was dissolved in DCM (4 mL), then DIPEA (162 µL, 0.93 mmol, 4.0 eq) and HATU (132 mg, 0.35 mmol, 1.5 eq) were added to the solution. After stirring for 30 min at room temperature, 4-pentyn-1-amine hydrochloride (33 mg, 0.28 mmol, 1.2 eq) was added and the solution was stirred overnight. The reaction mixture was washed with sat. NH_4Cl solution (50 mL) and extracted with EA (100 mL). The organic layer was washed with brine (3x50 mL), dried over anhydrous $MgSO_4$, filtered and the solvent was evaporated under reduced pressure. The obtained crude product was used for the next reaction without further purification.

Step 2

The crude product from step 1 was dissolved in 20% TFA in DCM (4 mL) and stirred for 2 h. To this solution toluene (1 mL) was added and the solvent was removed under reduced pressure yielding crude (*S*)-*N*-(pent-4-yn-1-yl)pyrrolidine-2-carboxamide.

Step 3

The crude product from step 2 was dissolved in DCM (4 mL), then K_2CO_3 (128 mg, 0.93 mmol, 4.0 eq) was added and the reaction mixture was stirred for 20 min at room temperature. The solution was cooled to 0 °C and 3 M cyanogen bromide solution in DCM (93 µL, 0.28 mmol, 1.2 eq) was added dropwise. After 30 min the ice bath was removed, and the reaction mixture was stirred for 2 h at room temperature. The reaction was quenched by the addition of H_2O (50 mL) and extracted with DCM (3x50 mL). The aqueous phase was extracted with EA (3x50 mL) and the organic layers were combined and dried over anhydrous $MgSO_4$. After filtering, the solvent was removed under reduced pressure and the product purified via a silica column eluting at 0-2% MeOH in DCM, yielding HK01S (14.2 mg, 0.07 mmol, 30%) as a clear resin.

^{1}H NMR (700 MHz, $CDCl_3$) δ (ppm) = 6.69 (s, 1H), 4.12 (dd, *J* = 8.6, 4.0 Hz, 1H), 3.64-3.59 (m, 1H), 3.45 (q, *J* = 8.1 Hz, 1H), 3.41 (q, *J* = 6.6 Hz, 2H), 2.28-2.21 (m, 3H), 2.19-2.14 (m, 1H), 2.02 (d, *J* = 2.9 Hz, 1H), 2.00-1.94 (m, 1H), 1.87 (dt, *J* = 13.0, 7.7 Hz, 1H), 1.77 (p, *J* = 7.0 Hz, 2H).

^{13}C NMR (176 MHz, $CDCl_3$) δ (ppm) = 169.84, 116.43, 83.24, 69.40, 65.12, 52.47, 38.85, 30.83, 27.79, 24.62, 16.12.

HRMS *m/z* for $C_{11}H_{16}N_3O^+$ ($[M+H]^+$) calculated: 206.1288, found 206.1287.

3.5 Synthesis of compound CG050

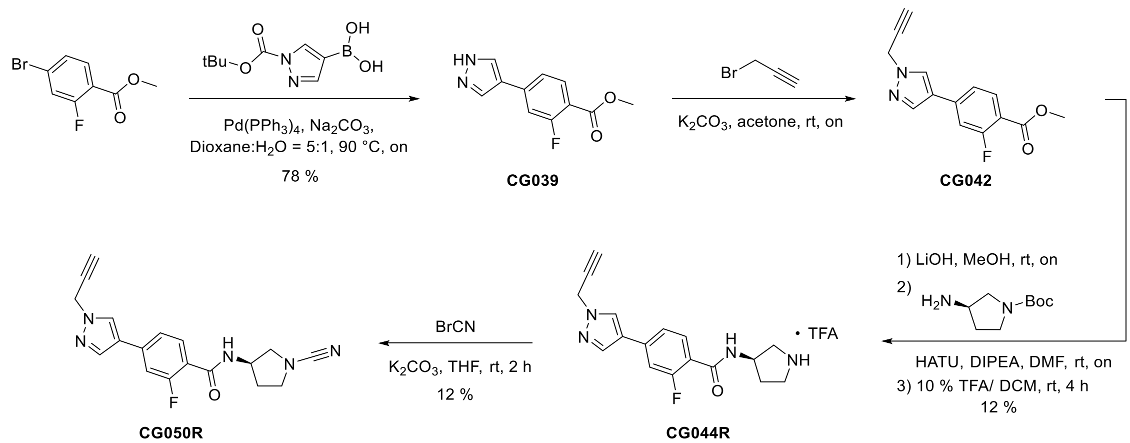

Scheme 3.5: Synthesis of compound CG050 based on [29].

Methyl 2-fluoro-4-(1*H*-pyrazol-4-yl)benzoate (CG039)

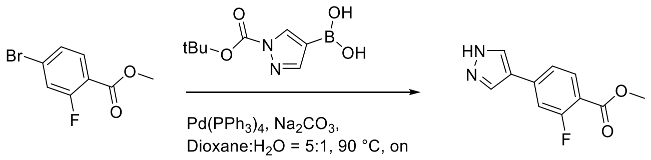

To a solution of methyl 4-bromo-2-fluorobenzoate (500 mg, 2.15 mmol, 1.0 eq), (1-(*tert*-butoxycarbonyl)-1*H*-pyrazol-4-yl)boronic acid (500 mg, 2.36 mmol, 1.1 eq) in 12 mL of a 5:1 mixture of 1,4-dioxane and H_2O was added Na_2CO_3 (750 mg, 7.08 mmol, 3.3 eq) at room temperature. Tetrakis(triphenylphosphin)palladium(0) (99.2 mg, 85.8 µmol, 0.04 eq) was added and the reaction mixture was stirred overnight at 90 °C under argon atmosphere. The resulting reaction mixture was poured into H_2O (200 mL) and extracted with EA (200 mL). The combined organic layers were washed with brine (150 mL), dried over anhydrous $MgSO_4$, filtered and concentrated under reduced pressure. The compound was purified via a silica column eluting at 10% MeOH in DCM yielding CG039 (370 mg, 1.68 mmol, 78%). The analytical characterization was in agreement with previously reported data[30].

^{1}H NMR (500 MHz, DMSO-d_6) δ (ppm) = 8.26 (s, 2H), 7.86 (t, *J* = 8.1 Hz, 1H), 7.64 (dd, *J* = 12.8, 1.7 Hz, 1H), 7.59 (dd, *J* = 8.2, 1.7 Hz, 1H), 3.84 (s, 3H).

^{13}C NMR (126 MHz, DMSO-d_6) δ (ppm) = 163.36 (d, *J* = 132.6 Hz), 160.80, 145.34, 140.37, 132.38, 120.81 (d, *J* = 1.9 Hz), 119.30, 114.59 (d, *J* = 10.0 Hz), 112.80 (d, *J* = 23.5 Hz), 52.20.

HRMS *m/z* for $C_{11}H_{10}FN_2O_2^+$ ($[M+H]^+$) calculated: 221.0721, found: 221.0717.

Methyl 2-fluoro-4-(1-(prop-2-yn-1-yl)-1*H*-pyrazol-4-yl)benzoate (CG042)

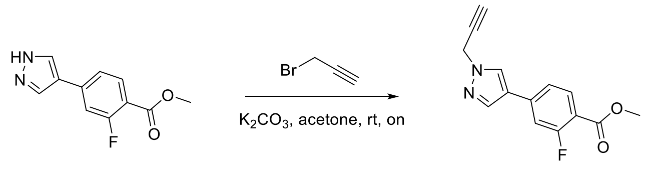

A solution of CG039 (350 mg, 1.59 mmol, 1.0 eq) and K_2CO_3 (439 mg, 3.18 mmol, 2.0 eq) in 4 mL DMF was cooled to 0 °C. To this mixture was added propargyl bromide (265.6 µL (80% in toluene), 2.38 mmol, 1.5 eq) dropwise at 0 °C. After stirring for 30 min the ice bath was removed and the reaction

was stirred at room temperature overnight. The resulting reaction mixture was poured into H_2O (50 mL) and extracted with EA (100 mL). The combined organic layers were washed with brine (100 mL), dried over anhydrous $MgSO_4$, filtered and concentrated under reduced pressure. The compound was purified via a silica column eluting at 20% EA in PE yielding CG042 (236 mg, 0.91 mmol, 57%).

^{1}H NMR (500 MHz, $CDCl_3$) δ (ppm) = 7.95 (s, 1H), 7.93 (t, *J* = 7.9 Hz, 1H), 7.84 (d, *J* = 0.9 Hz, 1H), 7.31 (dd, *J* = 8.2, 1.7 Hz, 1H), 7.23 (dd, *J* = 12.0, 1.7 Hz, 1H), 4.99 (d, *J* = 2.6 Hz, 2H), 3.93 (s, 3H), 2.58 (s, 1H).

^{13}C NMR (126 MHz, $CDCl_3$) δ (ppm) = 164.23 (d, *J* = 148.6 Hz), 161.57, 139.24 (d, *J* = 9.5 Hz), 137.93, 132.98 (d, *J* = 1.8 Hz), 126.81, 121.78, 120.85 (d, *J* = 3.3 Hz), 116.17 (d, *J* = 10.0 Hz), 113.48 (d, *J* = 23.7 Hz), 76.25, 75.55, 52.41, 42.09.

HRMS *m/z* for $C_{14}H_{12}FN_2O_2^+$ ($[M+H]^+$) calculated: 259.0877, found: 259.0874.

(*R*)-2-Fluoro-4-(1-(prop-2-yn-1-yl)-1*H*-pyrazol-4-yl)-*N*-(pyrrolidin-3-yl)benzamide (CG044R)

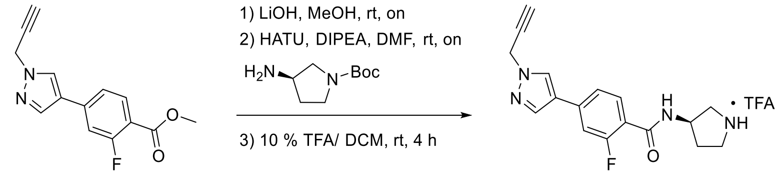

<u>Step 1</u>
To a solution of CG042 (88 mg, 0.34 mmol, 1.0 eq) in MeOH (2 mL) was added 350 µL 2 N LiOH solution and the mixture was stirred overnight at room temperature. The pH of the solution was adjusted to pH 7 and the solvent evaporated under reduced pressure to yield crude lithium 2-fluoro-4-(1-(prop-2-yn-1-yl)-1*H*-pyrazol-4-yl)benzoate.

<u>Step 2</u>
The crude product from step 1 was dissolved in DMF (2 mL), then HATU (194 mg, 0.51 mmol, 1.5 eq) and DIPEA (194 mg, 0.51 mmol, 1.5 eq) were added and the solution was stirred for 30 min at room temperature. Then *tert*-butyl (*R*)-3-aminopyrrolidine-1-carboxylate (70 mg, 0.37 mmol, 1.1 eq) was added and the solution was stirred overnight. The reaction mixture was poured into sat. NH_4Cl solution (50 mL) and extracted with EA (100 mL). The organic layer was washed with brine (100 mL), dried over anhydrous $MgSO_4$, filtered and the solvent was evaporated under reduced pressure to yield crude *tert*-butyl (*R*)-3-(2-fluoro-4-(1-(prop-2-yn-1-yl)-1*H*-pyrazol-4-yl)benzamido)pyrrolidine-1-carboxylate.

<u>Step 3</u>
The crude product from step 2 was dissolved in 20% TFA in DCM (4 mL) and stirred for 4 h. To this solution was added toluene (1 mL) and the solvent was removed under reduced pressure. The corresponding product was purified via preparative HPLC eluting at 18-26% ACN yielding CG044R as a TFA salt (12.7 mg, 0.04 mmol, 12%).

^{1}H NMR (500 MHz, DMSO-*d*$_6$) δ (ppm) = 8.91 (s, 1H), 8.54 (dd, *J* = 6.5, 1.9 Hz, 1H), 8.47 (s, 1H), 8.21 (s, 1H), 7.66 (dd, *J* = 12.2, 1.6 Hz, 1H), 7.63 (t, *J* = 7.8 Hz, 1H), 7.59 (dd, *J* = 8.0, 1.6 Hz, 1H), 7.51 (t, *J* = 6.5 Hz, 1H), 5.81 (d, *J* = 6.5 Hz, 2H), 4.51 (q, *J* = 6.3, 5.6 Hz, 2H), 3.48-3.41 (m, 1H), 3.34 (q, *J* = 6.2, 5.8 Hz, 1H), 3.26 (dtd, *J* = 13.2, 7.7, 6.4, 3.7 Hz, 1H), 3.15 (dq, *J* = 11.5, 5.5 Hz, 1H), 2.20 (dq, *J* = 13.3, 7.5 Hz, 1H), 2.02-1.89 (m, 1H).

^{13}C NMR (126 MHz, DMSO-*d*$_6$) δ (ppm) = 163.82, 159.87 (d, *J* = 248.8 Hz), 138.71, 136.68 (d, *J* = 9.3 Hz), 130.78 (d, *J* = 3.4 Hz), 126.57, 121.75, 120.92, 115.73, 112.45 (d, *J* = 23.7 Hz), 100.96, 89.11, 49.33, 49.25, 48.77, 44.01, 29.78.

HRMS *m/z* for $C_{17}H_{18}FN_4O^+$ ($[M+H]^+$) calculated: 313.1459, found: 313.1461.

(R)-N-(1-Cyanopyrrolidin-3-yl)-2-fluoro-4-(1-(prop-2-yn-1-yl)-1H-pyrazol-4-yl)benzamide (CG050R)

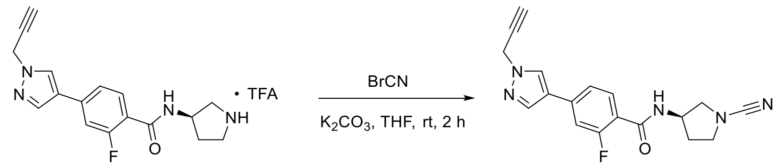

CG044R (171 mg, 0.40 mmol, 1.0 eq) and K_2CO_3 (166 mg, 1.20 mmol, 3.0 eq) were dissolved in DCM (4 mL) and stirred for 20 min at room temperature. The solution was cooled to 0 °C and 3 M cyanogen bromide solution (160 µL, 0.48 mmol, 1.2 eq) was added dropwise. After 30 min the ice bath was removed, and the reaction mixture was stirred for 2 h at room temperature. The reaction was quenched by the addition of H_2O (100 mL) and extracted with DCM (100 mL). The aqueous phase was extracted with EA (100 mL) and the organic layers were combined and dried over $MgSO_4$. After filtering the solvent was removed under reduced pressure and the product purified via a silica column eluting at 1-3% MeOH in DCM. CG050R (16.2 mg, 0.05 mmol, 12%) was obtained as a clear resin.

^{1}H NMR (500 MHz, DMSO-d_6) δ (ppm) = 8.57 (dd, J = 6.7, 1.3 Hz, 1H), 8.40 (d, J = 0.8 Hz, 1H), 8.08 (d, J = 0.8 Hz, 1H), 7.63-7.55 (m, 2H), 7.52 (dd, J = 8.0, 1.6 Hz, 1H), 5.07 (d, J = 2.6 Hz, 2H), 4.53-4.38 (m, 1H), 3.63 (dd, J = 9.8, 6.3 Hz, 1H), 3.56 (t, J = 2.5 Hz, 1H), 3.52 (dt, J = 9.1, 7.4 Hz, 1H), 3.45 (ddd, J = 9.2, 8.1, 5.3 Hz, 1H), 3.33-3.27 (m, 1H), 2.12 (dtd, J = 12.7, 7.8, 6.3 Hz, 1H), 1.92 (ddt, J = 12.5, 7.5, 5.1 Hz, 1H).

^{13}C NMR (126 MHz, DMSO-d_6) δ (ppm) = 163.82, 159.87 (d, J = 248.8 Hz), 158.24 (q, J = 32.2 Hz), 138.71, 136.68 (d, J = 9.3 Hz), 130.78 (d, J = 3.4 Hz), 126.57, 121.75, 120.92, 120.81, 116.92 (d, J = 297.9 Hz), 112.45 (d, J = 23.7 Hz), 100.96, 89.11, 49.33, 49.25, 48.77, 44.01, 29.78.

HRMS *m/z* for $C_{18}H_{17}FN_5O^+$ ($[M+H]^+$) calculated: 338.1412, found: 338.1414.

ee 96.9%, t_R (min) = 50.57.

3.6 Synthesis of compound MS37

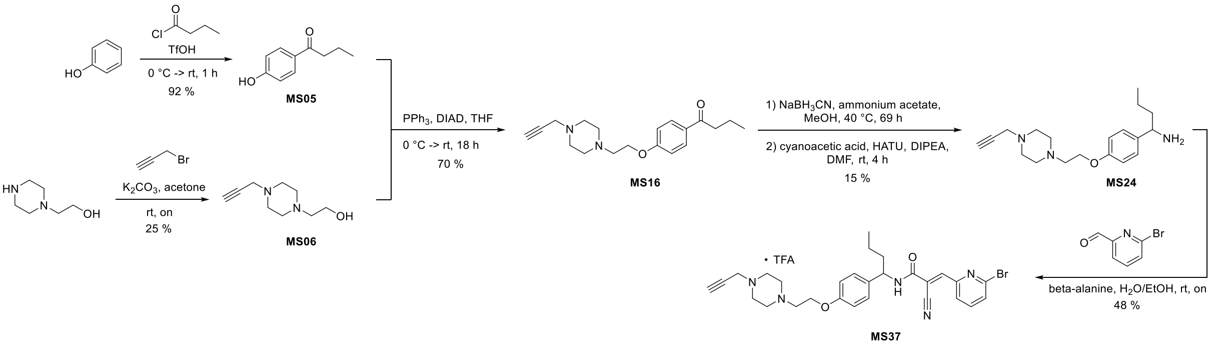

Scheme 3.6: Synthesis of compound MS37 based on [31].

1-(4-Hydroxyphenyl)butan-1-one (MS05)

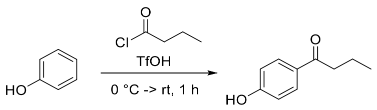

Phenol (500 mg, 5.31 mmol, 1.0 eq) was dissolved in neat triflic acid (TfOH, 4 mL) at 0 °C. The solution was stirred for 10 min. Butyryl chloride (550 µL, 5.31 mmol, 1.0 eq) was added dropwise at 0 °C. The ice bath was removed, and the reaction mixture was stirred for 1 h at room temperature. The reaction mixture was then poured into cold H_2O and extracted with EA (3x10 mL). The organic layers were combined and washed with aq. 1 M HCl solution (1x10 mL), sat. $NaHCO_3$ (1x10 mL) and brine (1x10 mL). The organic phase was dried over $MgSO_4$ and the solvent was evaporated under reduced pressure. The crude product was purified by column chromatography (0-40% EA in PE) to yield MS05 (800 mg, 4.87 mmol, 92%) as a white solid. The analytical characterization was in good agreement with previously reported data[31].

^{1}H NMR (600 MHz, DMSO-*d*$_6$) δ (ppm) = 10.30 (s, 1H), 7.87-7.82 (m, 2H), 6.87-6.82 (m, 2H), 2.88 (t, *J* = 7.2 Hz, 2H), 1.61 (h, *J* = 7.3 Hz, 2H), 0.92 (t, *J* = 7.4 Hz, 3H).

^{13}C NMR (151 MHz, DMSO-*d*$_6$) δ (ppm) = 198.11, 161.85, 130.39, 128.42, 115.17, 39.52, 17.54, 13.73.

LC-MS *m/z* for $C_{10}H_{13}O_2^+$ ($[M+H]^+$) calculated: 165.08, found: 165.10.

2-(4-(Prop-2-yn-1-yl)piperazin-1-yl)ethan-1-ol (MS06)

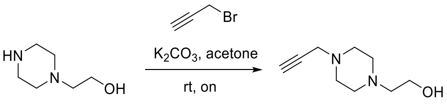

2-(Piperazin-1-yl)ethan-1-ol (50 mg, 0.38 mmol, 1.0 eq) was dissolved in acetone (2 mL). K_2CO_3 (52 mg, 0.38 mmol, 1.0 eq) was added and the reaction mixture was cooled to 0 °C. Propargyl bromide (32 µL, 0.38 mmol, 1.0 eq) was dissolved in acetone (2 mL) and added dropwise to the reaction mixture. The ice bath was removed, and the reaction mixture was allowed to stir overnight at room temperature. The solvent was evaporated under reduced pressure. The crude product was purified by column chromatography (aluminum oxide, 0-10% MeOH in DCM) without aqueous work up, to yield MS06 (16.4 mg, 0.1 mmol, 25%) as a light-yellow solid.

^{1}H NMR (400 MHz, DMSO-*d*$_6$) δ (ppm) = 4.35 (t, *J* = 5.4 Hz, 1H), 3.47 (td, *J* = 6.3, 5.3 Hz, 2H), 3.22 (d, *J* = 2.4 Hz, 2H), 3.12 (t, *J* = 2.4 Hz, 1H), 2.48-2.30 (m, 10H).

^{13}**C NMR** (101 MHz, DMSO-*d*$_6$) δ (ppm) = 79.45, 75.57, 60.23, 58.55, 53.03, 51.15, 46.00.

HRMS *m/z* for $C_9H_{17}N_2O^+$ ([M+H]$^+$) calculated: 169.1335, found: 169.1333.

1-(4-(2-(4-(Prop-2-yn-1-yl)piperazin-1-yl)ethoxy)phenyl)butan-1-one (MS16)

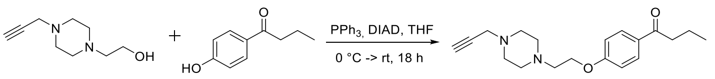

2-(4-(Prop-2-yn-1-yl)piperazin-1-yl)ethan-1-ol (214 mg, 1.27 mmol, 1.0 eq), 1-(4-hydroxyphenyl)butan-1-one (209 mg, 1.27 mmol, 1.0 eq) and triphenylphosphine (PPh_3) (400 mg, 1.53 mmol, 1.2 eq) were dissolved in anhydrous THF (5 mL) under argon atmosphere. The mixture was cooled to 0 °C. Diisopropyl azodicarboxylate (DIAD) (400 µL, 2.04 mmol, 1.6 eq) was dissolved in anhydrous THF (7.5 mL) and added dropwise to the reaction mixture. The solution was stirred for 10 min at 0 °C. The ice bath was removed, and the reaction was allowed to stir for 18 h at room temperature. The solvent was evaporated under reduced pressure. The resulting residue was washed with brine (1x20 mL). The aqueous phase was extracted with EA (3x20 mL). The crude product was purified by column chromatography (0-40% EA in PE and 0-10% MeOH in DCM) to obtain MS16 (279 mg, 0.89 mmol, 70%) as a white solid.

^{1}**H NMR** (400 MHz, $CDCl_3$) δ (ppm) = 7.95 (d, *J* = 8.9 Hz, 2H), 6.92 (d, *J* = 8.9 Hz, 2H), 4.50-4.41 (m, 2H), 3.72 (d, *J* = 2.6 Hz, 2H), 3.69-3.39 (m, 10H), 2.90 (t, *J* = 7.3 Hz, 2H), 2.59 (t, *J* = 2.5 Hz, 1H), 1.75 (h, *J* = 7.4 Hz, 2H), 1.00 (t, *J* = 7.4 Hz, 3H).

^{13}**C NMR** (101 MHz, $CDCl_3$) δ (ppm) = 199.23, 160.64, 131.70, 130.65, 114.30, 78.85, 72.46, 62.88, 56.15, 50.75, 48.09, 46.03, 40.45, 18.06, 14.03.

HRMS *m/z* for $C_{19}H_{27}N_2O_2^+$ ([M+H]$^+$) calculated: 315.2067, found: 315.2069.

1-(4-(2-(4-(Prop-2-yn-1-yl)piperazin-1-yl)ethoxy)phenyl)butan-1-amine (MS24)

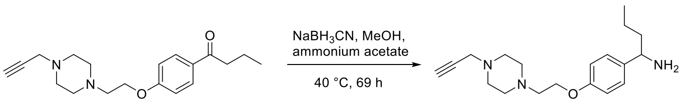

1-(4-(2-(4-(Prop-2-yn-1-yl)piperazin-1-yl)ethoxy)phenyl)butan-1-one (588 mg, 1.87 mmol, 1.0 eq) was dissolved in MeOH (20 mL) and ammonium acetate (4.325 g, 56.10 mmol, 30 eq) was added. The flask was charged with argon and a 1 M solution of $NaBH_3CN$ in dry THF (9 mL, 9.35 mmol, 5.0 eq) was added dropwise to the reaction mixture at room temperature. The mixture was heated to 40 °C and allowed to stir for 69 h. Thereafter the reaction was cooled to room temperature and diluted with EA (30 mL). The mixture was then washed with sat. $NaHCO_3$ solution (2 x 20 mL) and brine (1 x 20 mL). The aqueous phases were combined, and the pH was adjusted to 10 using aq. 2 M NaOH solution. Then the aqueous phase was extracted with EA (1 x 30 mL). The organic phases were combined, dried over $MgSO_4$ and the solvent was evaporated under reduced pressure. The crude product was isolated as a sticky yellow oil and was used for the next reaction without further purification.

^{1}**H NMR** (500 MHz, DMSO-*d*$_6$) δ (ppm) = 8.07 (s, 2H), 7.35 (d, *J* = 8.7 Hz, 2H), 7.00 (d, *J* = 8.8 Hz, 2H), 4.16 (dd, *J* = 9.4, 5.6 Hz, 1H), 4.07 (t, *J* = 5.8 Hz, 2H), 4.03 (q, *J* = 7.1 Hz, 2H), 3.24 (d, *J* = 2.4 Hz, 2H), 3.15 (t, *J* = 2.4 Hz, 1H), 2.68 (t, *J* = 5.4 Hz, 4H), 2.45 (s, 4H), 1.84-1.67 (m, 2H), 1.23 (s, 2H), 0.84 (t, *J* = 7.4 Hz, 3H).

^{13}**C NMR** (126 MHz, DMSO-*d*$_6$) δ (ppm) = 158.65, 131.55, 128.75, 114.70, 79.43, 75.72, 65.62, 59.79, 56.49, 53.65, 52.94, 45.99, 40.11, 39.85, 18.44, 14.11.

LC-MS *m/z* for $C_{19}H_{30}N_3O^+$ ([M+H]$^+$) calculated: 316.2383, found: 316.2385.

2-Cyano-*N*-(1-(4-(2-(4-(prop-2-yn-1-yl)piperazin-1-yl)ethoxy)phenyl)butyl)-acetamide (MS35)

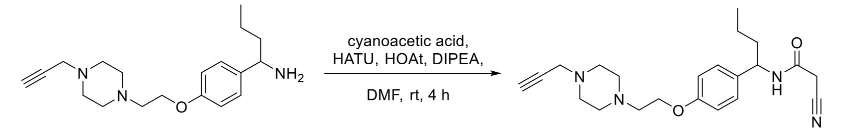

Cyanoacetic acid (95 mg, 1.12 mmol, 1.0 eq) was dissolved in dry DMF (2 mL) to which HATU (425 mg, 1.12 mmol, 1.0 eq) and HOAt (152 mg, 1.12 mmol, 1.0 eq) were added. The mixture was stirred for 30 min at room temperature, then cooled to 0 °C. 1-(4-(2-(4-(Prop-2-yn-1-yl)piperazin-1-yl)ethoxy)phenyl)butan-1-amine (353 mg, 1.12 mmol, 1 eq) was dissolved in dry DMF (2 mL) and added dropwise to the ice cold reaction mixture, followed by addition of DIPEA (419 µL, 2.46 mmol, 2.2 eq). The ice bath was removed, and the reaction was allowed to stir for 4 h at room temperature. Afterwards the reaction mixture was diluted with EA (10 mL) and washed with sat. $NaHCO_3$ solution (2x10 mL) and brine (1x10 mL). The organic phase was dried over $MgSO_4$ and the solvent was evaporated under reduced pressure. The crude product was purified by column chromatography (0-15% MeOH in DCM) to yield MS35 (68 mg, 0.18 mmol, 15% over two steps) as a sticky yellow oil.

^{1}H NMR (500 MHz, DMSO-*d*$_6$) δ (ppm) = 8.58 (d, *J* = 8.3 Hz, 1H), 7.19 (d, *J* = 8.7 Hz, 2H), 6.89 (d, *J* = 8.7 Hz, 2H), 4.69 (q, *J* = 8.2 Hz, 1H), 4.07 (t, 2H), 3.71-3.57 (m, 2H), 3.27 (s, 2H), 3.18 (s, 1H), 2.63 (dt, *J* = 3.6, 1.8 Hz, 2H), 2.53-2.45 (m, 8H), 1.60 (dddd, *J* = 22.5, 15.5, 8.7, 5.0 Hz, 2H), 1.25 (ddd, *J* = 19.6, 15.4, 7.0 Hz, 2H), 0.85 (t, *J* = 7.4 Hz, 3H).

^{13}C NMR (126 MHz, DMSO-*d*$_6$) δ (ppm) = 161.26, 157.25, 135.16, 127.57, 116.31, 114.30, 75.90, 54.95, 52.72, 52.30, 45.86, 38.23, 25.37, 19.08, 13.59.

HRMS *m/z* for $C_{22}H_{31}N_4O_2^+$ ($[M+H]^+$) calculated: 383.2442, found: 383.2443.

(*E*)-3-(6-Bromopyridin-2-yl)-2-cyano-N-(1-(4-(2-(4-(prop-2-yn-1-yl)piperazin-1-yl)ethoxy)phenyl)butyl)acrylamide (MS37)

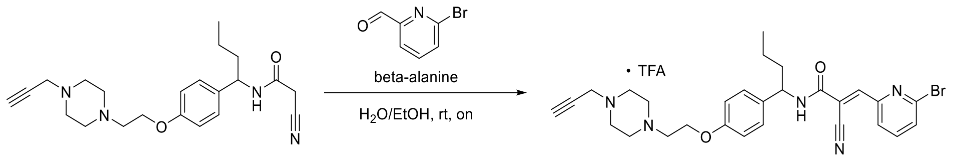

2-Cyano-*N*-(1-(4-(2-(4-(prop-2-yn-1-yl)piperazin-1-yl)ethoxy)phenyl)butyl)acetamide (58 mg, 0.15 mmol, 1.0 eq) was dissolved in EtOH (99%, absolute). Beta-alanine (203 mg, 2.27 mmol, 15 eq) and ddH_2O (2 mL) were added, followed by 6-bromo-2-pyridinecarboxaldehyde (113 mg, 0.61 mmol, 4 eq). The reaction mixture was stirred for 18 h at room temperature. Afterwards the reaction mixture was diluted with EA (6 mL) and washed with sat. $NaHCO_3$ solution (2x10 mL) and brine (1x10 mL). The organic phase was dried over $MgSO_4$ and the solvent was evaporated under reduced pressure. The crude product was isolated as a crystalline yellow solid. The crude material was further purified by preparative HPLC (gradient B) to obtain MS37 (40 mg, 0.07 mmol, 48%) as a pale-yellow TFA salt.

^{1}H NMR (500 MHz, $CDCl_3$) δ (ppm) = 8.15 (s, 1H), 7.70 – 7.62 (m, 1H), 7.61 – 7.52 (m, 2H), 7.26 (d, *J* = 8.6 Hz, 2H), 6.89 – 6.81 (m, 2H), 4.98 (q, *J* = 7.5 Hz, 1H), 4.40 – 4.22 (m, 2H), 3.81 – 3.74 (m, 2H), 3.70 (s, 4H), 3.57 – 3.47 (m, 6H), 2.62 (s, 1H), 1.94 – 1.76 (m, 2H), 1.44 – 1.17 (m, 2H), 0.94 (t, *J* = 7.4 Hz, 3H).

^{13}C NMR (126 MHz, $CDCl_3$) δ (ppm) = 162.00 (q, *J* = 38.3 Hz), 158.97, 156.49, 150.88, 148.50, 142.51, 139.35, 135.39, 130.87, 128.20, 125.81, 115.85, 115.79 (d, *J* = 288.0 Hz), 114.82, 109.35, 79.45, 71.69, 62.49, 56.15, 54.26, 50.27, 47.81, 45.89, 38.02, 19.52, 13.80.

HRMS *m/z* for $C_{28}H_{33}BrN_5O_2^+$ ($[M+H]^+$) calculated: 550.1812, found: 550.1823.

3.7 Synthesis of compound MS23

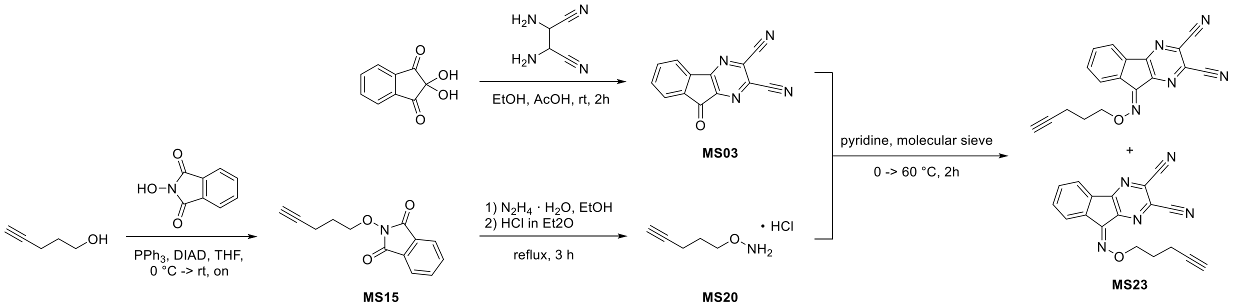

Scheme 3.7: Synthesis of compound MS23 based on [32].

9-Oxo-9*H*-indeno[1,2-*b*]pyrazine-2,3-dicarbonitrile (MS03)

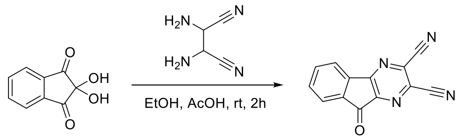

Ninhydrin (100 mg, 0.56 mmol, 1.0 eq) and diaminomaleonitrile (DAMN) (30 mg, 0.5 eq, 0.28 mmol) were dissolved in anhydrous EtOH (5 mL) and acetic acid (0.5 mL) was added. The mixture was stirred for 2 h at room temperature. The precipitated solid was filtered and washed with cold EtOH, to obtain MS03 (30 mg, 0.13 mmol, 23%) as a yellow solid. The product was used for the following reactions without further purification. The analytical characterization was in good agreement with previously reported data[32].

^{1}H NMR (400 MHz, DMSO-d_6) δ (ppm) = 8.11 (d, J = 7.5 Hz, 1H), 8.00-7.89 (m, 2H), 7.82 (td, J = 7.5, 1.1 Hz, 1H).

^{13}C NMR (176 MHz, DMSO-d_6) δ (ppm) = 186.49, 160.75, 150.86, 138.58, 137.83, 136.29, 135.29, 134.57, 132.44, 125.55, 124.11, 114.83.

LC-MS *m/z* for $C_{13}H_5N_4O^+$ ($[M+H]^+$) calculated: 233.04, found: 233.00.

2-(Pent-4-yn-1-yloxy)isoindoline-1,3-dione (MS15)

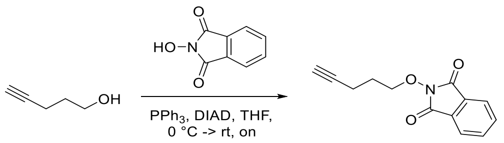

4-Pentyn-1-ol (55 µL, 0.59 mmol, 1.0 eq), *N*-hydroxyphthalimide (116 mg, 0.71 mmol, 1.2 eq) and triphenylphosphine (PPh_3) (203 mg, 0.77 mmol, 1.3 eq) were dissolved in anhydrous THF (2 mL) under argon atmosphere. The mixture was then cooled to 0 °C. Diisopropyl azodicarboxylate (DIAD) (187 µL, 0.95 mmol, 1.6 eq) was dissolved in anhydrous THF (2 mL) and added dropwise. The reaction mixture was stirred for 10 min at 0 °C, until complete dissolution. The ice bath was removed, and the reaction was allowed to stir overnight at room temperature. The solvent was evaporated under reduced pressure. The crude product was purified by column chromatography (0-40% EA in PE) to obtain MS15 (127 mg, 0.55 mmol, 93%) as a pale-yellow solid. The analytical characterization was in good agreement with previously reported data[33].

^{1}H NMR (700 MHz, DMSO-*d*$_6$) δ (ppm) = 7.86 (s, 4H), 4.21 (t, *J* = 6.3 Hz, 2H), 2.81 (t, *J* = 2.7 Hz, 1H), 2.39 (td, *J* = 7.2, 2.6 Hz, 2H), 1.85 (p, *J* = 6.7 Hz, 2H).

^{13}C NMR (176 MHz, DMSO-*d*$_6$) δ (ppm) = 163.34, 134.77, 128.61, 123.23, 83.53, 76.34, 71.63, 26.91, 14.24.

LC-MS *m/z* for $C_{13}H_{12}NO_3^+$ ($[M+H]^+$) calculated: 230.07, found: 230.10.

O-(Pent-4-yn-1-yl)hydroxylamine hydrochloride (MS20)

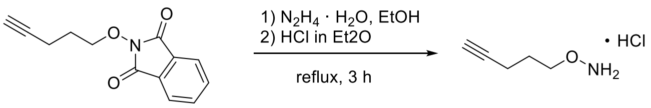

2-(Pent-4-yn-1-yloxy)isoindoline-1,3-dione (300 mg, 1.31 mmol, 1.0 eq) was dissolved in anhydrous EtOH (10 mL). Hydrazine hydrate (63 µL, 1.96 mmol, 1.5 eq) was added, the reaction mixture was heated to reflux and stirred for 3 h. The reaction was quenched by adding a solution of sat. $NaHCO_3$. The aqueous phase was extracted with DCM (3x10 mL). The combined organic layers were washed with brine and dried over Na_2SO_4. The solvent was removed under reduced pressure. The resulting slurry was dissolved in 2 N HCl in Et_2O (2 mL) and the solvent was removed *in vacuo*. The formed precipitate was washed with cold Et_2O and collected by vacuum filtration to yield MS20 (59 mg, 0.60 mmol, 45%) as a white solid. The product was used for the next reaction without further purification. The analytical characterization was in good agreement with previously reported data[33].

^{1}H NMR (500 MHz, DMSO-*d*$_6$) δ (ppm) = 11.05 (s, 2H), 4.07 (t, *J* = 6.4 Hz, 2H), 2.88 (t, *J* = 2.7 Hz, 1H), 2.24 (td, *J* = 7.2, 2.7 Hz, 2H), 1.78 (p, *J* = 6.8 Hz, 2H).

^{13}C NMR (126 MHz, DMSO-*d*$_6$) δ (ppm) = 83.76, 73.16, 72.40, 26.73, 14.70.

LC-MS *m/z* for $C_5H_{10}NO^+$ ($[M+H]^+$) calculated: 100.07, found: 100.07.

9-((Pent-4-yn-1-yloxy)imino)-9*H*-indeno[1,2-*b*]pyrazine-2,3-dicarbonitrile (MS23)

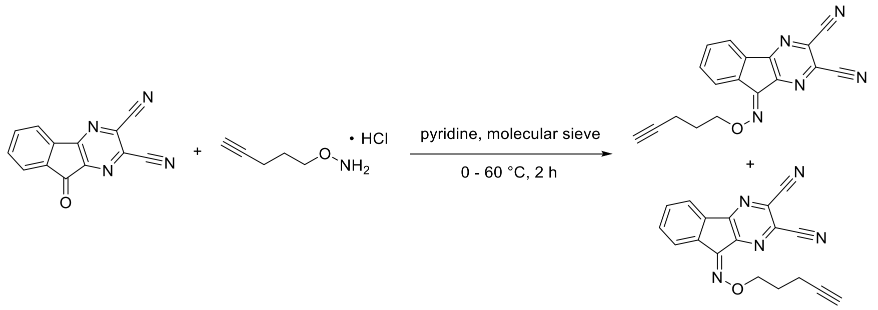

9-Oxo-9H-indeno[1,2-*b*]pyrazine-2,3-dicarbonitrile (30 mg, 0.13 mmol, 1.0 eq) was dissolved in pyridine (5 mL). The mixture was cooled to 0 °C and *O*-(pent-4-yn-1-yl)hydroxylamine hydrochloride (53 mg, 0.39 mmol, 3.0 eq) was added. The reaction mixture was warmed to room temperature and molecular sieves (3 Å) were added. The reaction mixture was heated to 60 °C and stirred for 2 h. Afterwards the mixture was cooled to room temperature and the pyridine was evaporated under reduced pressure. The residue was dissolved in sat. aq. NH_4Cl (10 mL) solution and the aqueous phase was extracted with EA (3x10 mL). The organic layers were combined, dried over Na_2SO_4 and the solvent was evaporated under reduced pressure. The crude product was purified by preparative HPLC (Gradient A) to obtain MS23 (11 mg, 0.04 mmol, 27%, anti/syn-mixture) as a pale-yellow solid.

^{1}H NMR (500 MHz, DMSO-*d*$_6$) δ (ppm) = 8.42 (d, *J* = 7.0 Hz, 1H), 8.22 (d, *J* = 6.9 Hz, 1H), 8.12 (d, *J* = 7.1 Hz, 1H), 7.97 (d, *J* = 7.3 Hz, 1H), 7.92-7.80 (m, 2H), 7.80-7.70 (m, 2H), 4.67 (t, *J* = 6.3 Hz, 2H),

4.61 (t, J = 6.4 Hz, 2H), 2.87 (t, J = 2.6 Hz, 1H), 2.85 (t, J = 2.6 Hz, 1H), 2.43 - 2.37 (m, 2H), 2.37-2.33 (m, 2H), 2.10-2.03 (m, 2H), 2.02-1.96 (m, 2H).

^{13}C NMR (126 MHz, DMSO-d_6) δ (ppm) = 155.70, 155.09, 151.28, 145.93, 145.33, 145.20, 136.72, 134.58, 134.16, 133.77, 133.18, 132.77, 132.38, 132.28, 131.99, 131.68, 131.01, 130.71, 128.79, 123.53, 123.48, 121.83, 114.80, 114.67, 114.54, 83.57, 83.53, 76.37, 75.95, 71.99, 71.86, 39.85, 27.90, 27.69, 14.53, 14.27.

HRMS *m/z* for $C_{18}H_{12}N_5O^+$ ($[M+H]^+$) calculated: 314.1036, found: 314.1034.

4. Chemical characterization data

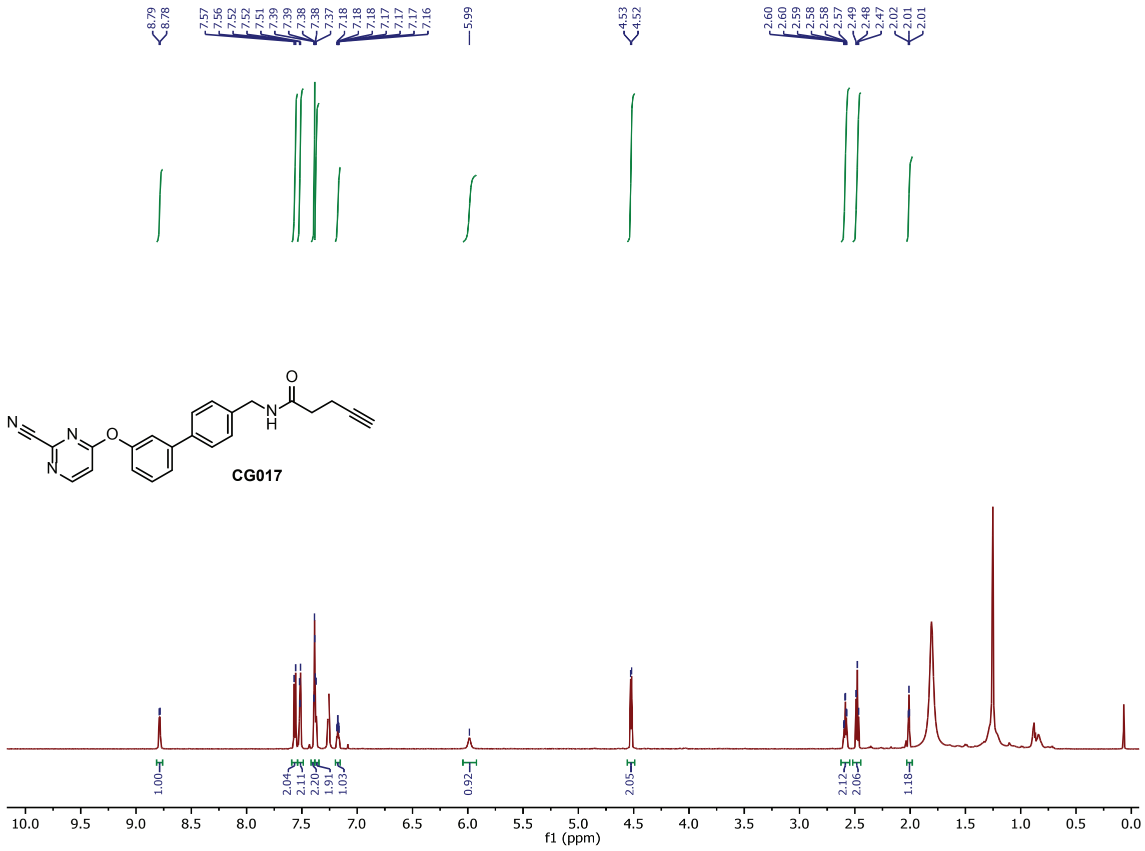
8.79
8.78
7.57
7.56
7.52
7.52
7.51
7.39
7.39
7.38
7.38
7.37
7.18
7.18
7.18
7.17
7.17
7.17
7.16
5.99
4.53
4.52
2.60
2.60
2.59
2.58
2.58
2.57
2.49
2.48
2.47
2.02
2.01
2.01
CG017
1.00
2.04
2.11
2.20
1.91
1.03
0.92
2.05
2.12
2.06
1.18
f1 (ppm)

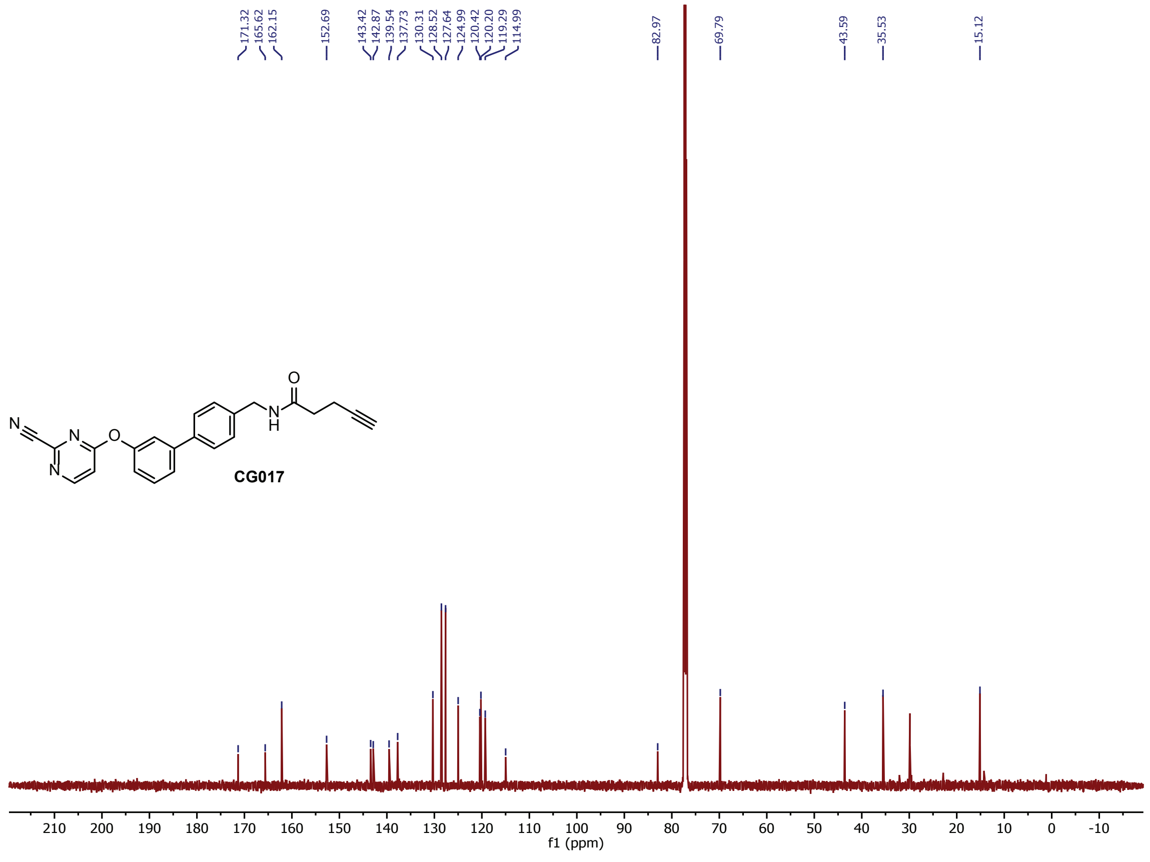
171.32
165.62
162.15
152.69
143.42
142.87
139.54
137.73
130.31
128.52
127.64
124.99
120.42
120.20
119.29
114.99
82.97
69.79
43.59
35.53
15.12
CG017
f1 (ppm)

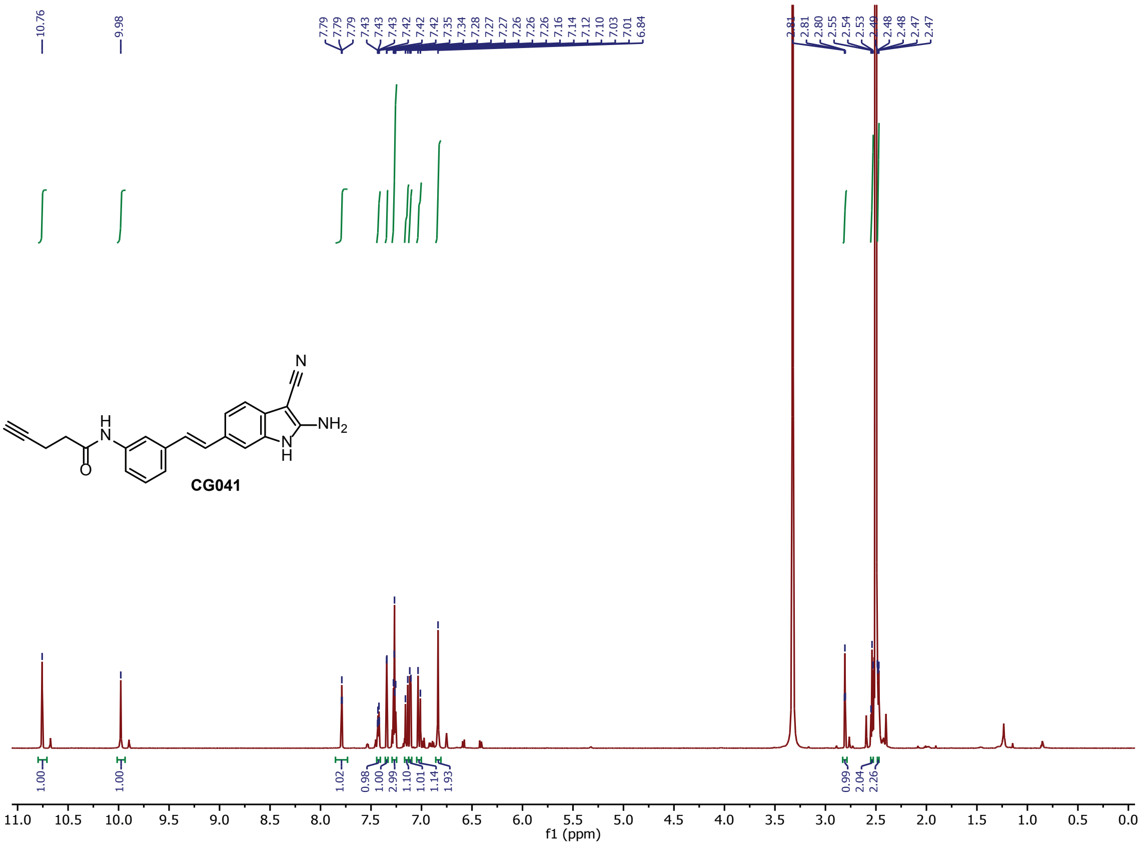
10.76
9.98
7.79 7.79 7.79 7.43 7.43 7.42 7.42 7.42 7.35 7.34 7.28 7.27 7.27 7.26 7.26 7.26 7.16 7.14 7.12 7.10 7.03 7.01 6.84
2.81 2.81 2.80 2.55 2.54 2.53 2.48 2.48 2.47 2.47
N
H
N
NH2
N
H
O
CG041
1.00
1.00
1.02
0.98 1.00 2.99 1.10 1.01 1.14 1.93
0.99 2.04 2.26
11.0 10.5 10.0 9.5 9.0 8.5 8.0 7.5 7.0 6.5 6.0 5.5 5.0 4.5 4.0 3.5 3.0 2.5 2.0 1.5 1.0 0.5 0.0
f1 (ppm)

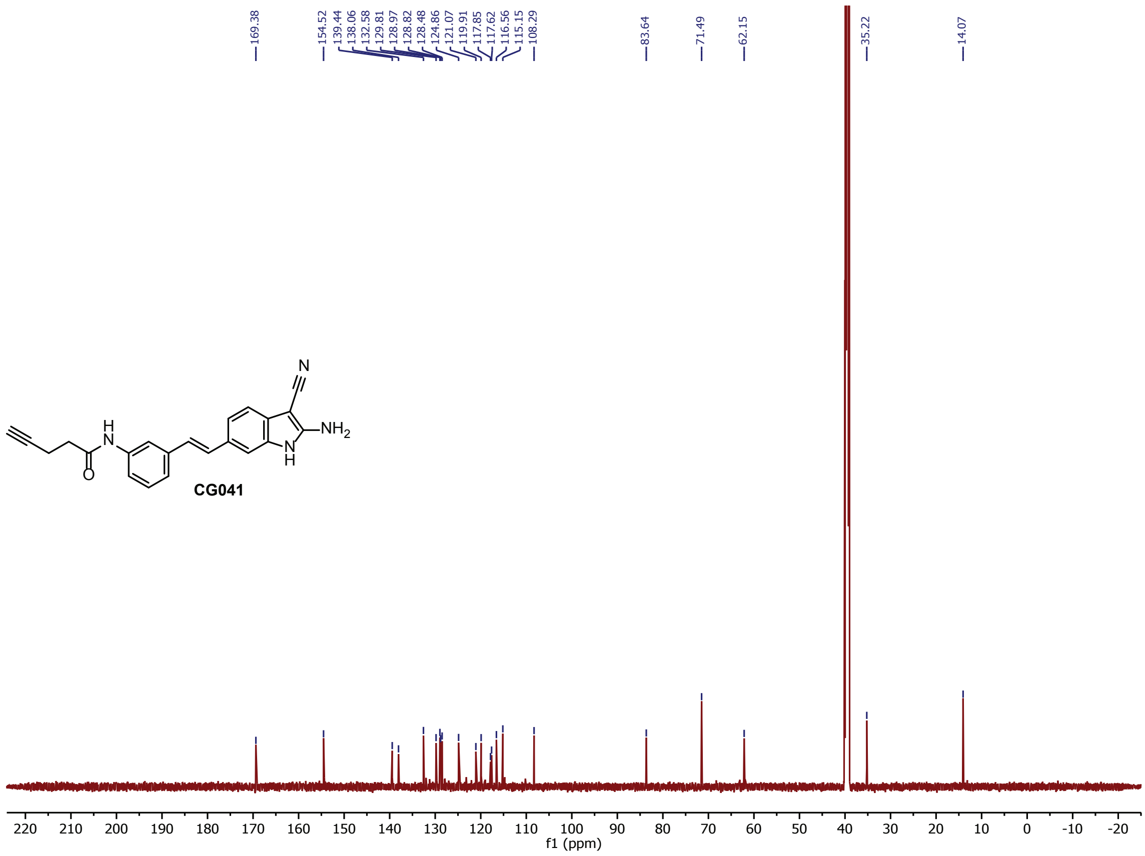
169.38
154.52 139.44 138.06 132.58 129.81 128.97 128.82 128.48 124.86 121.07 119.91 117.85 117.62 116.56 115.15 108.29
83.64
71.49
62.15
35.22
14.07
N
H
N
NH2
N
H
O
CG041
220 210 200 190 180 170 160 150 140 130 120 110 100 90 80 70 60 50 40 30 20 10 0 -10 -20
f1 (ppm)

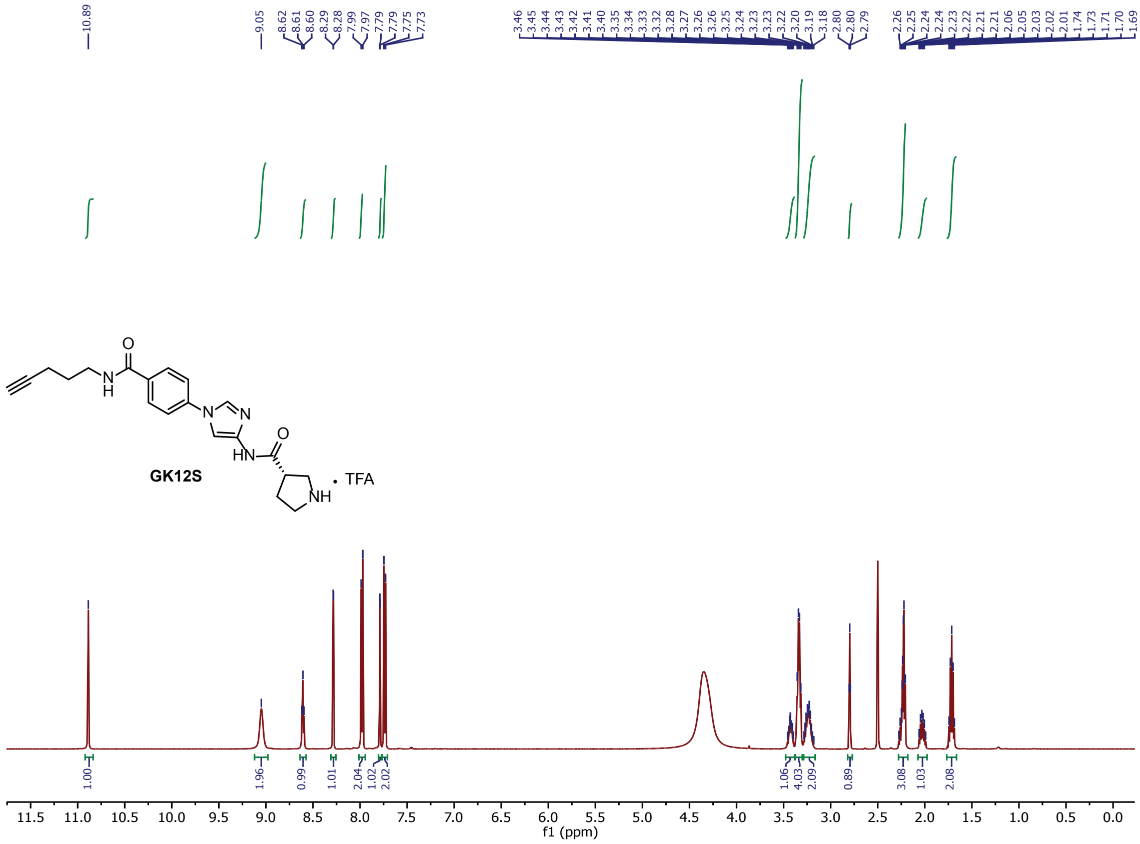

10.89
9.05
8.62
8.61
8.60
8.29
8.28
7.99
7.97
7.79
7.79
7.75
7.73
3.46
3.45
3.44
3.43
3.42
3.41
3.40
3.35
3.34
3.33
3.32
3.28
3.27
3.26
3.26
3.25
3.24
3.23
3.23
3.22
3.20
3.19
3.18
2.80
2.80
2.79
2.26
2.25
2.24
2.24
2.23
2.22
2.21
2.21
2.06
2.05
2.03
2.02
2.01
1.74
1.73
1.71
1.70
1.69
GK12S
TFA
1.00
1.96
0.99
1.01
2.04
1.02
2.02
1.06
4.03
2.09
0.89
3.08
1.03
2.08
f1 (ppm)

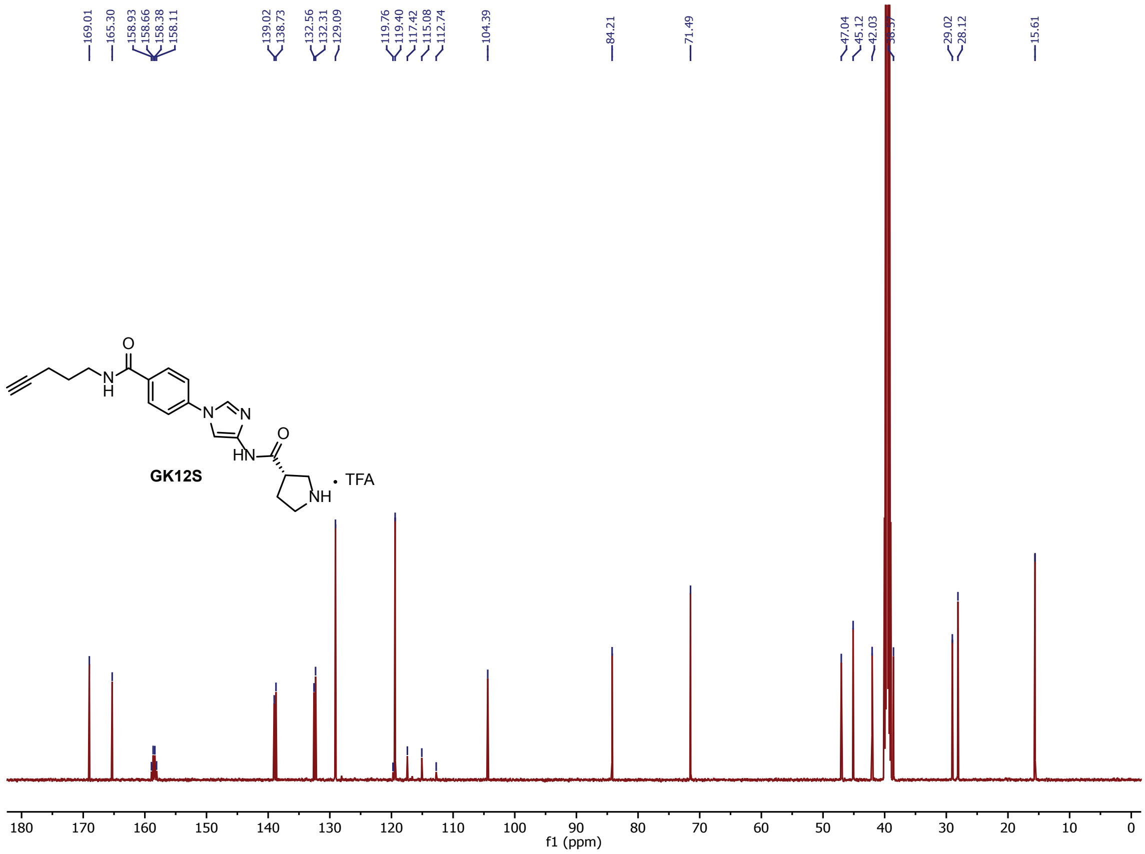

169.01
165.30
158.93
158.66
158.38
158.11
139.02
138.73
132.56
132.31
129.09
119.76
119.40
117.42
115.08
112.74
104.39
84.21
71.49
47.04
45.12
42.03
29.02
28.12
15.61
GK12S
TFA
f1 (ppm)

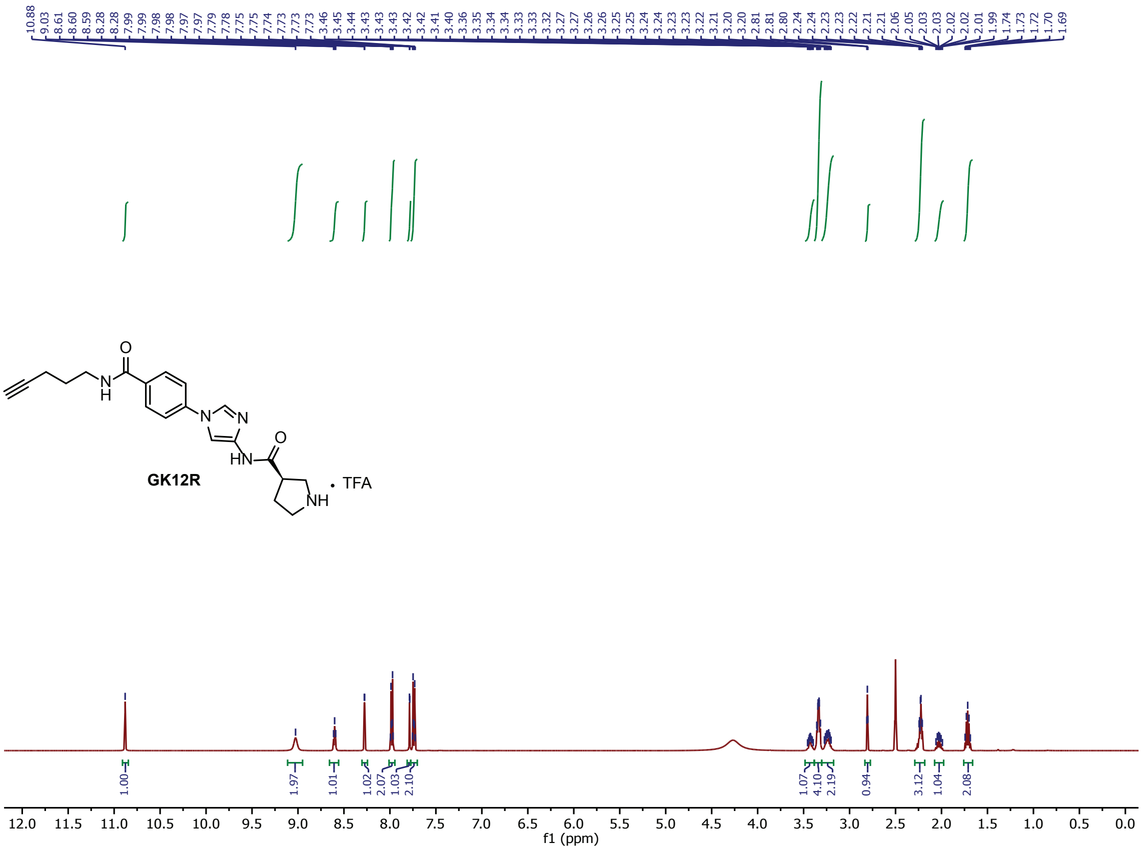
GK12R
• TFA
f1 (ppm)

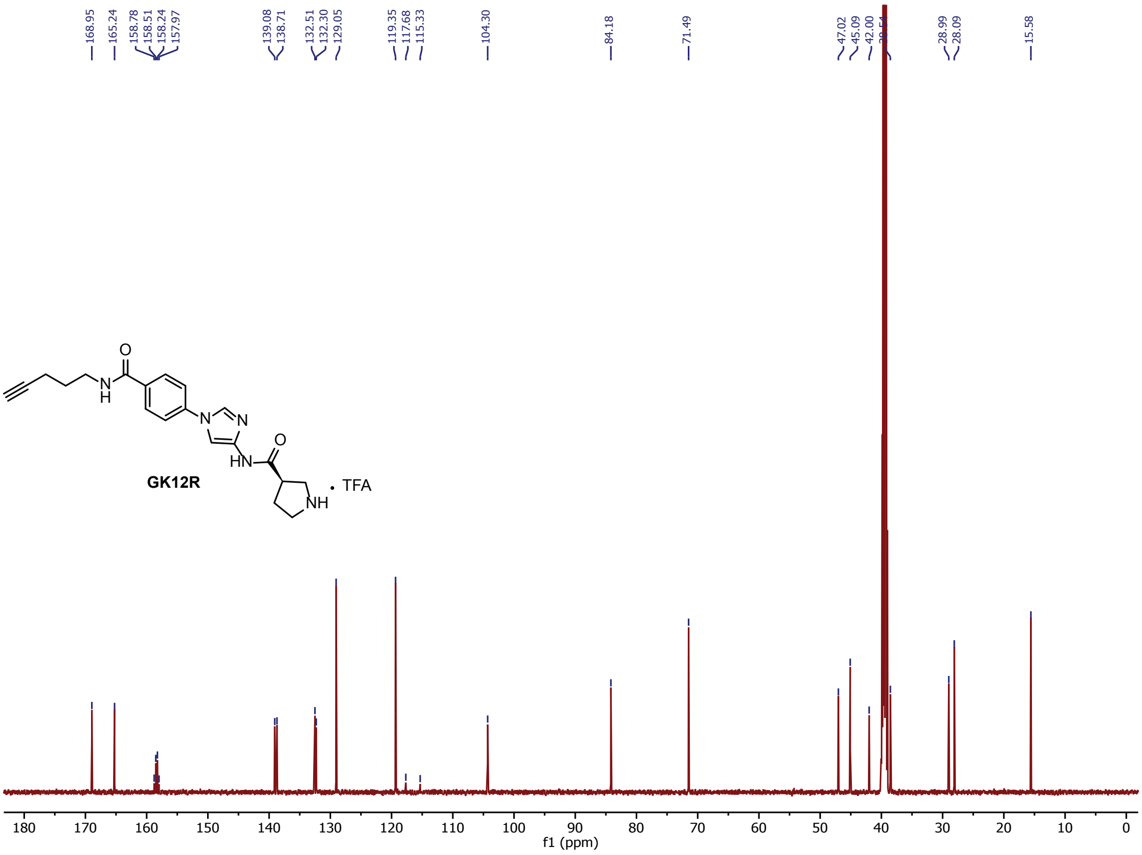
GK12R
• TFA
f1 (ppm)

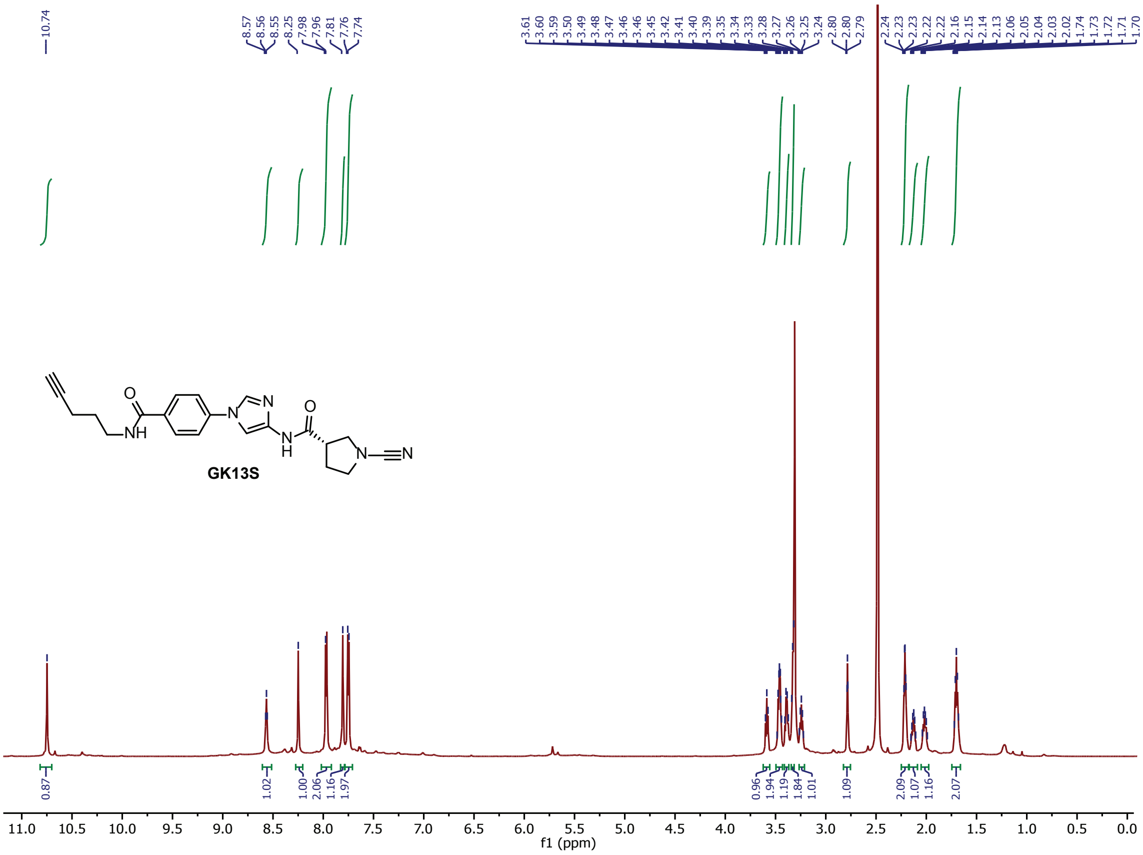

10.74
8.57 8.56 8.55 8.25 7.98 7.96 7.81 7.76 7.74
3.61 3.60 3.59 3.50 3.49 3.48 3.47 3.46 3.45 3.42 3.41 3.40 3.39 3.35 3.34 3.33 3.28 3.27 3.26 3.25 3.24 2.80 2.80 2.79
2.24 2.23 2.23 2.22 2.22 2.16 2.15 2.14 2.13 2.06 2.05 2.04 2.03 2.02 1.74 1.73 1.72 1.71 1.70
0.87 1.02 1.00 2.06 1.16 1.97 0.96 1.94 1.19 1.84 1.01 1.09 2.09 1.07 1.16 2.07
O
NH
N
N
O
N
H
N
N
GK13S
f1 (ppm)

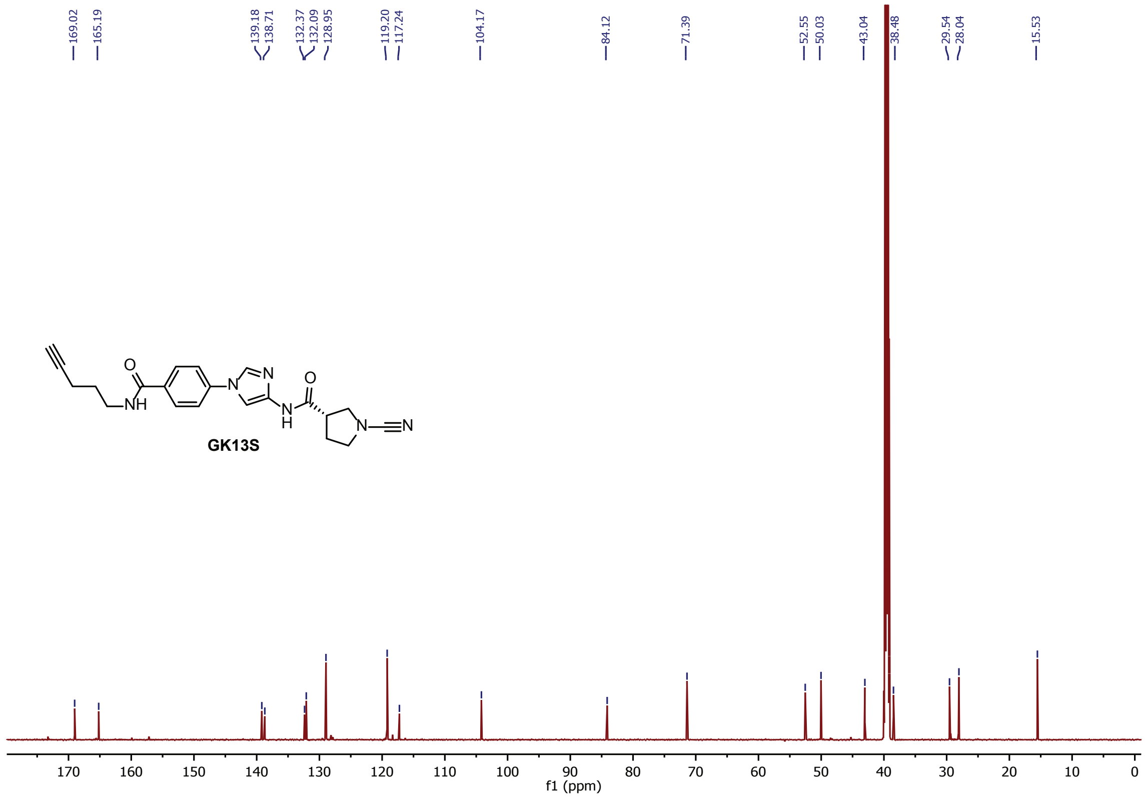

169.02 165.19 139.18 138.71 132.37 132.09 128.95 119.20 117.24 104.17 84.12 71.39 52.55 50.03 43.04 38.48 29.54 28.04 15.53
O
NH
N
N
O
N
H
N
N
GK13S
f1 (ppm)

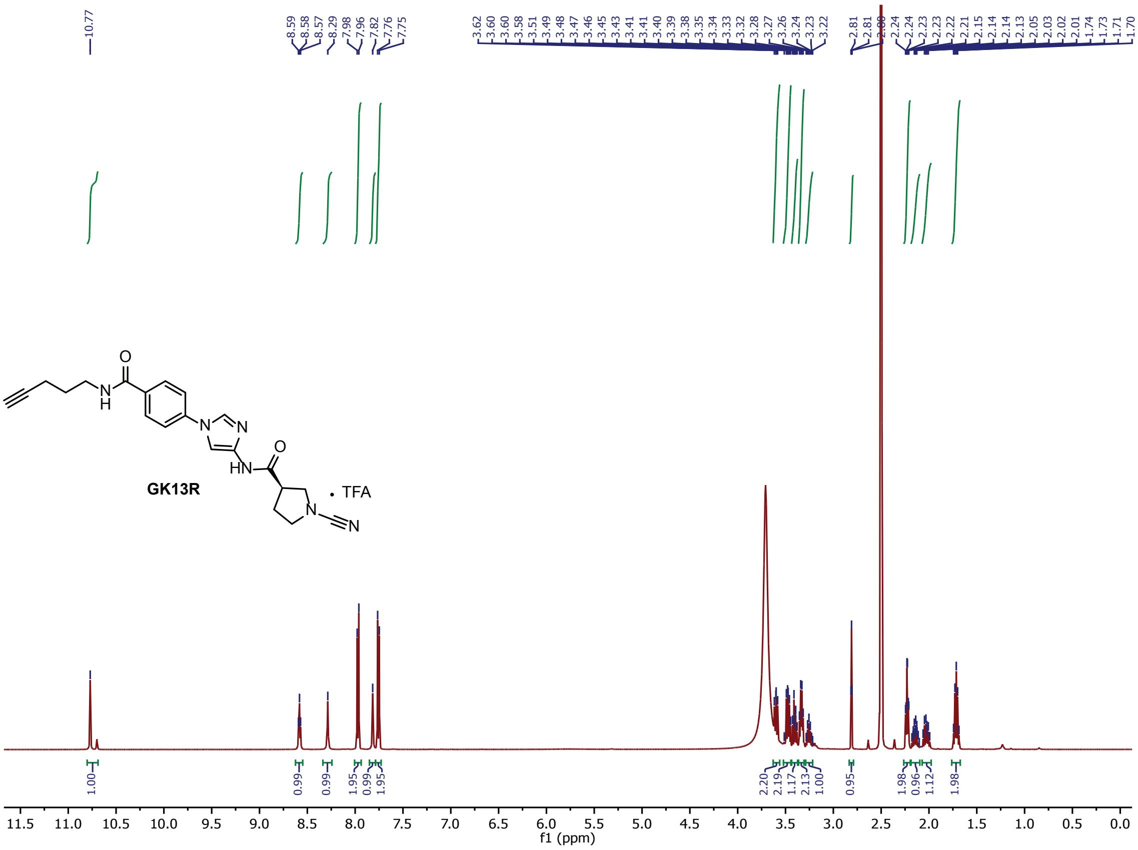
10.77
8.59
8.58
8.57
8.29
7.98
7.96
7.82
7.76
7.75
3.62 3.60 3.60 3.58 3.51 3.49 3.48 3.47 3.46 3.45 3.43 3.41 3.41 3.40 3.39 3.38 3.35 3.34 3.33 3.32 3.28 3.27 3.26 3.24 3.23 3.22
2.81
2.81
2.24 2.24 2.23 2.23 2.22 2.21 2.15 2.14 2.13 2.05 2.03 2.01 1.74 1.73 1.71 1.70
GK13R
• TFA
1.00
0.99
0.99
1.95
0.99
1.95
2.20
2.19
1.17
2.13
1.00
0.95
1.98
0.96
1.12
1.98
11.5 11.0 10.5 10.0 9.5 9.0 8.5 8.0 7.5 7.0 6.5 6.0 5.5 5.0 4.5 4.0 3.5 3.0 2.5 2.0 1.5 1.0 0.5 0.0
f1 (ppm)

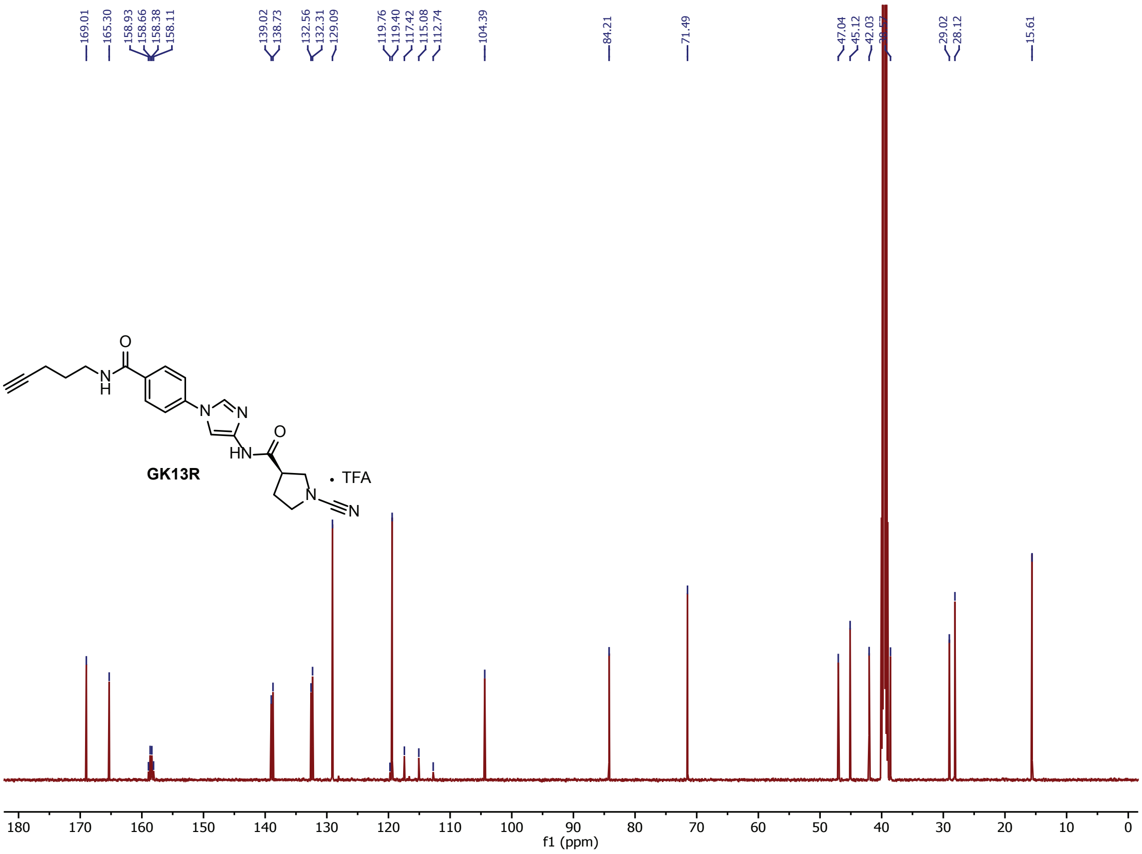
169.01
165.30
158.93
158.66
158.38
158.11
139.02
138.73
132.56
132.31
129.09
119.76
119.40
117.42
115.08
112.74
104.39
84.21
71.49
47.04
45.12
42.03
29.02
28.12
15.61
GK13R
• TFA
180 170 160 150 140 130 120 110 100 90 80 70 60 50 40 30 20 10 0
f1 (ppm)

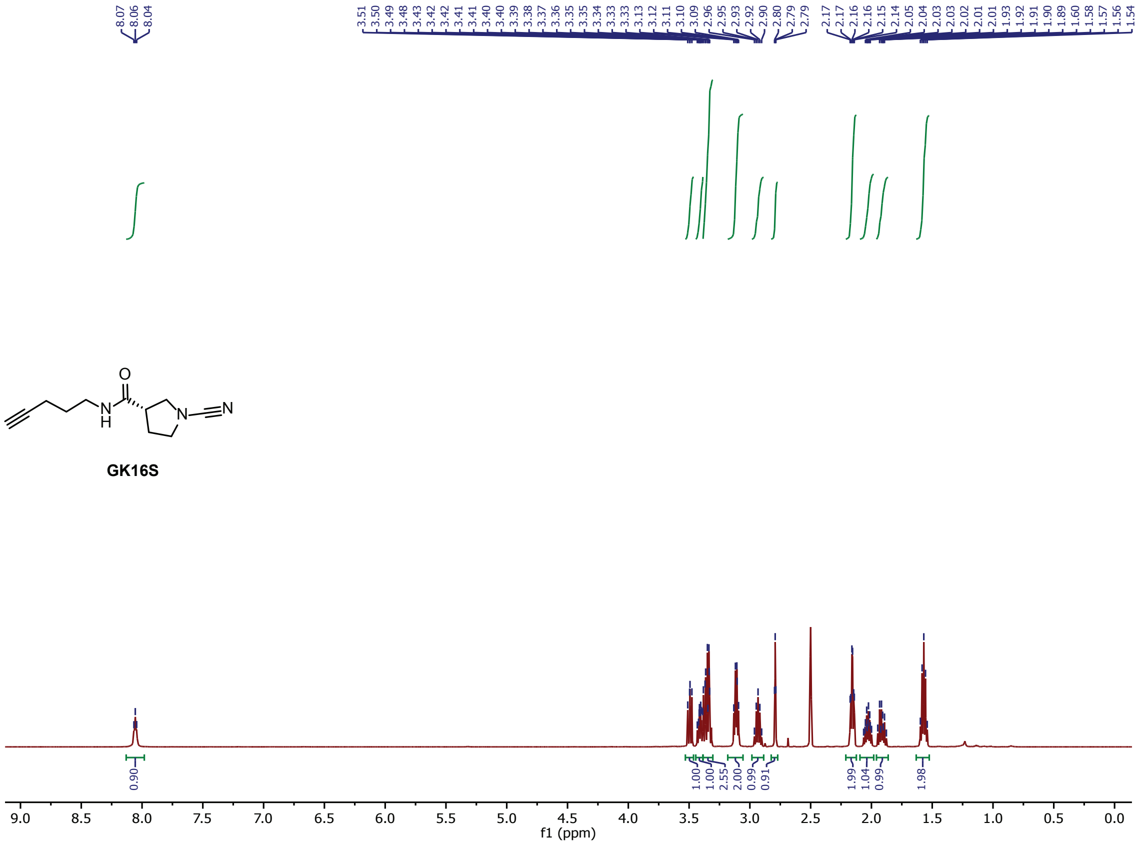
8.07
8.06
8.04
3.51 3.50 3.49 3.48 3.43 3.42 3.41 3.41 3.40 3.39 3.38 3.37 3.36 3.35 3.35 3.34 3.33 3.33 3.13 3.12 3.11 3.10 3.09 2.96 2.95 2.93 2.92 2.90 2.80 2.79 2.79
2.17 2.17 2.16 2.16 2.15 2.14 2.05 2.04 2.03 2.03 2.02 2.01 1.93 1.92 1.91 1.90 1.89 1.60 1.58 1.57 1.56 1.54
O
N
H
N
N
GK16S
0.90
1.00 1.00 2.55 2.00 0.99 0.91
1.99 1.04 0.99
1.98
9.0 8.5 8.0 7.5 7.0 6.5 6.0 5.5 5.0 4.5 4.0 3.5 3.0 2.5 2.0 1.5 1.0 0.5 0.0
f1 (ppm)

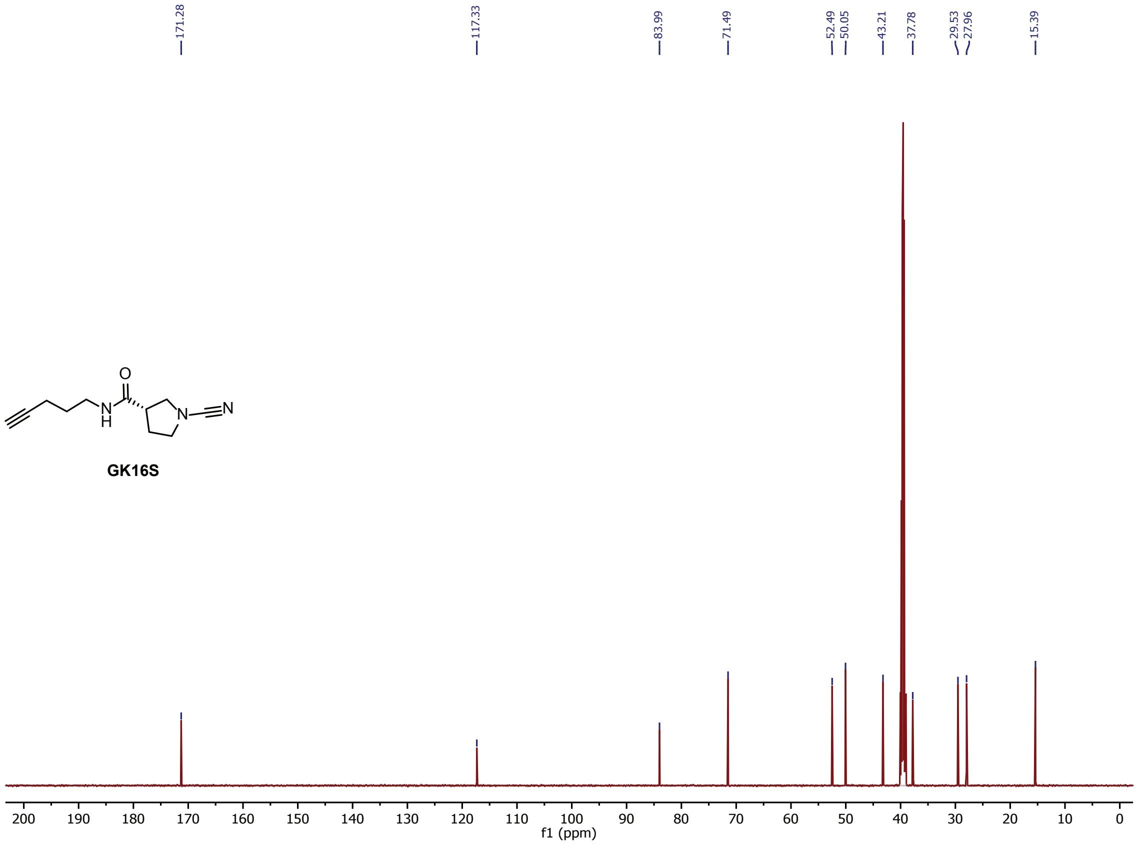
171.28
117.33
83.99
71.49
52.49
50.05
43.21
37.78
29.53
27.96
15.39
O
N
H
N
N
GK16S
200 190 180 170 160 150 140 130 120 110 100 90 80 70 60 50 40 30 20 10 0
f1 (ppm)

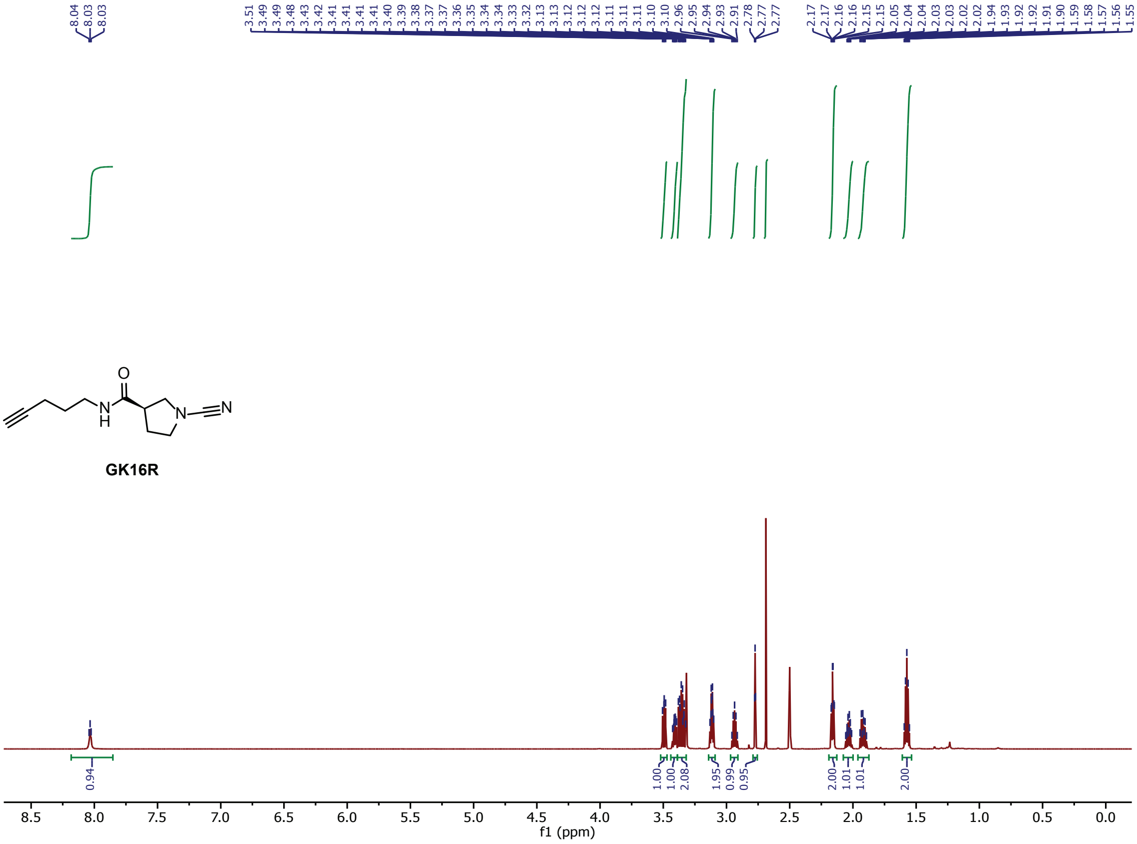

8.04 8.03 8.03
3.51 3.49 3.49 3.48 3.43 3.42 3.41 3.41 3.41 3.40 3.39 3.38 3.37 3.37 3.36 3.35 3.34 3.34 3.33 3.32 3.13 3.13 3.12 3.12 3.11 3.11 3.11 3.10 3.10 2.96 2.95 2.94 2.93 2.91 2.78 2.77 2.77
2.17 2.17 2.16 2.16 2.15 2.05 2.04 2.04 2.03 2.03 2.02 2.02 1.94 1.93 1.92 1.92 1.91 1.90 1.59 1.58 1.57 1.56 1.55
O
N
H
N
N
GK16R
0.94
1.00 1.00 2.08 1.95 0.99 0.95
2.00 1.01 1.01 2.00
8.5 8.0 7.5 7.0 6.5 6.0 5.5 5.0 4.5 4.0 3.5 3.0 2.5 2.0 1.5 1.0 0.5 0.0
f1 (ppm)

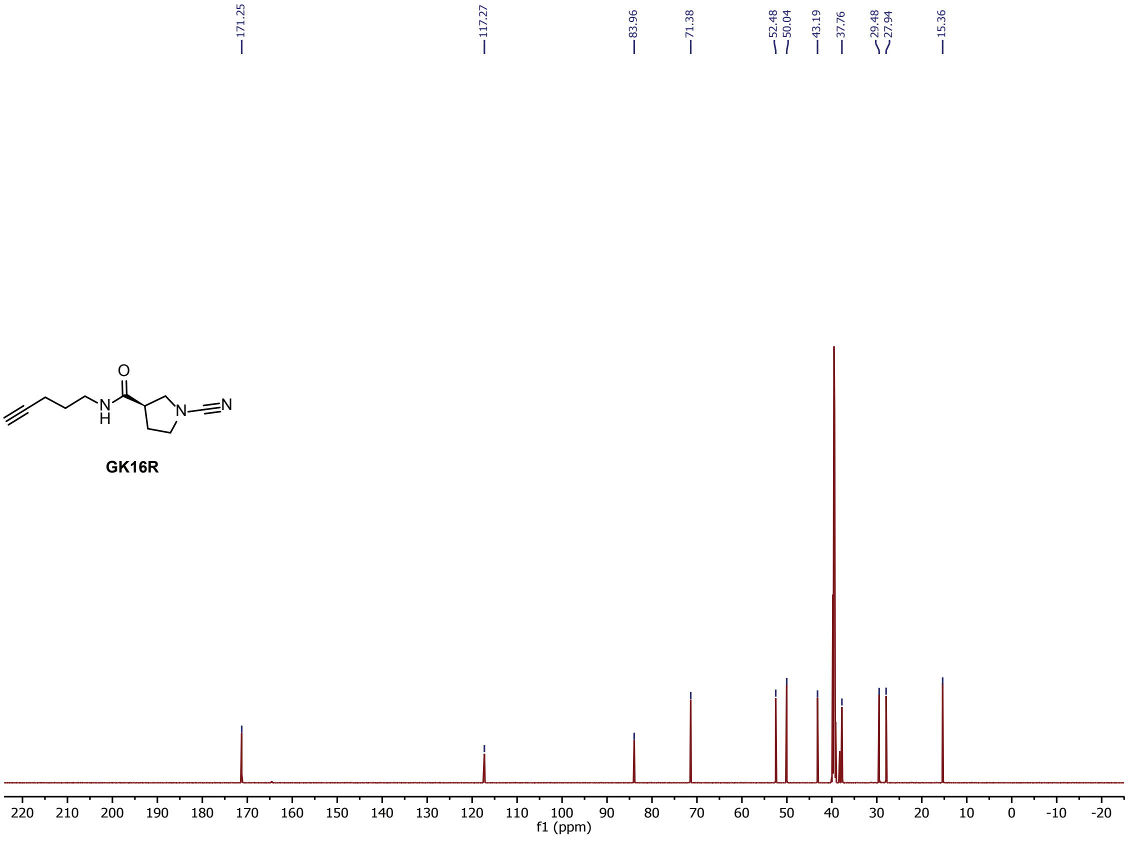

171.25
117.27
83.96
71.38
52.48 50.04
43.19
37.76
29.48 27.94
15.36
O
N
H
N
N
GK16R
220 210 200 190 180 170 160 150 140 130 120 110 100 90 80 70 60 50 40 30 20 10 0 -10 -20
f1 (ppm)

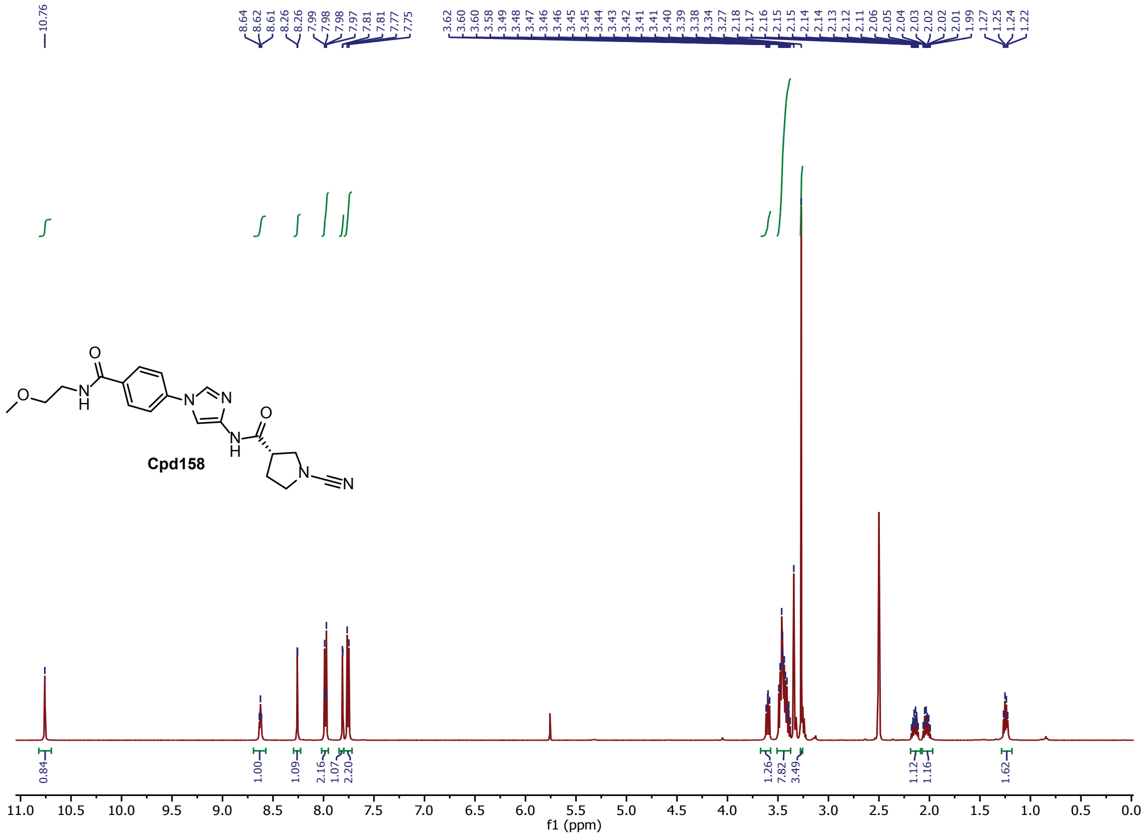
10.76
8.64
8.62
8.61
8.26
8.26
7.99
7.98
7.97
7.81
7.81
7.77
7.75
3.62
3.60
3.60
3.58
3.49
3.48
3.47
3.46
3.45
3.45
3.44
3.43
3.42
3.41
3.41
3.40
3.39
3.38
3.34
3.27
2.18
2.17
2.16
2.15
2.15
2.14
2.13
2.12
2.11
2.06
2.05
2.04
2.03
2.02
2.02
2.01
1.99
1.27
1.25
1.24
1.22
Cpd158
0.84
1.00
1.09
2.16
1.07
2.20
1.26
7.82
3.49
1.12
1.16
1.62
11.0 10.5 10.0 9.5 9.0 8.5 8.0 7.5 7.0 6.5 6.0 5.5 5.0 4.5 4.0 3.5 3.0 2.5 2.0 1.5 1.0 0.5 0.0
f1 (ppm)

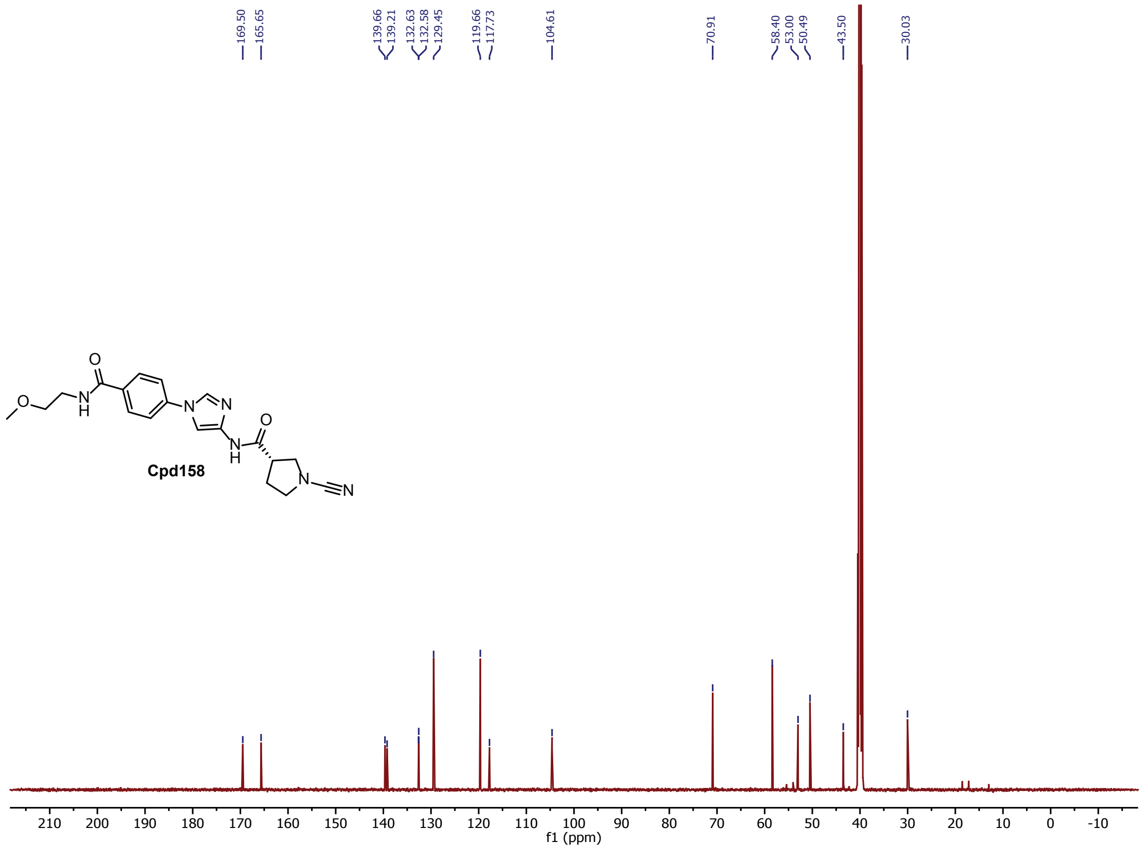
169.50
165.65
139.66
139.21
132.63
132.58
129.45
119.66
117.73
104.61
70.91
58.40
53.00
50.49
43.50
30.03
Cpd158
210 200 190 180 170 160 150 140 130 120 110 100 90 80 70 60 50 40 30 20 10 0 -10
f1 (ppm)

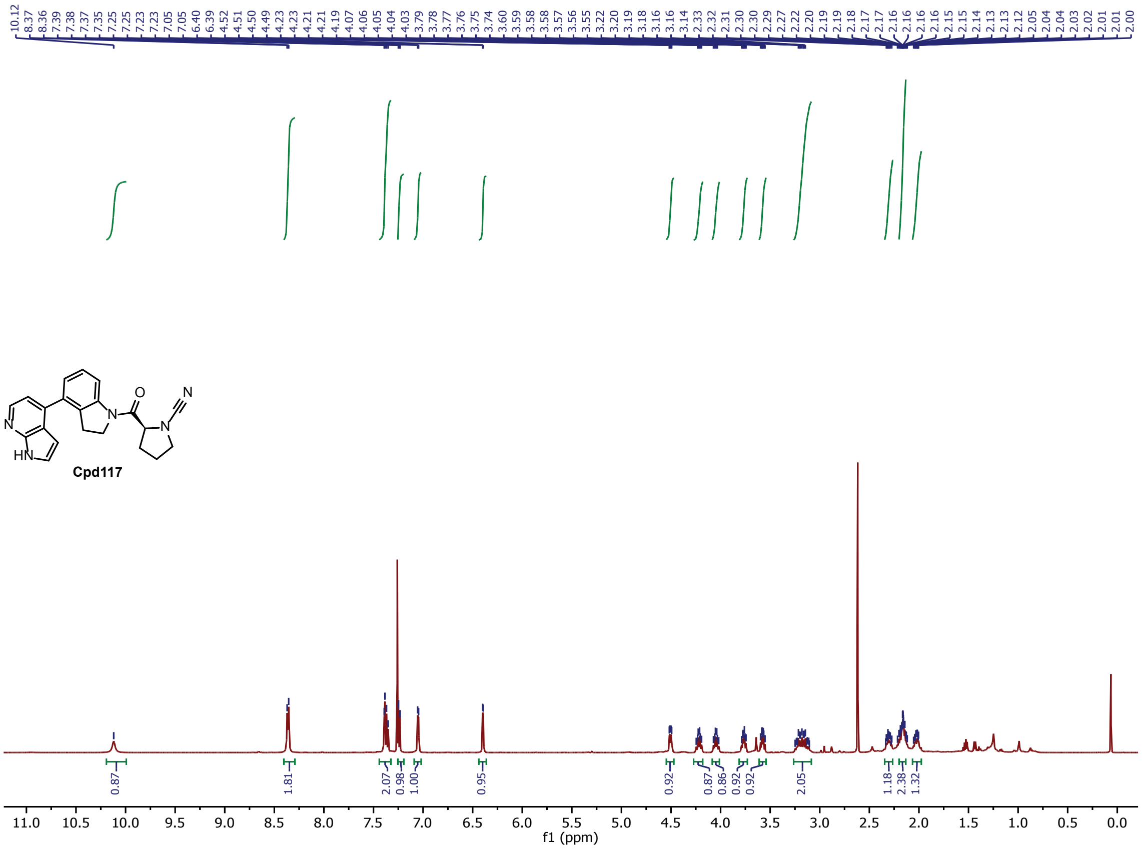

10.12
8.37
8.36
7.39
7.38
7.37
7.35
7.25
7.25
7.23
7.23
7.05
7.05
6.40
6.39
4.52
4.51
4.50
4.49
4.23
4.23
4.21
4.19
4.07
4.06
4.05
4.04
4.03
3.79
3.78
3.77
3.76
3.75
3.74
3.60
3.59
3.58
3.57
3.56
3.55
3.22
3.20
3.19
3.18
3.16
3.16
3.14
2.33
2.32
2.31
2.30
2.30
2.29
2.27
2.22
2.20
2.19
2.19
2.18
2.17
2.17
2.16
2.16
2.16
2.15
2.15
2.14
2.13
2.13
2.12
2.05
2.04
2.04
2.03
2.02
2.01
2.00
O
N
N
N
N
HN
Cpd117
0.87
1.81
2.07
0.98
1.00
0.95
0.92
0.87
0.86
0.92
0.92
2.05
1.18
2.38
1.32
11.0 10.5 10.0 9.5 9.0 8.5 8.0 7.5 7.0 6.5 6.0 5.5 5.0 4.5 4.0 3.5 3.0 2.5 2.0 1.5 1.0 0.5 0.0
f1 (ppm)

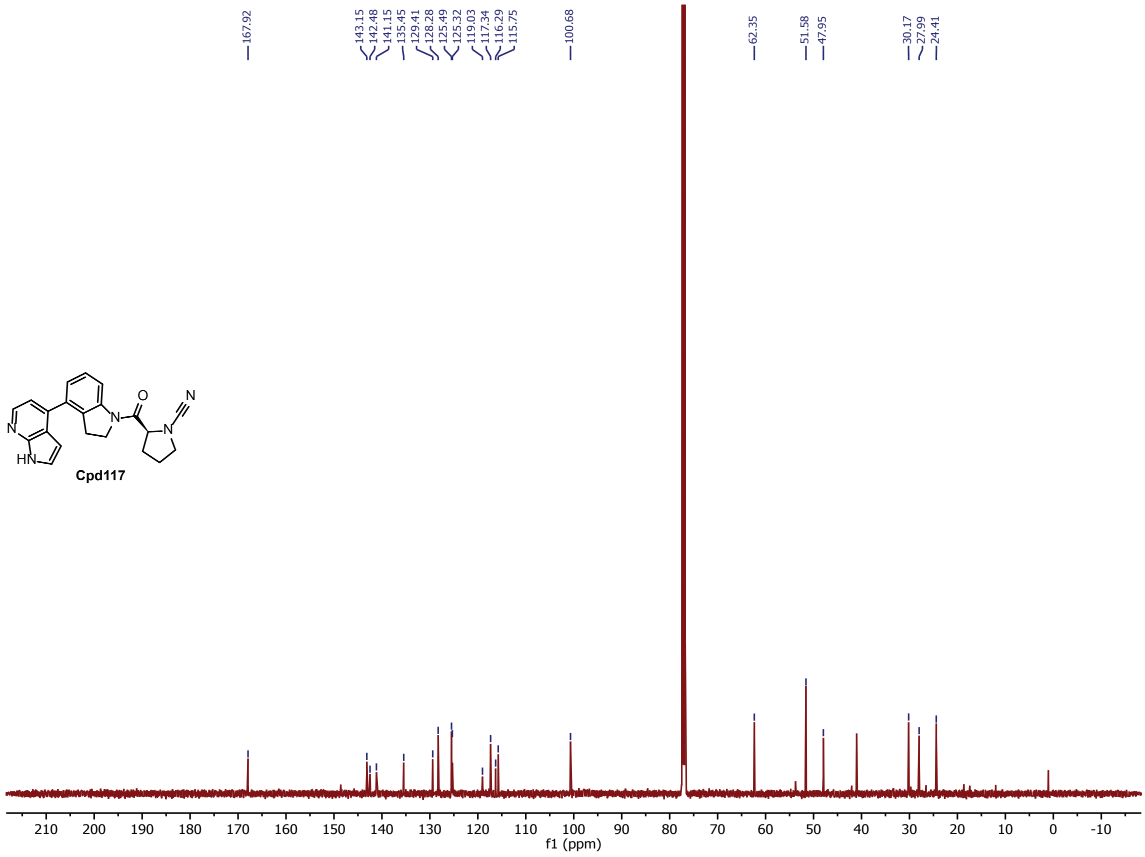

167.92
143.15
142.48
141.15
135.45
129.41
128.28
125.49
125.32
119.03
117.34
116.29
115.75
100.68
62.35
51.58
47.95
30.17
27.99
24.41
O
N
N
N
N
HN
Cpd117
210 200 190 180 170 160 150 140 130 120 110 100 90 80 70 60 50 40 30 20 10 0 -10
f1 (ppm)

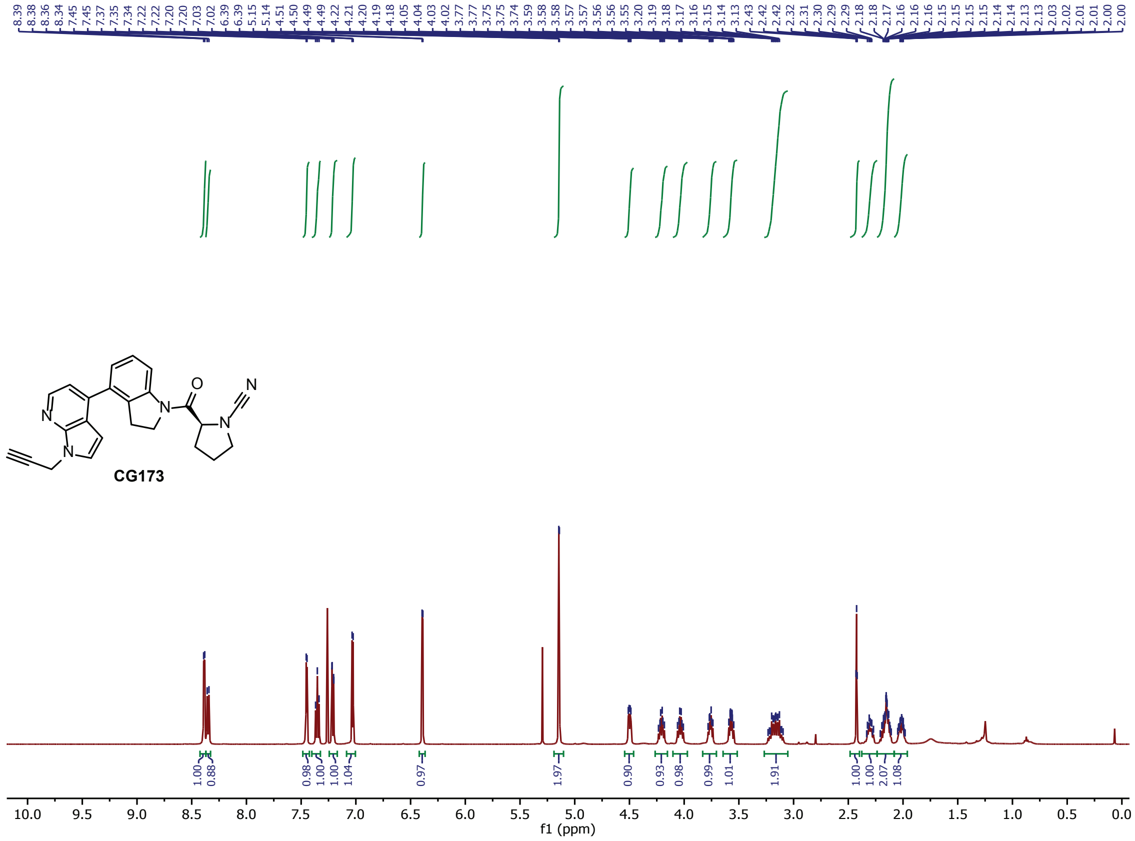
8.39 8.38 8.36 8.34 7.45 7.45 7.37 7.35 7.34 7.22 7.22 7.20 7.20 7.03 7.02 6.39 6.39 5.15 5.14 4.51 4.50 4.49 4.49 4.22 4.21 4.20 4.19 4.18 4.05 4.04 4.03 4.02 3.77 3.77 3.75 3.75 3.74 3.59 3.58 3.58 3.57 3.57 3.56 3.56 3.55 3.20 3.19 3.18 3.17 3.16 3.15 3.14 3.13 2.43 2.42 2.42 2.32 2.31 2.30 2.29 2.29 2.18 2.18 2.17 2.16 2.16 2.16 2.15 2.15 2.15 2.15 2.14 2.14 2.13 2.13 2.03 2.02 2.01 2.01 2.00 2.00
CG173
1.00 0.88 0.98 1.00 1.00 1.04 0.97 1.97 0.90 0.93 0.98 0.99 1.01 1.91 1.00 1.00 2.07 1.08
10.0 9.5 9.0 8.5 8.0 7.5 7.0 6.5 6.0 5.5 5.0 4.5 4.0 3.5 3.0 2.5 2.0 1.5 1.0 0.5 0.0
f1 (ppm)

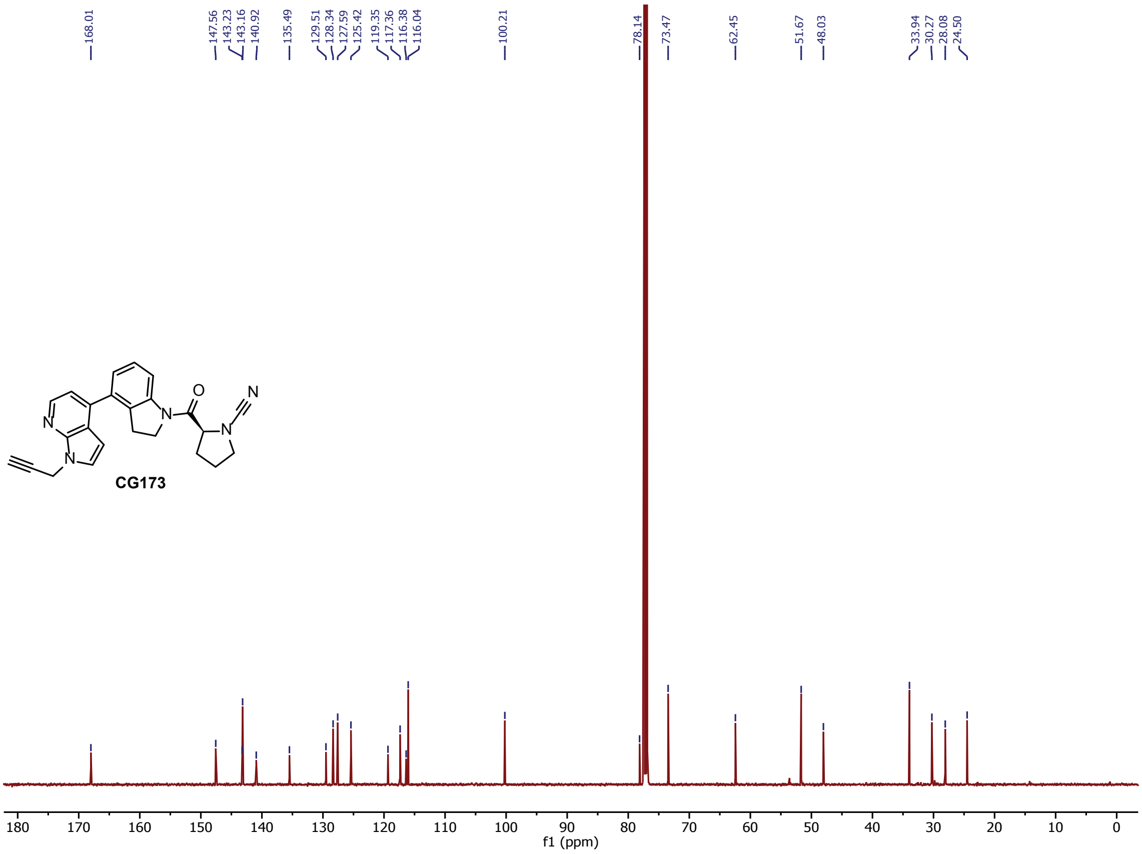
168.01
147.56 143.23 143.16 140.92
135.49
129.51 128.34 127.59 125.42
119.35 117.36 116.38 116.04
100.21
78.14 73.47
62.45
51.67 48.03
33.94 30.27 28.08 24.50
CG173
180 170 160 150 140 130 120 110 100 90 80 70 60 50 40 30 20 10 0
f1 (ppm)

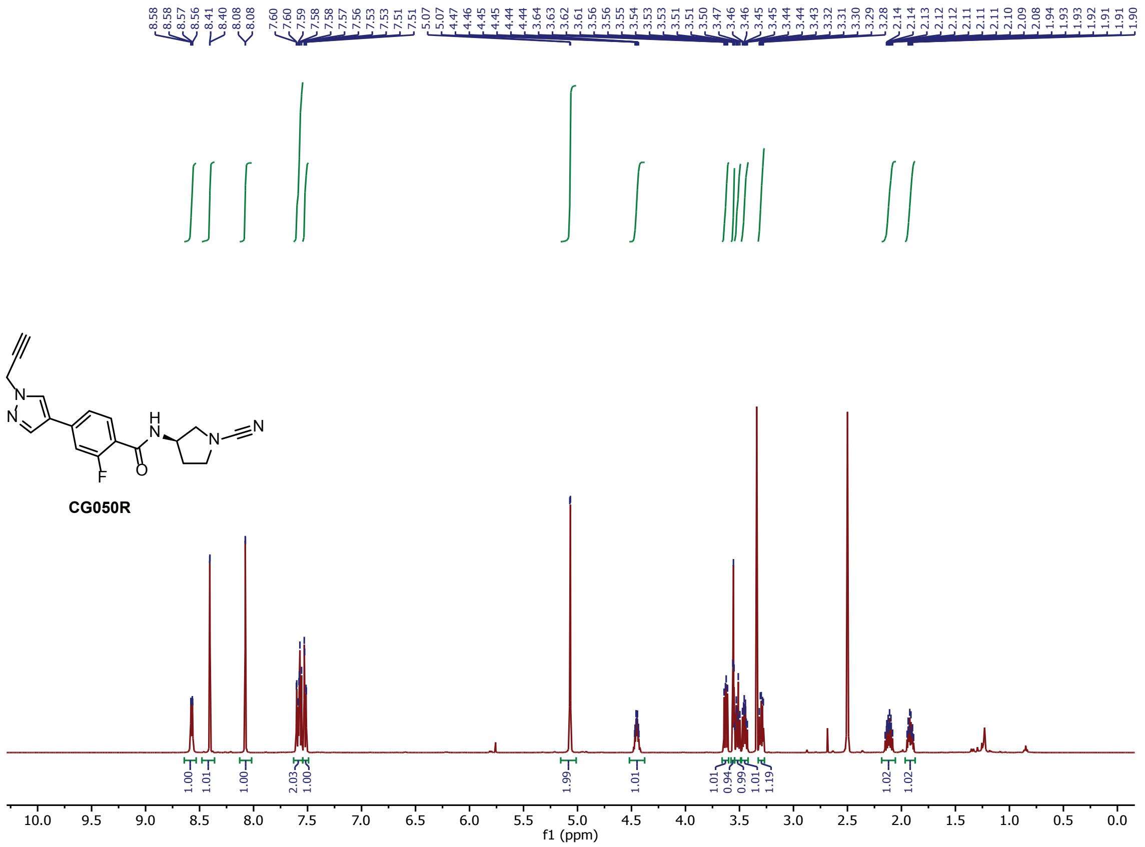
8.58 8.58 8.57 8.56 8.41 8.40 8.08 8.08 7.60 7.60 7.59 7.58 7.58 7.57 7.57 7.56 7.53 7.53 7.51 7.51 5.07 4.47 4.46 4.45 4.45 4.44 4.44 3.64 3.63 3.62 3.61 3.56 3.56 3.55 3.54 3.53 3.53 3.51 3.51 3.50 3.47 3.46 3.46 3.45 3.45 3.44 3.44 3.43 3.32 3.31 3.30 3.29 3.28 2.14 2.14 2.13 2.12 2.12 2.11 2.11 2.11 2.10 2.09 2.08 1.94 1.93 1.93 1.92 1.91 1.91 1.90
CG050R
1.00 1.01 1.00 2.03 1.00 1.99 1.01 1.01 0.94 1.01 0.99 1.01 1.19 1.02 1.02
10.0 9.5 9.0 8.5 8.0 7.5 7.0 6.5 6.0 5.5 5.0 4.5 4.0 3.5 3.0 2.5 2.0 1.5 1.0 0.5 0.0
f1 (ppm)

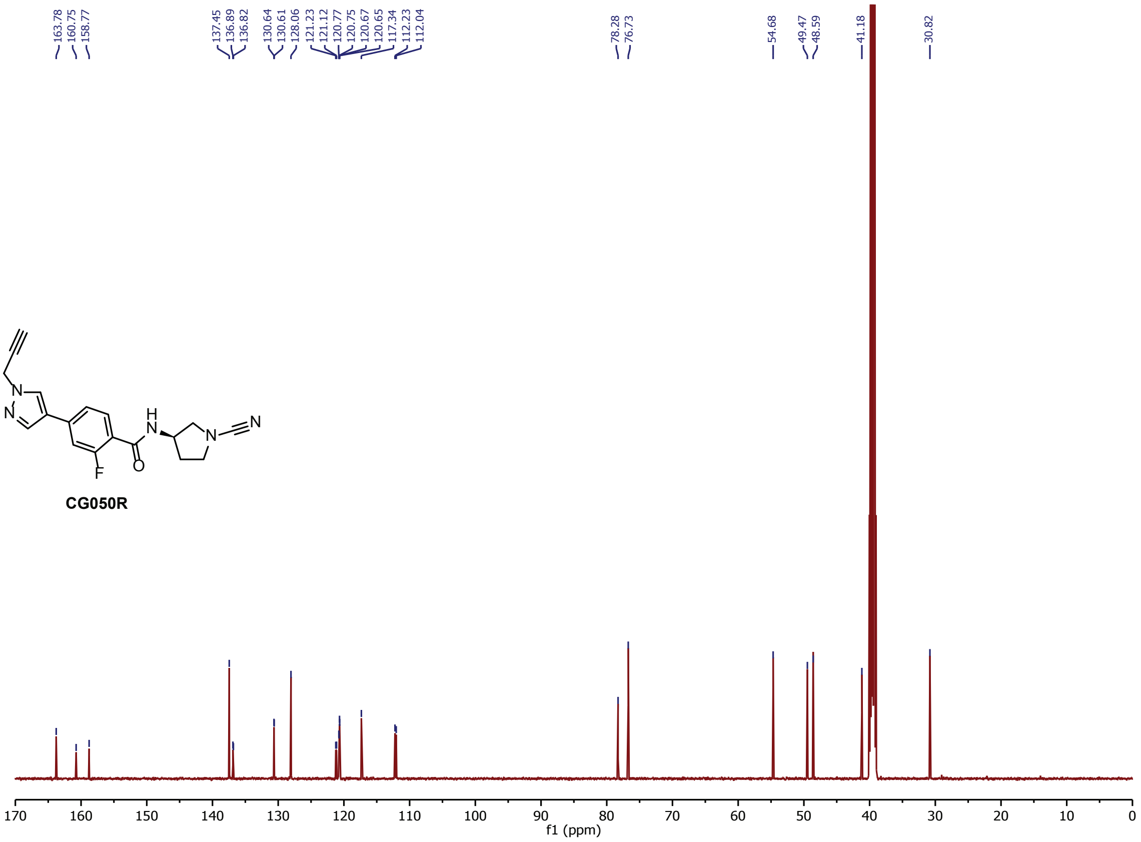
163.78 160.75 158.77 137.45 136.89 136.82 130.64 130.61 128.06 121.23 121.12 120.77 120.75 120.67 120.65 117.34 112.23 112.04 78.28 76.73 54.68 49.47 48.59 41.18 30.82
CG050R
170 160 150 140 130 120 110 100 90 80 70 60 50 40 30 20 10 0
f1 (ppm)

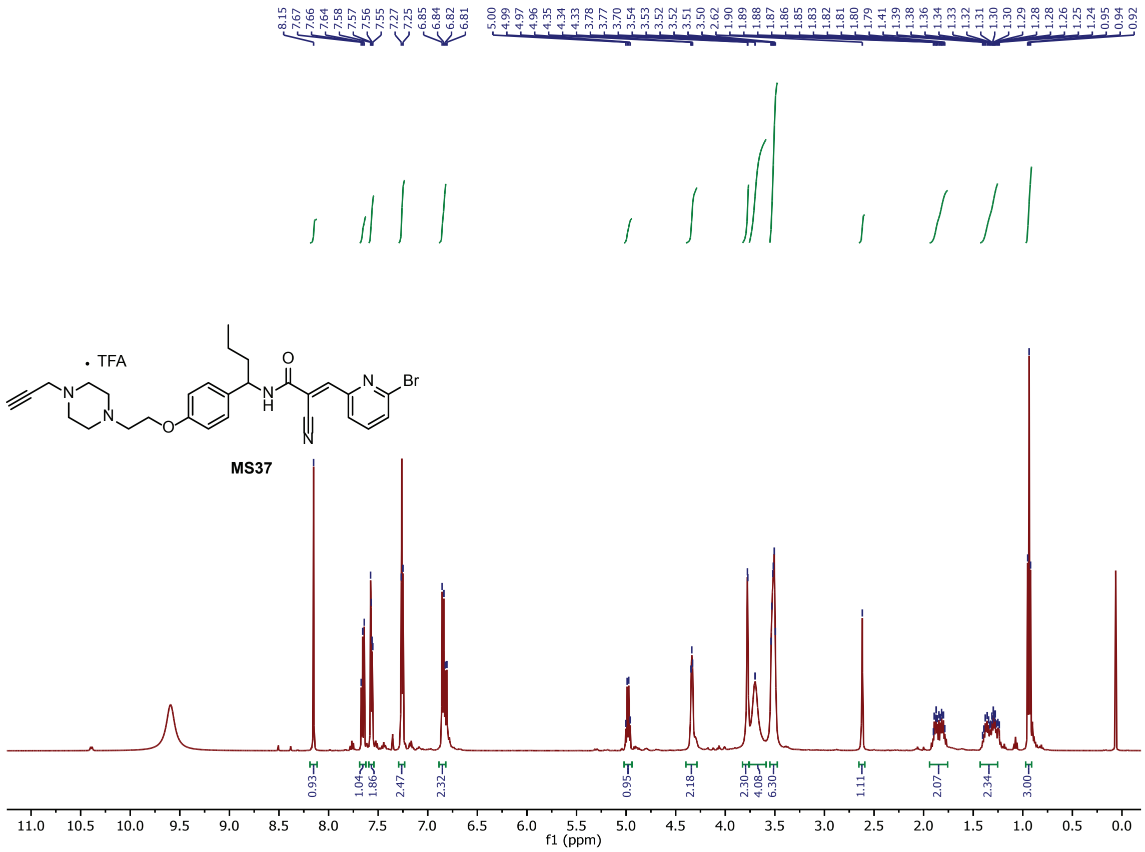
8.15 7.67 7.66 7.64 7.58 7.57 7.56 7.55 7.27 7.25 6.85 6.84 6.82 6.81 5.00 4.99 4.97 4.96 4.35 4.34 4.33 3.78 3.77 3.70 3.54 3.53 3.52 3.52 3.51 3.50 2.62 1.90 1.89 1.88 1.87 1.86 1.85 1.83 1.82 1.81 1.80 1.79 1.41 1.39 1.38 1.36 1.34 1.33 1.32 1.31 1.30 1.30 1.29 1.28 1.28 1.26 1.25 1.24 0.95 0.94 0.92
• TFA
O
N
Br
N
H
N
N
O
N
MS37
0.93 1.04 1.86 2.47 2.32 0.95 2.18 2.30 4.08 6.30 1.11 2.07 2.34 3.00
11.0 10.5 10.0 9.5 9.0 8.5 8.0 7.5 7.0 6.5 6.0 5.5 5.0 4.5 4.0 3.5 3.0 2.5 2.0 1.5 1.0 0.5 0.0
f1 (ppm)

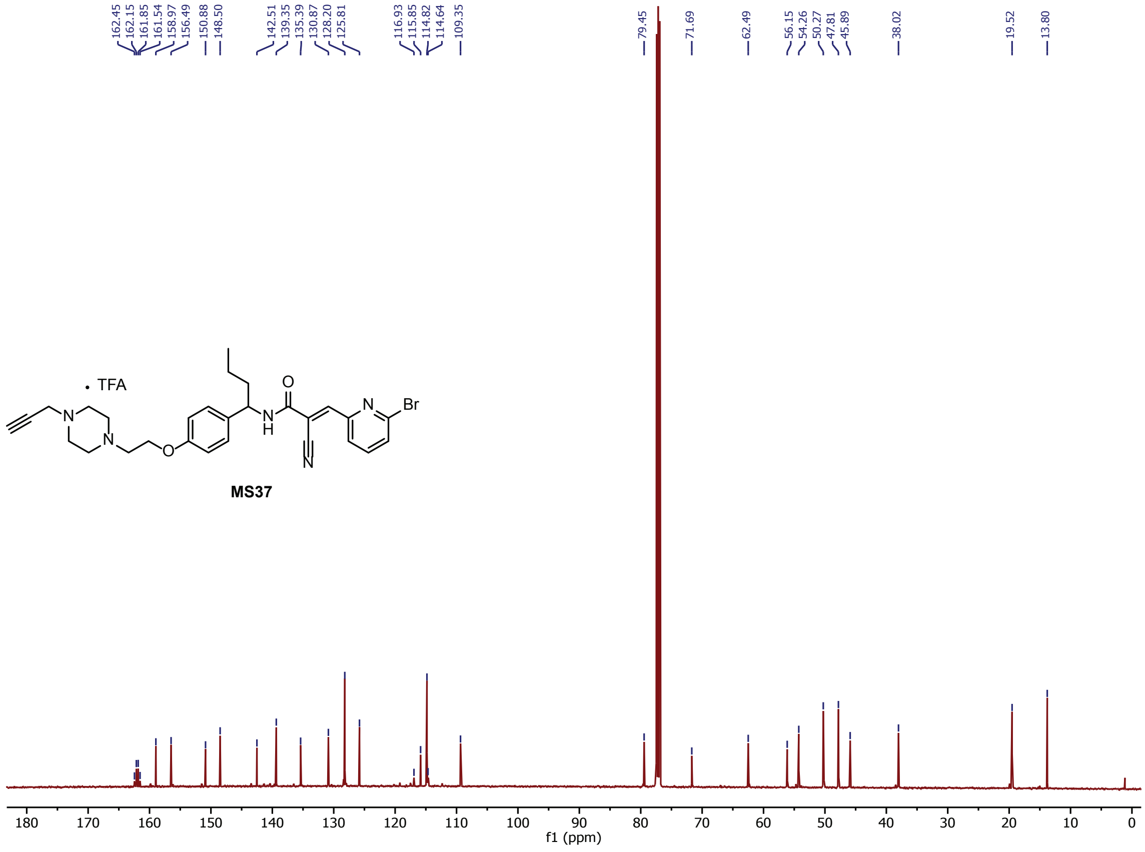
162.45 162.15 161.85 161.54 158.97 156.49 150.88 148.50 142.51 139.35 135.39 130.87 128.20 125.81 116.93 115.85 114.82 114.64 109.35 79.45 71.69 62.49 56.15 54.26 50.27 47.81 45.89 38.02 19.52 13.80
• TFA
O
N
Br
N
H
N
N
O
N
MS37
180 170 160 150 140 130 120 110 100 90 80 70 60 50 40 30 20 10 0
f1 (ppm)

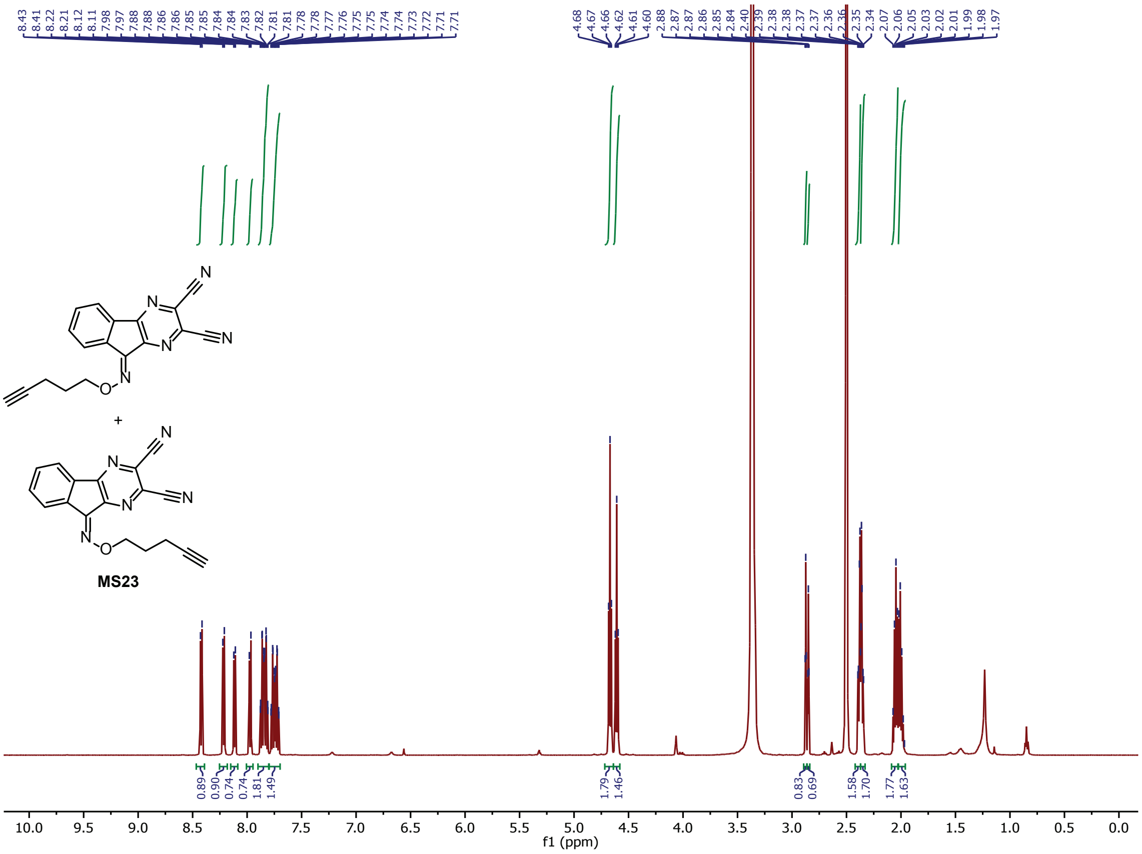
8.43 8.41 8.22 8.21 8.12 8.11 7.98 7.97 7.88 7.88 7.86 7.86 7.85 7.85 7.84 7.84 7.83 7.82 7.81 7.81 7.78 7.78 7.77 7.76 7.75 7.75 7.74 7.74 7.73 7.72 7.71 7.71
4.68 4.67 4.66 4.62 4.61 4.60 2.88 2.87 2.87 2.86 2.85 2.84 2.40 2.39 2.38 2.38 2.37 2.37 2.36 2.36 2.35 2.34 2.07 2.06 2.05 2.03 2.02 2.01 1.99 1.98 1.97
MS23
0.89 0.90 0.74 0.74 1.81 1.49
1.79 1.46
0.83 0.69
1.58 1.70
1.77 1.63
f1 (ppm)

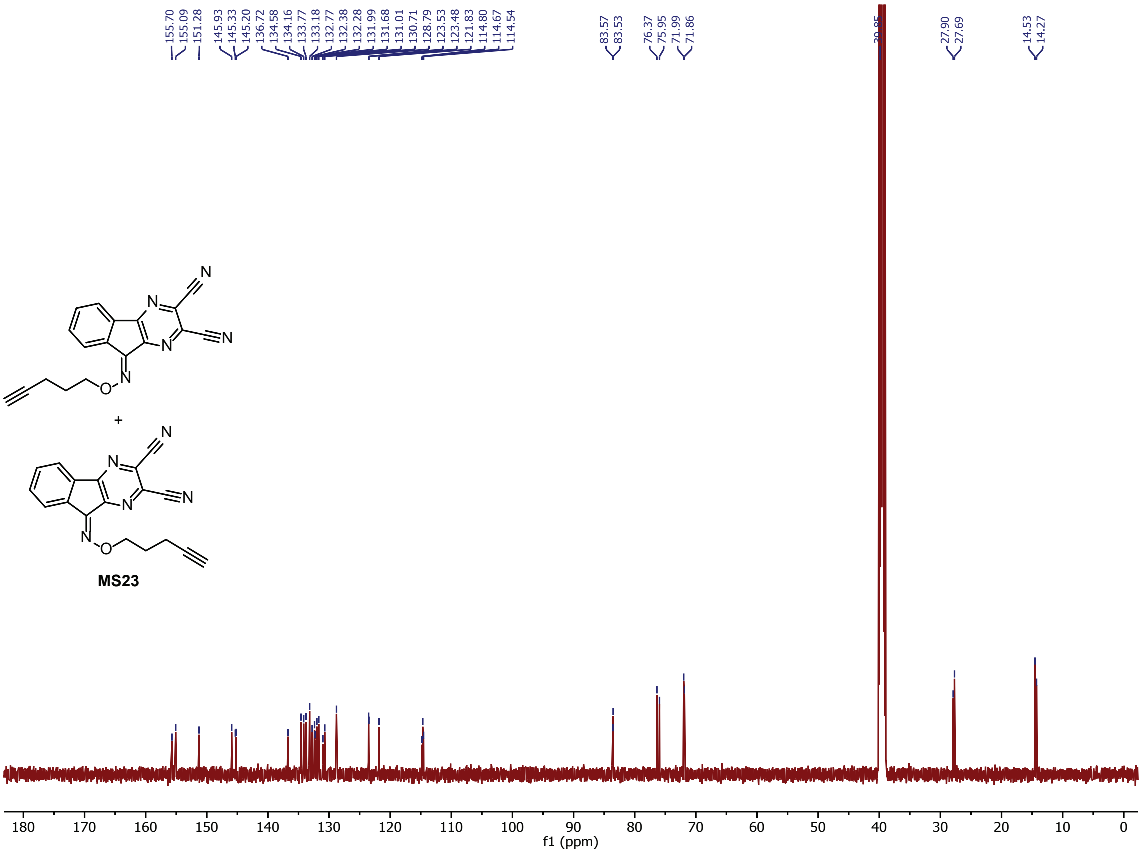
155.70 155.09 151.28 145.93 145.33 145.20 136.72 134.58 134.16 133.77 133.18 132.77 132.38 132.28 131.99 131.68 131.01 130.71 128.79 123.53 123.48 121.83 121.80 114.80 114.67 114.54
83.57 83.53
76.37 75.95 71.99 71.86
27.90 27.69
14.53 14.27
MS23
f1 (ppm)

5. Supplementary references

1. Greenspan, P.D. et al. Identification of Dipeptidyl Nitriles as Potent and Selective Inhibitors of Cathepsin B through Structure-Based Drug Design. *J Med Chem* **44**, 4524-4534 (2001).
2. Owen, D.R. et al. An oral SARS-CoV-2 M(pro) inhibitor clinical candidate for the treatment of COVID-19. *Science* **374**, 1586-1593 (2021).
3. Smith, P.A. et al. Optimized arylomycins are a new class of Gram-negative antibiotics. *Nature* **561**, 189-194 (2018).
4. Furber, M. et al. Cathepsin C inhibitors: property optimization and identification of a clinical candidate. *J Med Chem* **57**, 2357-67 (2014).
5. Boxer, M.B., Shen, M., Auld, D.S., Wells, J.A. & Thomas, C.J. A small molecule inhibitor of Caspase 1. *Probe Reports from the NIH Molecular Libraries Program [Internet]* (2010 Feb 25 [Updated 2011 Mar 3]).
6. Ward, Y.D. et al. Design and Synthesis of Dipeptide Nitriles as Reversible and Potent CathepsinS Inhibitors. *J Med Chem* **45**, 5471-5482 (2002).
7. Law, S., Andrault, P.M., Aguda, A.H., Nguyen, N.T., Kruglyak, N., Brayer, G.D. & Bromme, D. Identification of mouse cathepsin K structural elements that regulate the potency of odanacatib. *Biochem J* **474**, 851-864 (2017).
8. Guardiola, S., Prades, R., Mendieta, L., Brouwer, A.J., Streefkerk, J., Nevola, L., Tarrago, T., Liskamp, R.M.J. & Giralt, E. Targeted Covalent Inhibition of Prolyl Oligopeptidase (POP): Discovery of Sulfonylfluoride Peptidomimetics. *Cell Chem Biol* **25**, 1031-1037 e4 (2018).
9. Nabeno, M., Akahoshi, F., Kishida, H., Miyaguchi, I., Tanaka, Y., Ishii, S. & Kadowaki, T. A comparative study of the binding modes of recently launched dipeptidyl peptidase IV inhibitors in the active site. *Biochem Biophys Res Commun* **434**, 191-6 (2013).
10. Wang, Y.-H., Zhang, F., Diao, H. & Wu, R. Covalent Inhibition Mechanism of Antidiabetic Drugs—Vildagliptin vs Saxagliptin. *ACS Catalysis* **9**, 2292-2302 (2019).
11. Jilkova, A. et al. Azanitrile Inhibitors of the SmCB1 Protease Target Are Lethal to Schistosoma mansoni: Structural and Mechanistic Insights into Chemotype Reactivity. *ACS Infect Dis* **7**, 189-201 (2021).
12. Oliveira, R. et al. Tetraoxane-pyrimidine nitrile hybrids as dual stage antimalarials. *J Med Chem* **57**, 4916-23 (2014).
13. Ehmke, V., Quinsaat, J.E., Rivera-Fuentes, P., Heindl, C., Freymond, C., Rottmann, M., Brun, R., Schirmeister, T. & Diederich, F. Tuning and predicting biological affinity: aryl nitriles as cysteine protease inhibitors. *Org Biomol Chem* **10**, 5764-8 (2012).
14. Cai, J. et al. 6-Phenyl-1H-imidazo[4,5-c]pyridine-4-carbonitrile as cathepsin S inhibitors. *Bioorg Med Chem Lett* **20**, 4350-4 (2010).
15. Ramirez, Y.A., Adler, T.B., Altmann, E., Klemm, T., Tiesmeyer, C., Sauer, F., Kathman, S.G., Statsyuk, A.V., Sotriffer, C. & Kisker, C. Structural Basis of Substrate Recognition and Covalent Inhibition of Cdu1 from Chlamydia trachomatis. *ChemMedChem* **13**, 2014-2023 (2018).
16. Falgueyret, J.P., Oballa, R.M., Okamoto, O., Wesolowski, G., Aubin, Y., Rydzewski, R.M., Prasit, P., Riendeau, D., Rodan, S.B. & Percival, M.D. Novel, Nonpeptidic Cyanamides as Potent and Reversible Inhibitors of Human Cathepsins K and L. *J Med Chem* **44**, 94-104 (2001).
17. Deaton, D.N., Hassell, A.M., McFadyen, R.B., Miller, A.B., Miller, L.R., Shewchuk, L.M., Tavares, F.X., Willard, D.H., Jr. & Wright, L.L. Novel and potent cyclic cyanamide-based cathepsin K inhibitors. *Bioorg Med Chem Lett* **15**, 1815-9 (2005).
18. Boudreaux, D.A., Maiti, T.K., Davies, C.W. & Das, C. Ubiquitin vinyl methyl ester binding orients the misaligned active site of the ubiquitin hydrolase UCHL1 into productive conformation. *Proc Natl Acad Sci U S A* **107**, 9117-22 (2010).
19. Choi, D., Kim, J., Ha, S., Kwon, K., Kim, E.H., Lee, H.Y., Ryu, K.S. & Park, C. Stereospecific mechanism of DJ-1 glyoxalases inferred from their hemithioacetal-containing crystal structures. *Febs Journal* **281**, 5447-5462 (2014).
20. Heremans, I.P., Caligiore, F., Gerin, I., Bury, M., Lutz, M., Graff, J., Stroobant, V., Vertommen, D., Teleman, A.A., Van Schaftingen, E. & Bommer, G.T. Parkinson's disease protein PARK7 prevents metabolite and protein damage caused by a glycolytic metabolite. *Proceedings of the National Academy of Sciences of the United States of America* **119**(2022).
21. Leisico, F., Vieira, D.V., Figueiredo, T.A., Silva, M., Cabrita, E.J., Sobral, R.G., Ludovice, A.M., Trincao, J., Romao, M.J., de Lencastre, H. & Santos-Silva, T. First insights of

peptidoglycan amidation in Gram-positive bacteria - the high-resolution crystal structure of Staphylococcus aureus glutamine amidotransferase GatD. *Scientific Reports* **8**(2018).
22. Flohr, S., Furet, P., Imbach, P., Hommel, U., Litshcer, H.-U., Parrado, S.G., Hassiepen, U. & Zimmermann, J. 2-Cyano-pyrimidines and-triazines as cysteine protease inhibitors. *US7700605B2* (2010).
23. Eelkema, R. & Anderson, H.L. Synthesis of End-Functionalized Polyanilines. *Macromolecules* **41**, 9930-9933 (2008).
24. Arnould, J.-C., Bird, T.G., Boyle, F.T. & Blakey, D.C. Indole derivatives with vascular damaging activity. *US Patent 7,030,123* (2006).
25. Jones, A., Kemp, M.I., Stockley, M.L., Gibson, K.R., Whitlock, G.A. & Madin, A. Novel compounds. *WO2016046530A1* (2016).
26. Brodney, M.A. & Coffman, K.J. Imidazole compounds for the treatment of neurodegenerative disorders. *US Patent App. 11/971,272* (2008).
27. Kemp, M., Stockley, M. & Jones, A. Cyanopyrrolidines as dub inhibitors for the treatment of cancer. *US20180194724A1* (2018).
28. Panyain, N. et al. Correction to "Discovery of a Potent and Selective Covalent Inhibitor and Activity-Based Probe for the Deubiquitylating Enzyme UCHL1, with Antifibrotic Activity". *J Am Chem Soc* **142**, 15199 (2020).
29. Jones, A., Kemp, M., Stockley, M., Gibson, K. & Whitlock, G. 1-Cyano-pyrrolidine Compounds as USP30 Inhibitors *WO2016156816A1* (2016).
30. Brodney, M.A., Davoren, J.E., Garnsey, M.R., Zhang, L. & O'neil, S.V. Pyridine derivatives as muscarinic m1 receptor positive allosteric modulators. *US20160016907A1* (2016).
31. Donato, N.J., Talpaz, M., Peterson, L., Young, M., Showalter, H.D., Wobus, C., O'riordan, M.X.D. & Ermann, M. Deubiquitinase inhibitors and methods for use of the same. *WO2015054555A1* (2018).
32. Colombo, M., Vallese, S., Peretto, I., Jacq, X., Rain, J.C., Colland, F. & Guedat, P. Synthesis and biological evaluation of 9-oxo-9H-indeno[1,2-b]pyrazine-2,3-dicarbonitrile analogues as potential inhibitors of deubiquitinating enzymes. *ChemMedChem* **5**, 552-8 (2010).
33. Darabedian, N., Chen, T.C., Molina, H., Pratt, M.R. & Schonthal, A.H. Bioorthogonal Profiling of a Cancer Cell Proteome Identifies a Large Set of 3-Bromopyruvate Targets beyond Glycolysis. *ACS Chem Biol* **13**, 3054-3058 (2018).
