# Supplementary material for: Structural basis for specific inhibition of the deubiquitinase UCHL1
Source: Nat Commun. 2022 Oct 10;13:5950. doi: 10.1038/s41467-022-33559-4 (PMC9549030; doi:10.1038/s41467-022-33559-4)
Supplement: Supplementary file 3 — Reporting Summary [file 41467_2022_33559_MOESM3_ESM.pdf]

nature portfolio

Corresponding author(s): Malte Gersch

Last updated by author(s): Sep 9, 2022

Reporting Summary

Nature Portfolio wishes to improve the reproducibility of the work that we publish. This form provides structure for consistency and transparency in reporting. For further information on Nature Portfolio policies, see our Editorial Policies and the Editorial Policy Checklist.

Statistics

For all statistical analyses, confirm that the following items are present in the figure legend, table legend, main text, or Methods section.

n/a	Confirmed	
☐	☒	The exact sample size (n) for each experimental group/condition, given as a discrete number and unit of measurement
☐	☒	A statement on whether measurements were taken from distinct samples or whether the same sample was measured repeatedly
☐	☒	The statistical test(s) used AND whether they are one- or two-sided<br>*Only common tests should be described solely by name; describe more complex techniques in the Methods section.*
☒	☐	A description of all covariates tested
☒	☐	A description of any assumptions or corrections, such as tests of normality and adjustment for multiple comparisons
☐	☒	A full description of the statistical parameters including central tendency (e.g. means) or other basic estimates (e.g. regression coefficient) AND variation (e.g. standard deviation) or associated estimates of uncertainty (e.g. confidence intervals)
☐	☒	For null hypothesis testing, the test statistic (e.g. F, t, r) with confidence intervals, effect sizes, degrees of freedom and P value noted<br>*Give P values as exact values whenever suitable.*
☒	☐	For Bayesian analysis, information on the choice of priors and Markov chain Monte Carlo settings
☒	☐	For hierarchical and complex designs, identification of the appropriate level for tests and full reporting of outcomes
☒	☐	Estimates of effect sizes (e.g. Cohen's d, Pearson's r), indicating how they were calculated

Our web collection on statistics for biologists contains articles on many of the points above.

Software and code

Policy information about availability of computer code

Data collection	Tecan SparkControl 2.3, Agilent Openlab 2.4.0.628, Thermo Xcalibur 4.2.28.14
Data analysis	GraphPad Prism 9, ImageJ 1.53o, MaxQuant v.2.0.3.1, Perseus v.1.6.15.0, Microsoft Excel 16.60, ProMass 3.0 rev12, Phenix 1.19.2_4158, coot 0.9.6, Phaser 2.8.3, DIALS 3.1.3-ga0a48b452-release, STARANISO 3.335, Incucyte 2018A.1.6628.28170, Image Lab 6.1

For manuscripts utilizing custom algorithms or software that are central to the research but not yet described in published literature, software must be made available to editors and reviewers. We strongly encourage code deposition in a community repository (e.g. GitHub). See the Nature Portfolio guidelines for submitting code & software for further information.

Data

Policy information about availability of data

All manuscripts must include a data availability statement. This statement should provide the following information, where applicable:

- Accession codes, unique identifiers, or web links for publicly available datasets
- A description of any restrictions on data availability
- For clinical datasets or third party data, please ensure that the statement adheres to our policy

Data related to the structure of methylated UCHL1 in complex with GK13S have been deposited with the protein data bank under accession code 7ZM0. Proteomics data have been deposited with ProteomeXchange under accession codes MSV000090044 and MSV000090045. Chemical characterization data as well as uncropped gels and blots are provided in the Supplementary Information. Source data for biochemical experiments are provided with this paper. All data are available on request from the authors. Protein sequences are available through the uniport database under the following accession codes: UCHL1: P09936; UCHL3: P15374; UCHL5: Q9Y5K5; BAP1: Q92560; and PARK7: Q99497.

Field-specific reporting

Please select the one below that is the best fit for your research. If you are not sure, read the appropriate sections before making your selection.

☒ Life sciences ☐ Behavioural & social sciences ☐ Ecological, evolutionary & environmental sciences

For a reference copy of the document with all sections, see nature.com/documents/nr-reporting-summary-flat.pdf

Life sciences study design

All studies must disclose on these points even when the disclosure is negative.

Sample size	For quantitative experiments, a sample size of 3 to 6 independent experiments was chosen in line with what is the standard of the field in the molecular biosciences.
Data exclusions	Data exclusion occurred in the processing of crystallographic data as implemented in the respective software (e.g. in Phenix.Refine during the scaling of input intensities and subsequent outlier rejection according to expected intensity statistics). These processes took place completely automated as is the default in these programs and without any customization or user input.
Replication	All observations were made in at least two independent experiments, typically with technical triplicates, all with consistent results.
Randomization	Randomization was not applicable as no experiments involving humans or animals, and no experiments that might be sensitive to the order of measurement / treatment were performed.
Blinding	Blinding was not applicable as no subjective analysis (e.g. scoring) was performed.

Reporting for specific materials, systems and methods

We require information from authors about some types of materials, experimental systems and methods used in many studies. Here, indicate whether each material, system or method listed is relevant to your study. If you are not sure if a list item applies to your research, read the appropriate section before selecting a response.

Materials & experimental systems

n/a	Involved in the study	
☐	☒	Antibodies
☐	☒	Eukaryotic cell lines
☒	☐	Palaeontology and archaeology
☒	☐	Animals and other organisms
☒	☐	Human research participants
☒	☐	Clinical data
☒	☐	Dual use research of concern

Methods

n/a	Involved in the study	
☒	☐	ChIP-seq
☒	☐	Flow cytometry
☒	☐	MRI-based neuroimaging

Antibodies

Antibodies used	Primary antibodies: anti-UCHL1, 1:1000, Cell Signaling, D3T2E lot 3; anti-PARK7, 1:1000, Cell Signaling, D29E5 lot 2; anti-Tubulin, 1:4000, Sigma, T6199; anti-Hemagglutinin, 1:1000, BioLegend, 16B12 lot 13272772; anti-flag, 1:2000, Sigma, F3165 lot BW9109; anti-Ubiquitin, 1:1000, 1058 Cell Signaling, P4D1 lot 17; anti-Ubiquitin, 1:300, Santa Cruz, P4D1 sc-8017; anti-UCHL3, 1:1000, Proteintech, 12384-1-AP.<br>Secondary antibodies: anti-mouse,1:5000, Sigma, NXA931 lot 17246087; anti-rabbit, 1:5000, Sigma, GENA934 lot 17376631.
Validation	All antibodies are validated for the application of Western Blotting on human protein as per statements on the manufacturers' websites. Antibodies for UCHL1 and PARK7 were further validated by RNA interference.

Eukaryotic cell lines

Policy information about cell lines

Cell line source(s)	HEK293, HeLa, MCF-7 and PC-3 cells were obtained from the DSMZ repository (ACC 305, ACC 57, ACC 115, ACC 465). U-87 MG cells were obtained from LGC Standards (ATCC HTB-14).
Authentication	Cell lines were purchased for this study and used without authentication.
Mycoplasma contamination	Cells were regularly tested for mycoplasma contamination, with consistently negative results.

Commonly misidentified lines
(See ICLAC register)

No commonly misidentified lines were used.
