# Supplementary material for: Structural basis for specific inhibition of the deubiquitinase UCHL1
Source: Nat Commun. 2022 Oct 10;13:5950. doi: 10.1038/s41467-022-33559-4 (PMC9549030; doi:10.1038/s41467-022-33559-4)

## 2. Uncropped gels and blots

### Fig. 1d

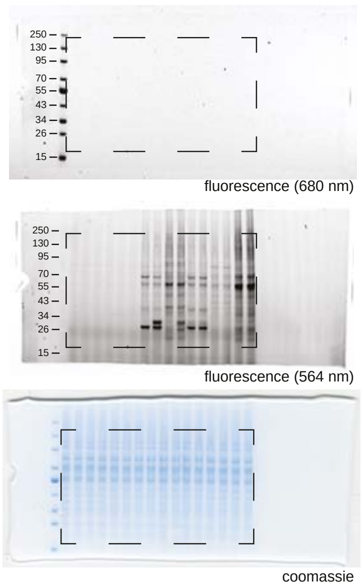


### Fig. 2b

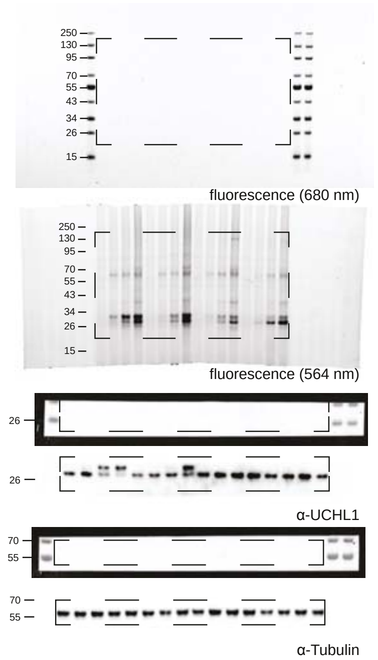


### Fig. 2c

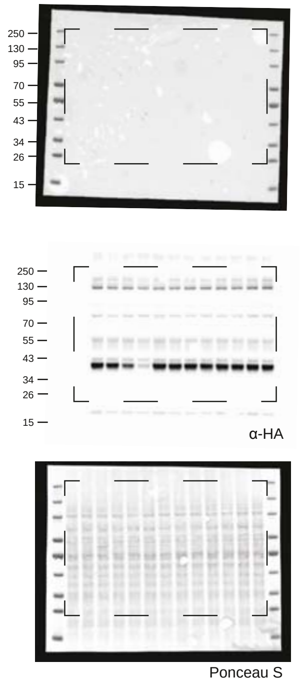


### Fig. 2f

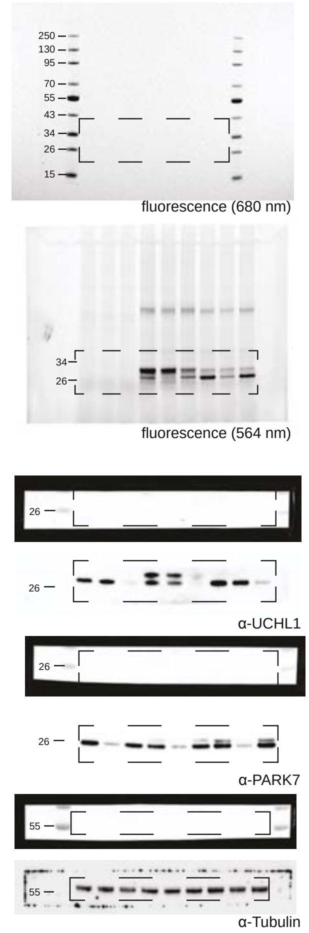


### Fig. 2g

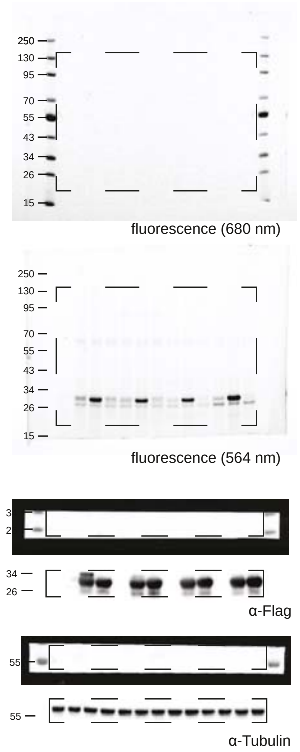


### Fig. 2h

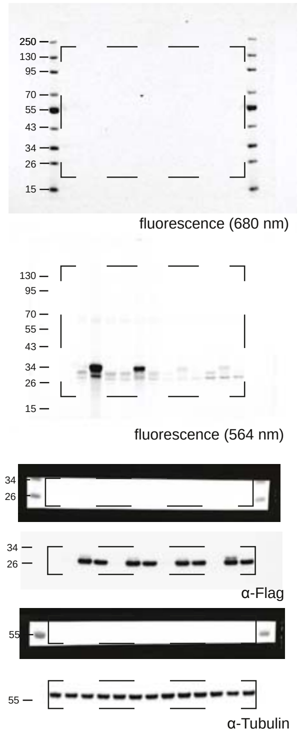

## Fig. 3e

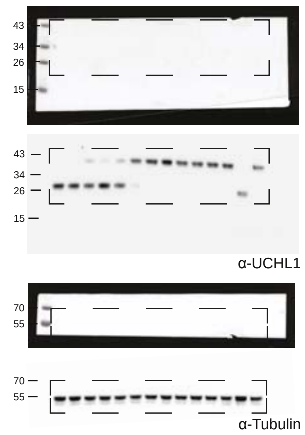


## Fig. 4b

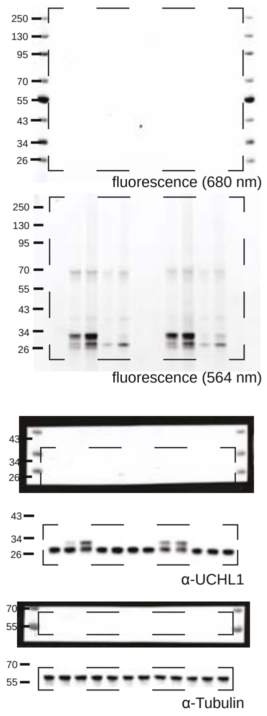


## Fig. 4d

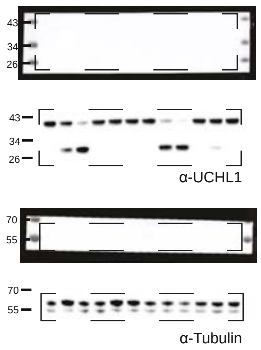


## Fig. 4e

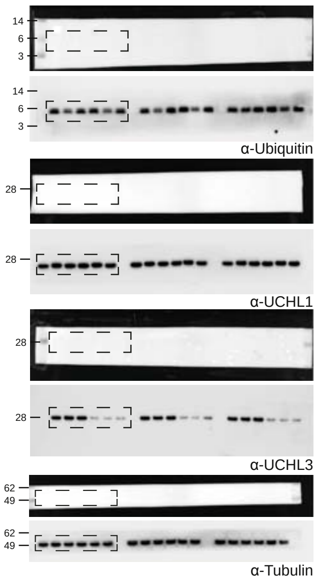

**Fig. 5k**

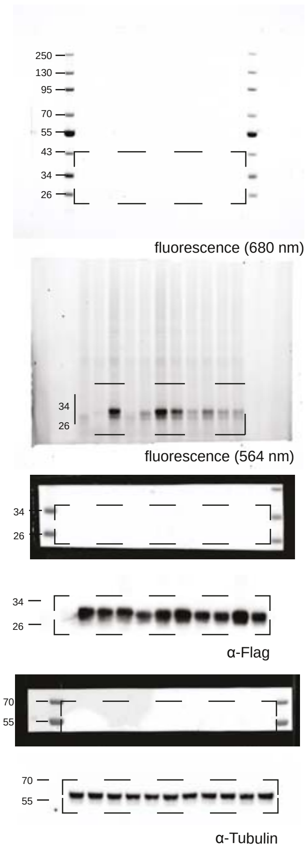
250
130
95
70
55
43
34
26
fluorescence (680 nm)
34
26
fluorescence (564 nm)
34
26
34
26
α-Flag
70
55
70
55
α-Tubulin

**Fig. 6e**

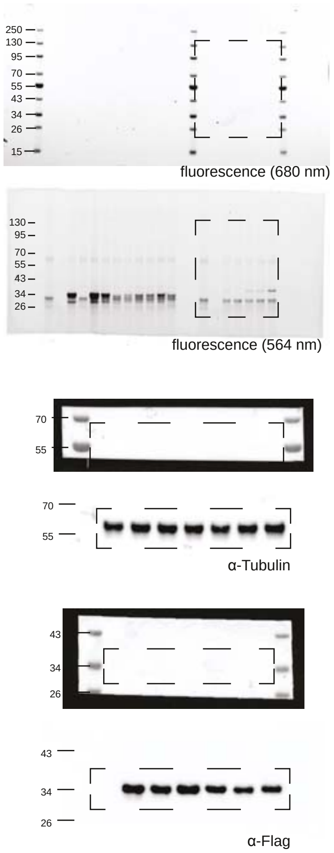
250
130
95
70
55
43
34
26
15
fluorescence (680 nm)
130
95
70
55
43
34
26
fluorescence (564 nm)
70
55
70
55
α-Tubulin
43
34
26
43
34
26
α-Flag

## Supplementary Fig. 2a

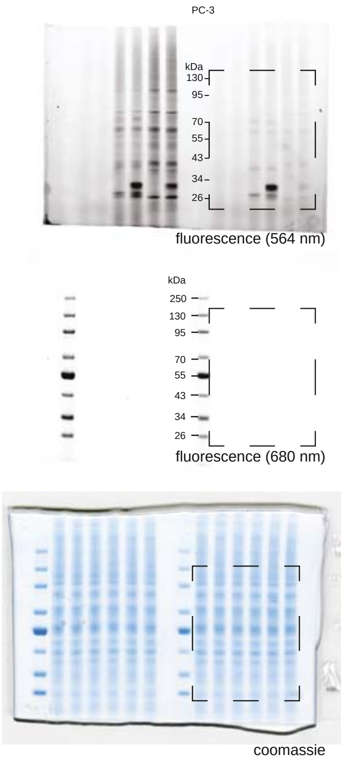


## Supplementary Fig. 2b

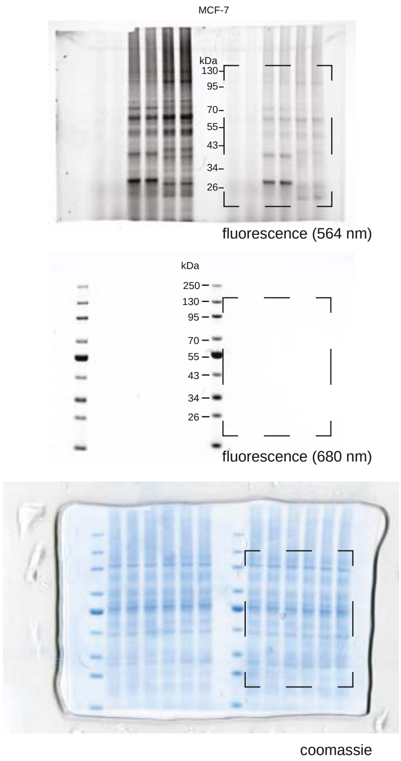


## Supplementary Fig. 2c

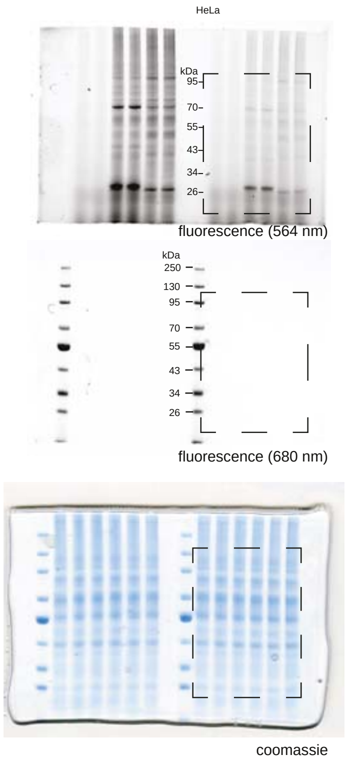


## Supplementary Fig. 2e

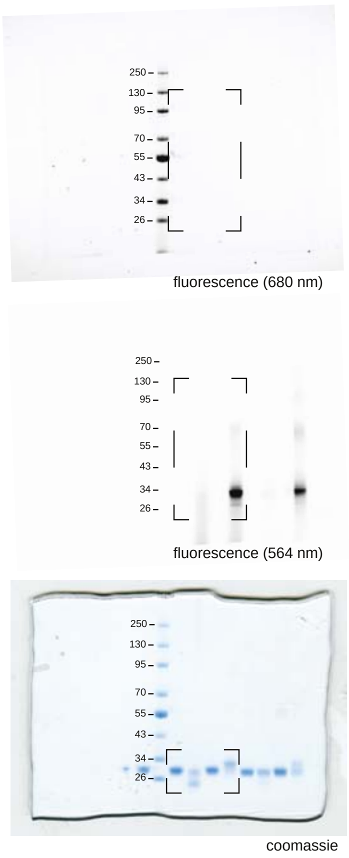


## Supplementary Fig. 5f

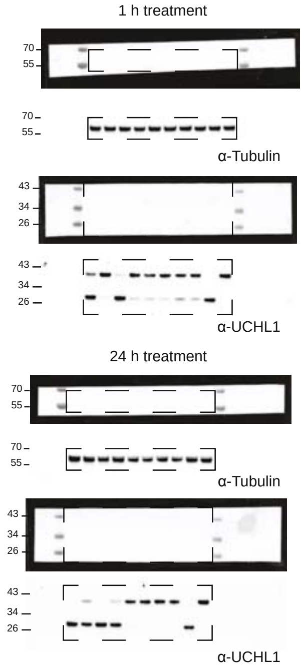


## Supplementary Fig. 7f

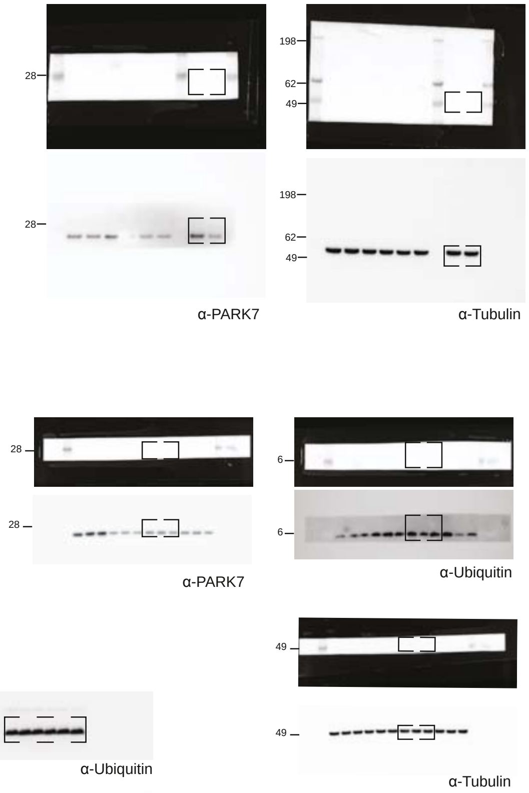


## Supplementary Fig. 7e

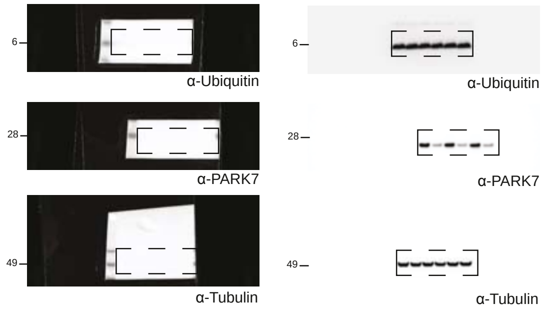

Supplement: Supplementary file 4 — Source Data Files [file 41467_2022_33559_MOESM4_ESM.zip › uncropped_blots_gels.pdf]
